# A Neuroaffirmative, Self-Determination Theory–Based Psychosocial Intervention for Adults With Attention-Deficit/Hyperactivity Disorder: Randomized Feasibility Study

**DOI:** 10.2196/69943

**Published:** 2025-10-29

**Authors:** Rebecca Elizabeth Champ, Rita Wengorovius Meneses, Marios Adamou, Warren Gillibrand, Sally Arrey, Barry Tolchard

**Affiliations:** 1 University of Huddersfield Huddersfield United Kingdom; 2 Marketing Aplicado Lda (Portugal) Lisbon Portugal; 3 University of Teeside North Yorkshire Middlesbrough United Kingdom

**Keywords:** ADHD, adult, attention-deficit/hyperactivity disorder, psychotherapy, self-determination theory, treatment

## Abstract

**Background:**

Neurodevelopmental disorders are complex and heterogeneous, creating challenges for treatment design. Multiple syndromes are associated with executive function (EF) deficits; however, theories of attention-deficit/hyperactivity disorder (ADHD) centralize a singular perspective of outcomes arising from EF impairments in adults. Deficit-based etiologies state ADHD-related EF impairments interfere with agentic self-development, perspectives that may inadvertently contribute to social stigma and influence neurotype dysphoria in ADHD identity construction. Challenges to this perspective highlight heterogeneity, context variability, the absence of a single EF deficit of origin, correlational neuroimaging data, and limited investigation into altered brain activity in ADHD research. Recommendations for psychosocial interventions primarily support cognitive behavioral therapy, which centers on a deficit-based etiology of ADHD and prioritizes symptom reduction and cognitive control of self-regulation as treatment outcomes—skills that require additional cognitive effort and may involve avoidance of emotional experiences to minimize negative affect. Transdiagnostic approaches are recommended to gain new insights into mental health challenges. Self-Determination Theory (SDT) presents a transdiagnostic approach that offers alternative outcomes by prioritizing basic psychological need satisfaction, which supports strong identity formation, motivation, and self-regulation.

**Objective:**

This study examines the feasibility and effects of an SDT-based quality-of-life therapeutic intervention for adults with ADHD.

**Methods:**

We aimed to recruit 30 participants aged 18 years or older with a confirmed diagnosis of ADHD and access to an internet connection. Participants were recruited from the Adult ADHD Clinic at the South West Yorkshire Partnership National Health Service Foundation Trust and allocated through 4-block randomization by a nonblinded researcher to either an 11-session therapeutic coaching intervention (n=11) or a control waitlist (n=9) condition. Feasibility was evaluated by pretreatment and posttreatment measurements of health-related quality of life (QoL), psychological distress, ADHD symptoms, ADHD-related QoL, self-reflection and insight, autonomous functioning, and individual outcome measures of impairment. Participants also responded to a qualitative feedback interview question on intervention value.

**Results:**

Adherence was high for both intervention completion (10/11, 91.6%) and control condition completion (9/11, 81.8%). Results showed clinically significant improvement on measures of psychological distress, specifically in the subscales of problems (*z*=0.0; *P*=.01), nonrisk (*z*=2.0; *P*=.01), functions (*z*=5.0; *P*=.02), and well-being (*z*=6.0; *P*=.03), as well as ADHD symptoms (*z*=3.0; *P*≤.01), particularly inattention (*z*=3.0; *P*≤.01), outcomes not specifically targeted by the intervention. Additional clinically significant findings of improvement in QoL, specifically in outlook subscale (*z*=21; *P*=.67), reduction of distress in problems identified in the individual outcome measure and the need for self-reflection subscale of self-reflection for the control group (*z*=1.0; *P*=.05) indicate potential positive effectiveness despite the impact of COVID-19. Positive qualitative feedback on usefulness and transferability of the intervention was provided by 90% (20/23) of participants.

**Conclusions:**

This study suggests that a randomized controlled trial of an SDT-based psychosocial intervention with nondeficit-based outcomes for adults with ADHD is feasible and recommended.

**Trial Registration:**

ClinicalTrials.gov NCT04832737; https://clinicaltrials.gov/study/NCT04832737

## Introduction

### Overview

Neurodevelopmental disorders are a category of mental health conditions defined by the *DSM-5-TR* (*Diagnostic and Statistical Manual of Mental Disorders* [Fifth Edition, Text Revision]) [1] and include attention-deficit/hyperactivity disorder (ADHD), autism spectrum disorder, neurodevelopmental motor disorders, including tic disorders, intellectual disability, communication disorders, and specific learning disorders. A shared characteristic of neurodevelopmental disorders is atypical brain development that generates impairments in cognition, communication, behavior, and motor skills. Within this heterogeneous category [2,3], the diagnostic and therapeutic approach to ADHD is overwhelmingly governed by a theoretical framework that posits executive function (EF) deficits as the origin of impairment [4-7]. This EF deficit model proposes that challenges in metacognitive, emotional, and behavioral management are critical factors impeding the sustained goal-directed activities necessary for foundational development. Success, according to these theories, is predicated on recognition and support from peers and authority figures, which are essential for achieving both self-actualization and societal functioning [5,8,9].

This dominant EF deficit model, however, faces significant and growing scientific challenges. A primary criticism is that no single EF deficit has been identified as sufficient to cause ADHD [10]. Furthermore, EF impairments themselves are not unique to the condition and can vary widely between individuals depending on context [10-12]. The neurobiological evidence is also less definitive than often assumed; neuroimaging research has not shown structural differences of a magnitude that is significant compared with controls, and the data remain correlational, unable to establish a definitive causal link [13-15]. Notably, when neuroimaging does reveal altered brain activity in ADHD participants, such as the recruitment of different response pathways, these variations are frequently categorized simply as “abnormal” rather than being explored as potentially valid alternative modes of neural organization [6,16]. This conceptual fragility calls the very foundation of current diagnostic protocols into question.

The pervasive focus on deficits has important iatrogenic consequences. The closed-label nature of the ADHD diagnosis, combined with negative narratives and stereotypes often promoted by the media, means many individuals encounter judgmental responses that alter their social treatment [17-23]. This social stigma can become internalized as ableism and neurotype dysphoria, particularly as the need for accommodations leads individuals to identify with diagnostic criteria and seek a “cure” for their inherent neurotype behaviors [24]. This process can be unintentionally reinforced by professional guidance based on the deficit model, as articulated by authorities such as Barkley [25]. This perspective frames ADHD as a chronic, incurable condition in which medication is the only effective treatment to normalize EF, functionality is dependent on external scaffolding, and any strengths are attributed to individual talent rather than recognized as potential aspects of the neurotype itself [7,26-31]. Consequently, treatment recommendations from bodies such as the National Institute for Health and Care Excellence prioritize pharmacotherapy, which, while beneficial for many, often leaves patients with significant residual symptoms and functional impairment [15,32].

The primary evidence-based nonpharmacological treatment, cognitive behavioral therapy (CBT), is also rooted in this deficit model [7,15,32,33]. While recent systematic reviews and meta-analyses show that CBT approaches can improve core symptoms and quality of life (QoL), their recommendations remain cautious due to methodological limitations such as diverse protocols, small sample sizes, and high risk of bias [34-37]. The central aim of CBT for ADHD is to strengthen cognitive abilities, increase awareness of behavior, and reframe maladaptive schemas through cognitive reappraisal [6,7]. This approach, however, presents a fundamental paradox: it demands “effortful coping” [38] and a high cognitive load to manage emotions and behavior; yet, the condition is itself characterized by effort avoidance [39] and inability to allocate sufficient cognitive effort [40]. Moreover, emerging research highlights the possibility that the ADHD neurobiological processing style may not be amenable to the reinforcement learning models upon which CBT is based [41].

Given the conceptual and practical limitations of the current paradigm, there is growing support for transdiagnostic approaches to gain new perspectives on mental health difficulties [42,43]. For ADHD specifically, recommendations are being made for transdiagnostic models due to the cross-disorder nature of EF impairments, as well as the condition’s clinical heterogeneity and context variability [3,44,45], and current theories are criticized for isolating domains of functioning and therefore lacking dimensionality and an integrative approach [46,47]. Self-Determination Theory (SDT) presents a robust, empirically based transdiagnostic framework for understanding psychopathology [21]. A mini-theory of SDT, Basic Psychological Needs Theory, posits that the satisfaction of 3 universal psychological needs—autonomy, competence, and relatedness—is essential for growth, well-being, and organismic integration. This process provides the energy for the development of an agentic self [22-24]. Research shows that the satisfaction of these needs supports mature identity formation and contributes to quality of motivation for long-term goals [48]. Conversely, the frustration or thwarting of these needs, predicts problem behaviors, increases risk of psychopathology, and can forestall identity development [21,26,27,48,49].

Recent research suggests that this experience of need frustration may function as an underlying transdiagnostic mechanism that can explain diverse forms of psychopathology and their comorbidity [50]. Some studies have examined ADHD behaviors and motivation through an SDT lens, particularly within university environments [51-56]. However, these applications have remained tethered to a deficit model, invariably using SDT as a tool to address symptom management. At the theoretical level, ADHD etiology has been interpreted as a manifestation of need frustration and impairment of internalization [6,50,57], yet no studies involving practical application of this nondeficit perspective have been published. This reveals a clear paucity of research in the area. Therefore, this study aims to address this critical gap by using SDT as an alternative theoretical foundation to explore ADHD expression and support outside the confines of the deficit-based paradigm.

### The Autonomy, Design, Awareness, Psychoeducation, and Training Integration for Sustainable Change Framework

Champ et al [6] presents a neuroaffirmative etiology of ADHD based in SDT, describing ADHD behaviors as neurobiologically altered approaches to processing and task engagement. This nondeficit model of natural ADHD behaviors based on neurodivergent neurobiological needs provides a nonstigmatizing foundation for self-regulatory functioning. Combining this model with the understanding of the polar nature of the interaction of ADHD consciousness and the environment as described in the Creative Awareness Theory [58] creates a new framework for understanding ADHD lived experience, identity formation, and self-regulation. The Creative Awareness Theory provides both practitioner and client with a positive model of unskilled attempts at self-regulation, forming an active guide to interpret existing strategies and facilitate development of awareness and self-management skills. Using this framework, it is possible to shift the focus from EF deficits and interpret ADHD psychopathology as a history of fundamental misunderstandings of ADHD motivation and engagement processes resulting in impaired internalization, need frustration, thwarting and neglect, and subsequent development of maladaptive identities, coping strategies, and need substitutes. This generates significant challenges to organismic integration by impairing connection with an authentic inner compass (AIC) [59,60]. Based in SDT, the AIC is defined as the feeling and perception of what is truly important for us—voluntary and intrinsic self-guiding preferences including values, life aspirations, interests, and goals that feel authentic and become long term as we mature. Lack of confidence and confusion regarding these preferences can impact the ability to make choices, resulting in feeling incapable of true self-direction. Research indicates that active and reflective formation of a strong sense of the AIC demonstrates an understanding of authentic core preferences expressed as agency, leading to experiences of autonomy, growth, resistance to peer pressure, and resilience [59-61]. The ADAPT (Autonomy, Design, Awareness, Psychoeducation, and Training Integration for Sustainable Change) framework aims to support individuals with ADHD using a multimodal psychotherapeutic approach to increase self-awareness of their unique neurobiology, develop their AIC, understand their basic psychological needs and needs based on their neurobiological differences, and support internalization of identity commitments. This foundation will facilitate task and environmental engagement, increase motivational activation, and feelings of confidence in their ability to design strategies to meet their needs, manage self-regulation, and develop life-crafting skills in a variety of contexts [62-64]. It is hypothesized that a neuroaffirmative ADHD treatment program that introduces the above framework will reduce symptoms, demonstrate changes in specific psychological difficulties, improve self-awareness, evidence personal experience of change, increase feelings of autonomy, and improve QoL.

### Aims and Objectives

To progress to a randomized controlled trial (RCT), it is critical to identify the most appropriate outcome measures for an SDT-based intervention for adults with ADHD. To provide clear guidance on good research conduct specifically for pilot and feasibility studies in preparation for an RCT assessing intervention or therapy effectiveness, Eldridge et al [65] developed an extension to the 2010 CONSORT (Consolidated Standards of Reporting Trials) guidelines for RCTs. This framework aligns with the United Kingdom Medical Research Council guidance on complex interventions and the National Institute for Health and Care Research definition of pilot studies. Throughout this study, the design will be referred to as a randomized feasibility study, and therefore this framework will be used as guidance. In maintaining this standard, the extension for nonpharmacologic treatment interventions to the 2010 CONSORT guidelines for reporting has been added (Multimedia Appendix 1) [66].

As RCTs are still upheld as vital for informing policy decisions [67], the call for transdiagnostic-based perspectives provides a good opportunity to offer the ADAPT framework as a novel nonpharmacological ADHD treatment approach. Therefore, this study aims to examine the feasibility, acceptability, and potential effectiveness of a randomized feasibility study evaluating a novel SDT-based program of therapeutic self-development, psychoeducation, and skills training for adults with a diagnosis of ADHD. The study objectives were to evaluate the feasibility of delivering an 11-session online self-development therapeutic intervention to a group of adults with ADHD, accounting for attrition rates of recruited participants; evaluate the acceptability of randomization to an adult ADHD population for a therapeutic self-development intervention; evaluate the acceptability of multiple measures to an adult ADHD population, including SDT-based measures for autonomy and self-reflection; evaluate the most appropriate outcome measure for the ADAPT framework for adults with ADHD; and evaluate the potential effectiveness of the ADAPT framework on ADHD symptoms, QoL, self-awareness, autonomous functioning, and personal experience of change.

## Methods

### Sample

Sample sizes for feasibility studies are much debated [68], and recommendations vary from 10-12 per group to 60-75 per group depending on study objectives [69]. We consulted with a University of Huddersfield statistician; however, this did not result in formal sample size calculation recommendations. To achieve ethical approval and with reference to the rule of thumb for a medium to large effect size (0.3<0.7), we set the sample at two groups of 10. In anticipation of dropouts, we aimed to recruit 30 participants.

### ADAPT Framework Intervention

**Figure 1:**
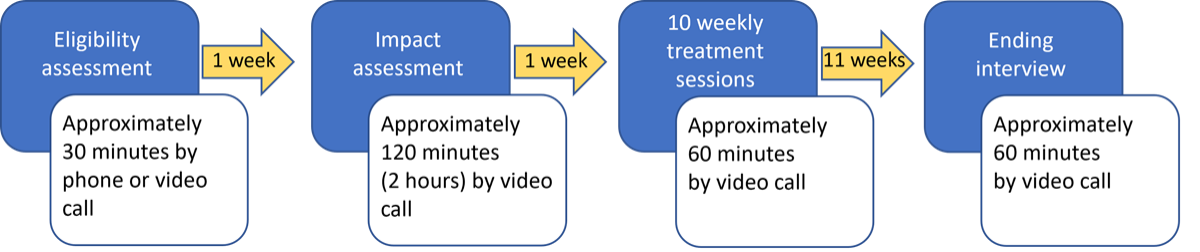
Overview of the ADAPT (Autonomy, Design, Awareness, Psychoeducation, and Training Integration for Sustainable Change) framework pilot study: assessment timeline and frequency of intervention sessions.

Eleven participants received 11 sessions of online, individually focused therapeutic coaching not currently accessible within the service (Multimedia Appendix 2). Treatment included one 2-hour assessment session exploring personal challenges and lived experience of ADHD, including a foundational section in neuroaffirmative SDT-based psychoeducation focused on neurobiological responses to environmental engagement [6], followed by ten 1-hour sessions of therapeutic coaching. Excluding session 1 of 10, which focused on time management skills for immediate and overinclusive processing styles [6], each session centralized autonomy-supportive, client-led problem identification, facilitating development of self-awareness of motivational factors in task initiation and engagement, application of context-oriented strategy development, and support for neurobiological and basic psychological needs. The researcher delivering the intervention was a psychotherapist and coach with 13 years’ experience in a specialist ADHD private practice. The intervention was supervised by a British Association for Counselling and Psychotherapy accredited psychotherapist to review casework and measure progress. Figure 1 summarizes the flow of participants through the intervention.

### Waitlist Control Group

Nine participants who met the same inclusion criteria and were assessed using the same methodology also enrolled in the therapeutic self-development intervention after a 12-week wait.

### Procedure

The design selected was a randomized, controlled study with a 1:1 allocation ratio to 2 small independent groups (control and intervention), a longitudinal design (pretreatment and posttreatment), and repeated online measures or surveys. Participants randomly assigned to intervention group and waitlist control group were assessed at pretreatment and posttreatment, and within-treatment measures were assessed during the intervention only. Assessment measures at intervention initiation and completion consisted of one measurement before and one after for both groups, while within-session measures increased the number of assessment moments to 10 for each session. Due to COVID-19 restrictions for research requiring in-person contact, all interviews, screening, data collection, and treatment sessions were conducted online using a National Health Service (NHS)–approved video platform.

### Clinical Measures

#### EQ-5D-5L

The EQ-5D-5L questionnaire [47] is a self-rated scale measuring health-related QoL in adults, used to assess treatment effect before and after treatment by measuring gains or losses in reported health status. It produces a 5-digit health state profile representing the level of reported problems on 5 dimensions of health, with lower ratings indicating better health states. This generates a health state profile, and each health state can be assigned a summary index score based on societal preference weights, or “utilities,” for that health state.

#### Clinical Outcomes in Routine Evaluation–Outcome Measure

Participants’ self-reported awareness of psychological distress was measured with the Clinical Outcomes in Routine Evaluation–Outcome Measure (CORE-OM) [70]. This 34-item scale measures 4 subscales: well-being, problems, functioning, and risk within a 7-day timeframe. Items are rated on a 6-point Likert-type sale (1=not at all to 6=most or all the time), with higher ratings indicating worse outcomes and greater psychological distress.

#### Attention-Deficit/Hyperactivity Disorder Rating Scale, Investigator-Administered

The Conners Adult ADHD Rating Scales was developed by Conners et al [71], to assess symptoms of ADHD in adults. The Attention-Deficit/Hyperactivity Disorder Rating Scale, Investigator-Administered (ADHDRS-IV-Inv) is an 18-item measure extracted from the Conners Adult Attention-Deficit/Hyperactivity Disorder Rating Scales Observer: Short Version, assessing the severity of ADHD inattentive and hyperactive-impulsive symptoms corresponding to the 18 items in the *DSM-5-TR* and providing a combined rating for severity and frequency of symptoms [72,73]. Participants are assessed on a 4-point scale (0=not at all or never to 3=very much or frequently), with severity indicated by higher ratings. The scale demonstrates good reliability, consistency, relative validity, and concurrent validity (α=.74-.95).

#### Attention-Deficit/Hyperactivity Disorder Quality of Life Scale

The Attention-Deficit/Hyperactivity Disorder Quality of Life Scale (AAQoL) is a 29-item measure assessing ADHD-related QoL areas of impact in 4 dimensions: productivity, mental health, life outlook, and relationships [74]. Participants evaluate how frequently each issue is problematic using a 5-point Likert-type scale (1=not at all or never to 5=extremely or very often), with higher ratings indicating greater problem frequency. Higher score therefore indicate poorer QoL.

#### Self-Reflection and Insight Scale

SDT highlights self-awareness as key in development of a strong AIC and the ability to identify basic psychological need frustration. The Self-Reflection and Insight Scale (SR&I) measure was used to assess individual differences in self-awareness [75]. This 20-item self-report scale consists of 3 subscales: an 8-item experience of self-reflection, an 8-item need for self-reflection, and an 8-item insight subscale. Participants rated their current state on a 7-point scale (1=strongly disagree to 7=strongly agree), with higher scores indicating greater and more frequent use of reflection skills.

#### Index of Autonomous Functioning

In SDT, autonomous behavior is experienced as self-congruent and integrated; however, the continual regulation of behavior can vary from highly autonomous, or truly self-regulated, to frequently experiencing external regulation from controlling influences [50]. Participants completed a 15-item, 5-point Likert-type scale (1=not at all true to 5=completely true) designed to measure individual differences in autonomy across 3 dimensions: authorship, control, and interest. Higher scores in any of these dimensions by inversion signifies greater autonomy.

#### Personal Questionnaire

An RCT for an intervention must demonstrate statistical significance of effectiveness for the treatment to be recommended. Practice-based evidence approaches in psychotherapy have faced challenges in this area due to the generic nature of measures, which can lack specificity and sensitivity in detecting subtle changes in an individual’s functioning; therefore, the use of a personalized outcome measure is recommended [76]. In this study, within-treatment measures consisted of the Personal Questionnaire (PQ) [77], an individualized outcome measure comparing the efficacy of the intervention across the two participant groups. The PQ is generated by participants identifying up to a maximum of 10 issues they would like to address during the intervention. These issues are identified in the assessment session, and a 7-point within-session rating of distress (1=not at all to 7=maximum possible) is completed at the start of each session throughout the treatment.

### Qualitative Measure

#### Thematic Analysis

Thematic analysis is a widely used method of qualitative research that emphasizes identifying, analyzing, and interpreting patterns of meaning in data. Participants were offered the opportunity to answer a single question about their experience during the final session of the 11-session intervention.

#### Statistical Analysis

With only 20 participants, tests suitable for analysis of small samples were used throughout, and all tests were 2-tailed with an alpha level of .05 (α=.05). Demographic and clinical characteristics were also tested for significant differences between intervention and waitlist control groups. In some cells, expected frequencies were below 5 participants; therefore, Fisher exact tests were used to test independence of experimental groups and nominal or ordinal demographics with two levels or categories. Cramer’s V was used to test the independence of experimental groups and nominal or ordinal variables with more than two groups. A Mann-Whitney U test determined the independence of experimental groups on scale variables (Table 1).

**Table 1:** ADAPT (Autonomy, Design, Awareness, Psychoeducation, and Training Integration for Sustainable Change) framework pilot study: frequency of demographic attributes per participant group and overall (N=23).

Group characteristic		Intervention, n (%)	Control, n (%)	Total, n (%)
Size		12 (52.2)	11 (47.8)	23 (100)
**Gender**				
	Male	7 (30.4)	6 (26.1)	13 (56.5)
	Female	4 (17.4)	4 (17.4)	8 (34.8)
	Transgender	1 (4.4)	1 (4.4)	2 (8.7)
	Total	12 (52.2)	11 (47.8)	23 (100)
**Age (years)**				
	<33	9 (39.1)	7 (30.4)	16 (69.6)
	>33	3 (13)	4 (17.4)	7 (30.4)
	Total	12 (52.2)	11 (47.8)	23 (100)
**Race**				
	White British	12 (52.2)	8 (34.8)	20 (87)
	Not White British	0 (0)	3 (13)	3 (13)
	Total	12 (52.2)	11 (47.8)	23 (100)
**ADHD** ^a^ **subtype**				
	Inattentive	3 (13)	4 (17.4)	7 (30.4)
	Hyperactive	1 (4.4)	1 (4.4)	2 (8.7)
	Combined	8 (34.8)	6 (26.1)	14 (60.1)
	Total	12 (52.2)	11 (47.8)	23 (100)
**Date of diagnosis**				
	Before 2020	4 (17.4)	4 (17.4)	8 (34.8)
	During or after 2020	8 (34.8)	7 (30.4)	15 (65.2)
	Total	12 (52.2)	11 (47.8)	23 (100)
**Medication**				
	None	2 (8.7)	3 (13)	5 (21.7)
	Prescribed	10 (43.5)	8 (34.8)	18 (78.3)
	Total	12 (52.2)	11 (47.8)	23 (100)

^a^ADHD: attention-deficit/hyperactivity disorder.

To determine whether the two groups differed between assessment moments over time, a nonparametric Wilcoxon signed-rank test comparing pretreatment and posttreatment data was used for all measures, except for the within-session measure, the PQ. A Friedman test was used to analyze the data collected across 10 assessment moments (T1 to T10) of the PQ, with Bonferroni-corrected multiple comparisons.

Comparing pretreatment and posttreatment results of all measures was considered in terms of reporting possible efficacy of the intervention. However, due the use of multiple measures in this study, a single general score, identified as “total,” was calculated by summing all ratings to provide an indicator participants’ performance across each of the 6 repeated measures. After establishing pretreatment values for each total variable, the normality of the distribution of these scores was tested using the Shapiro-Wilk normality test. The independence of total scores from group assignment procedures was tested with a Mann-Whitney *U* test to clarify whether groups were potentially biased in terms of pretreatment values. Finally, correlations between these variables were tested with Spearman correlations, along with Fieller, Hartley, and Pearson CIs for correlations. All analysis was conducted with SPSS (version 29; IBM Corp).

### Ethical Considerations

The ethical process for this project was reviewed by the University of Huddersfield School of Human and Health Sciences—School Research Ethics and Integrity Committee—and received Health Research Authority and Care Research Wales approval (Multimedia Appendix 3). All sessions with participants were conducted in accordance with the Ethical Framework for Good Practice set by the United Kingdom Council for Psychotherapy. The Research Ethics Committee reference for the study is 21/SC/0143. The Integrated Research Application System project ID is 291103. The trial protocol can be accessed at ClinicalTrials.gov (project ID NCT04832737).

The ethical implications for this study were considered to protect the identity of individual participants, all personally identifiable data were anonymized and will not be released. Information on confidentiality policy and anonymization of personally identifiable data were included in the consent form. Participants in the pilot study were offered a counselling agreement confirming that all details and discussions within the therapeutic relationship are confidential, unless they or anyone else are at risk of serious harm, in accordance with the United Kingdom Council for Psychotherapy Ethical Guidelines (2019; Multimedia Appendix 3).

The researcher complied with the General Data Protection Regulation, the NHS Confidentiality Code of Practice, the Computer Misuse Act (covering information security), and all local trust policies regarding the collection, storage, processing, and disclosure of personal information. All participant case records were kept in electronic form (consent forms, agreements, and interviews), and participants’ home addresses (including postcodes) and telephone numbers were stored on a secure database and spreadsheet on NHS and University computers in accordance with the Data Protection Act [78].

Participants received an initial interview invitation to discuss study participation. An information sheet detailing the purpose, activities, outcomes, and results of the study was provided, including the researcher’s contact details (name, phone number, and email address). Participants were encouraged to read the information sheet and ask questions on assessment day before signing the consent form (Multimedia Appendix 3). The voluntary nature of participation and the ability to withdraw consent at any time were emphasized during the study. All participants were offered the intervention as compensation for their time and participation.

The researcher recognized that some participants might have been recently diagnosed with ADHD. Incorporating this information into their identity and self-concept initiates a process of acceptance, which may include elements of anger and grief. The researcher was a qualified psychotherapist with 10 years of experience, and a distress policy was created as part of the research protocol. Participants were under the current clinical care of the NHS Adult ADHD Service at the South West Yorkshire Partnership NHS Foundation Trust, which was made aware of any additional support requirements if needed.

In accordance with the Data Protection Act (1998), personal data will not be retained for longer than necessary. All participant personal data, transcripts, recordings, memos, and process notes were retained for the duration of the project and were accessible only by the researcher. To submit for publication, participant data were retained to obtain permission for the study results to be published, in accordance with ethical approval. Anonymized electronic data were retained in a secure, password-protected spreadsheet and database. Hard copy data, including process notes, were stored in a locked cabinet. Videos of the intervention sessions were recorded for random review by university supervisors to ensure intervention fidelity and were destroyed upon completion of the review.

## Results

### Recruitment and Retention

**Figure 2:**
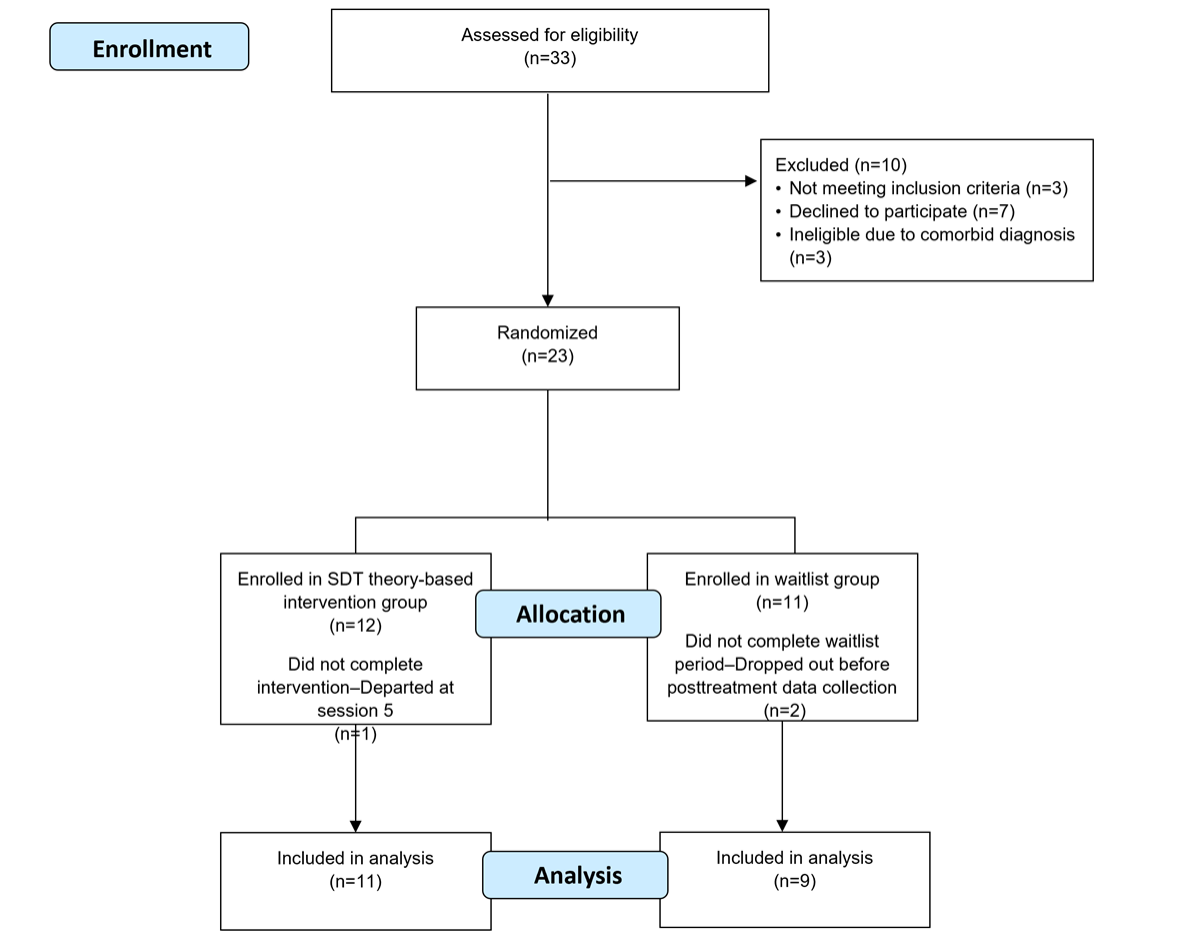
ADAPT (Autonomy, Design, Awareness, Psychoeducation, and Training Integration for Sustainable Change) framework pilot study recruitment and participation flowchart—Consolidated Standards of Reporting Trials (CONSORT) format.

Eligibility criteria consisted of age 18 years or older with a confirmed diagnosis of ADHD and access to a computer or smartphone with an internet connection. Participants with comorbid diagnosis (eg, autism, bipolar disorder, intellectual disabilities, learning difficulties, traumatic brain injury, psychosis, or Tourette syndrome), a diagnosis of substance use disorder or personality disorder, or other mental health disorders (eg, posttraumatic stress disorder and oppositional defiant disorder) were not eligible for the study. Medication was not included in the exclusion criteria, as participants were under current NHS treatment. Additionally, research indicates that multimodal treatment is recommended for adults with ADHD [10,13]. Figure 2 outlines the participant recruitment process.

Participants were recruited by staff from the Adult ADHD Clinic at the South West Yorkshire Partnership NHS Foundation Trust, which proved challenging due to high rates of ADHD comorbidity (58.4%) [79]. Recruited and randomized participants in this study consisted of 23 adult NHS patients between May 2022 and September 2022, representing 76% of the original target of 30 participants. Reasons for nonrecruitment included those who declined (n=7) and those who were discovered to be ineligible due to comorbid diagnosis of dyslexia and dyspraxia, dyslexia only, and generalized anxiety disorder (n=3). The researcher contacted and allocated a rolling entry of participants using 4-block randomization to the intervention or waitlist control over a 12-week period. The randomization was not blinded, as the researcher also delivered the intervention.

Attendance analysis showed an average of 15 weeks for participants to complete the 11-session program. The attrition rate for the full study was 13.04% of 23 participants (Figure 2). One intervention participant left at session 5, and 2 waitlist control group participants dropped out before posttreatment measure data collection without continuing on to the intervention. Correlations between demographic and clinical indicators showed that fewer dropout participants were medicated (ρ=−0.422; *P*=.05). Therefore, dropout rates suggest there may be a small risk of bias; however, the CI for this estimate was large, varying between −0.711 and −0.01 (Tables S10 and S11 in Multimedia Appendix 4).

### Measures Acceptability

This study aimed to evaluate the acceptability and accessibility of measures by participants with adult ADHD. A total of 2 participants did not complete the pretreatment or posttreatment ADHD QoL measure, leaving 10 participants in the intervention group for this measure. In the measure of autonomy Index of Autonomous Functioning (IAF), 2 participants had missing answers at pretreatment, which were resolved through imputation strategies based on each participant’s response pattern.

In the PQ, participants selected up to a maximum of 10 issues to evaluate through the intervention. Missing ratings for any issue were also absent for the entire timeframe of the intervention, indicating that the issue had not been identified for evaluation. Therefore, in both groups, the sample size decreased as the number of issues identified increased (Table 2).

One control participant only completed 9 of 10 PQ measures; therefore, an imputation strategy was used based on the participant’s response pattern (Multimedia Appendix 5).

**Table 2:** ADAPT (Autonomy, Design, Awareness, Psychoeducation, and Training Integration for Sustainable Change) framework pilot study: personal questionnaire measure of distribution of missing values and valid sample size.

Variable	Intervention			Control	
	Missing values, n	Sample size, n	Missing values, n		Sample size, n
P1-P6	0	11	≈0^a^		8
P7	0	11	1		8
P8	1	10	3		6
P9	2	9	6		3
P10	6	5	6		3

^a^One problem lacked ratings.

### Demographics and Clinical Characteristics

Participant age ranged from 20 and 56 years, with a mean of 33.35 (SD 10.1) years. In total, 13 participants identified as male, 8 as female, and 2 as transgender. Most participants (n=20) identified as White British. Clinically, 14 participants received a combined diagnosis of ADHD, 15 were diagnosed after 2020, and 18 (78.26%) were actively taking prescribed medication at the time of the study. There was no bias in the distribution of assessed demographic and clinical attributes, as group assignment was independent of Gender (Cramer V=0.038; *P*=.98), age (Mann-Whitney *U*=85.5; *P*=.24), race-nationality (Fisher exact test *P*=.09), diagnosis (Cramer V=0.13; *P*=.83), date of diagnosis (Mann-Whitney *U*=64.5; *P*=.93), and medication (Fisher exact test *P*=.64; Table 1).

Correlations between posttreatment totals and demographics were also inspected to identify any participants who might be more susceptible to the intervention. The date of the diagnosis correlated with: EQ-5D-5L total (ρ=0.573; *P*=.08), SR&I total (ρ=–0.543; *P*=.01), and ADHDRS-IV-Inv (ρ=0.553; *P*=.01). This indicates that the more recently participants had been diagnosed, the stronger were their symptoms of ADHD, and the poorer were their reflection abilities and health state.

### Quantitative Analysis for Measure Effectiveness

Table 3 summarizes the mean (SD) for each of the measures used at pretreatment and at posttreatment, namely EQ-5D-5L, CORE-OM, ADHDRS-IV-Inv, AAQoL, SR&I, and IAF. Results are also unpacked one by one for each scale in the following subsections.

**Table 3:** ADAPT (Autonomy, Design, Awareness, Psychoeducation, and Training Integration for Sustainable Change) framework pilot study: results of primary outcome measures.

Variable				Intervention group					Control group		
				Pretreatment, mean (SD)		Posttreatment, mean (SD)		Pretreatment, mean (SD)			Posttreatment, mean (SD)
**EQ-5D-5L**											
	Index value		0.70 (0.23)		0.73 (0.16)		0.78 (0.16)			0.8 (0.12)	
	Total		8.91 (2.98)		8.36 (2.20)		7.89 (2.71)			7.67 (2.24)	
**CORE-OM^a^**											
	Well-being		1.82 (0.51)		1.34 (0.36)		1.47 (0.81)			1.31 (0.89)	
	Problems		1.73 (0.53)		1.11 (0.35)		1.47 (0.62)			1.25 (0.67)	
	Functioning		1.57 (0.75)		1.12 (0.42)		1.57 (0.68)			1.19 (0.60)	
	Risk		0.08 (0.16)		0.05 (0.08)		0.13 (0.23)			0.11 (0.17)	
	Nonrisk		1.67 (0.53)		1.15 (0.27)		1.52 (0.63)			1.23 (0.63)	
	Risk to self		0.05 (0.15)		0.02 (0.08)		0.17 (0.33)			0.14 (0.22)	
	Risk to others		0.14 (0.23)		0.09 (0.20)		0.06 (0.17)			0.06 (0.17)	
	Total		1.39 (0.44)		1.21 (0.23)		1.27 (0.55)			1.32 (0.33)	
**ADHDRS-IV-Inv^b^**											
		Inattention	17.45 (5.17)		13.55 (4.12)		19.0 (4.98)			17.22 (4.82)	
		Hyperactivity	16.0 (5.6)		12.36 (5.4)		17.67 (5.1)			15.22 (5.63)	
		Total	33.45 (9.94)		25.91 (9.32)		36.67 (8.4)			32.44 (8.58)	
**AAQoL^c^**											
	Productivity		52.96 (15.27)		46.59 (13.77)		58.59 (13.72)			50.50 (8.71)	
	Mental health		40.42 (19.75)		47.08 (17.9)		39.82 (19.22)			49.54 (16.6)	
	Outlook		61.43 (16.74)		61.43 (13.66)		50.0 (11.85)			55.95 (12.11)	
	Relationships		44.50 (20.0)		54.0 (19.65)		41.67 (11.99)			45.0 (22.08)	
	Total		50.95 (9.12)		51.55 (6.52)		40.71 (4.76)			50.67 (7.43)	
**SR&I^d^**											
	Self-reflection		4.17 (1.49)		4.24 (1.32)		4.50 (0.89)			4.37 (1.24)	
	Need for self-reflection		4.91 (0.94)		4.97 (0.80)		5.57 (0.32)			5.09 (0.61)	
	Insight		3.35 (1.01)		3.71 (0.82)		3.01 (1.12)			3.61 (1.21)	
	Total		4.06 (1.04)		4.25 (0.82)		4.23 (0.55)			4.28 (0.78)	
**IAF^e^**											
	Authorship		16.04 (2.78)		17.55 (3.11)		16.89 (4.31)			16.33 (3.87)	
	Control		14.41 (4.82)		16.00 (5.48)		14.78 (3.80)			14.89 (3.85)	
	Interest		18.73 (3.74)		20.45 (4.08)		19.22 (2.91)			18.22 (4.71)	
	Total		49.18 (8.12)		54.00 (7.39)		50.89 (8.01)			49.44 (9.46)	

^a^CORE-OM: Clinical Outcomes in Routine Evaluation–Outcome Measure.

^b^ADHDRS-IV-Inv: Attention-Deficit/Hyperactivity Disorder Rating Scale, Investigator-Administered.

^c^AAQoL: Attention-Deficit/Hyperactivity Disorder Quality of Life Scale.

^d^SR&I: Self-Reflection and Insight Scale.

^e^IAF: Index of Autonomous Functioning.

#### Health-Related Quality of Life (EQ-5D-5L)

An EQ-5D-5L profile for each participant rating was generated from the data, and frequencies of every profile were determined by assessment moment and group. Only one waitlist control group profile showed a frequency higher than 1, profile 11112, which was detected in 3 participants at both pretreatment and posttreatment with no reported health state change. Findings suggest participants self-assessed their health as generally good, with slight problems in one or another single area. Wilcoxon signed-rank test results for the intervention group (W=14.0; 2-tailed significance *P*=.30) for the subscales of mobility (W=1.5; *P*>.99), self-care (W=1.5; *P*>.99), activities (W=7.0; *P*=.11), pain/discomfort (W=5.0; *P*>.99), and anxiety/depression (W=7.7; *P*>.99); and for the control group (W=4.5; 2-tailed significance *P*=.85) for the subscales of mobility (W=0.0; *P*>.99), self-care (W=1.0; *P*=.32), activities (W=3.5; *P*=.79), pain/discomfort (W=2.5; *P*=.16), and anxiety/depression (W=5.0; *P*>.99) showed no significant differences in either group between pretreatment and posttreatment results.

#### Psychological Distress (CORE-OM)

Well-being, problems, functioning, and risk as assessed by the CORE-OM showed that most participants experienced low levels of distress on every measure, and mean scores decreased overall in both groups at posttreatment. A single exception was the total score measure for the control group, which showed a small increase at posttreatment, suggesting interference in longitudinal outcomes by confounding factors. Subscales showing the highest averages, representing the greatest difficulties, were well-being and problems, whereas the lowest averages were in items assessing risk. In the intervention group, significant differences were identified in 4 subscales: well-being (*z*=6.0; *P*=.03), problems (*z*=0.0; *P*=.01), functions (*z*=5.0; *P*=.02), and nonrisk (*z*=2.0; *P*=.01); but not in risk (*z*=4.0; *P*=.71), risk to self (*z*=1.0; *P*=.66), or risk to others (*z*=6.0; *P*=.66). In the control group, only nonrisk showed significant differences across assessment moments (*z*=3.0; *P*=.04), with none observed in well-being (*z*=12.0; *P*=.39), problems (*z*=5.0; *P*=.13), functions (*z*=7.0; *P*=.07), risk (*z*=1.0; *P*=.66), risk to self (*z*=1.0; *P*=.66), or risk to others (*z*=1.5; *P*>.99).

#### ADHD Symptom Severity (ADHDRS-IV-Inv)

As assessed by the ADHDRS-IV-Inv scale, the control group reported slightly greater symptom severity at pretreatment than the intervention group. However, a decrease was registered from pretreatment to posttreatment in both groups across all subscales of this measure. Wilcoxon signed-rank tests confirmed that while both groups showed improvements at posttreatment, significant differences in favor of the intervention group were seen, with most improvements registered in the inattention scale (*z*=3.0; *P*≤.01) versus hyperactivity (*z*=2.5; *P*≤.05) and total scale (*z*=3.0; *P*≤.01). In comparison, control group improvements were noted for inattention (*z*=3.0; *P*≤.05), hyperactivity (*z*=0.0; *P*≤.05), and total (*z*=0.0; *P*≤.05) scores.

#### ADHD-related QoL (AAQoL)

The AAQoL is a self-report measure of the impact of ADHD symptoms on QoL. Analysis of intervention participants was limited to 10, as one participant did not complete the measure (ID=5). The mean (SD) observed for both groups show mid-scale responses at both assessment moments, demonstrating neither very positive nor very negative QoL. Subscale analysis indicated the intervention group increased in productivity and decreased in mental health and relationships, while outlook showed no change. The control group also increased in productivity, but averages were lower, and QoL decreased in mental health, relationships, and outlook. Wilcoxon signed-rank test showed significant differences in both groups on every scale between assessment moments, except for total score. Positive changes, indicative of increased distress, were reported in the intervention group for mental health (*z*=28.0; *P*=.02), outlook (*z*=21; *P*=.67), relationships (*z*=32.5; *P*=.04), and in total scores (*z*=31.0; *P*=.72), but not in productivity (*z*=7.0; *P*=.04). Negative changes, indicated of reduced distress, were reported in the control group for productivity (*z*=3.0; *P*=.02), whereas more distress was observed in outlook (*z*=26.0; *P*=.04), mental health (*z*=34.0; *P*=.02), relationships (Z=23.0; *P*=.48), and total scores (*z*=21.5; *P*=.62).

#### Self-Awareness (SR&I)

The SR&I is an SDT-based self-report self-awareness measure, with increases in scoring indicating positive change. Rankings were higher at intervention posttreatment across all measures, indicating overall improvement; however, higher rankings were observed in need reflection at both assessment moments, with slightly higher scores at posttreatment, while lowest rankings highlighted weakness in the insight scale. Both self-reflection and need reflection subscales showed higher results at control group baseline, indicating a decrease in self-reflection skills posttreatment. Wilcoxon signed-rank test showed no significant differences for the intervention group in subscales for self-reflection (*z*=20; *P*=.78), need for self-reflection (*z*=13.5; *P*=.93), insight (*z*=47.5; *P*=.2), or total (*z*=42.5; *P*=.4). Results for the control group showed no significant differences in subscales for self-reflection (*z*=10.0; *P*=.50), insight (*z*=35.5; *P*=.12), or total (*z*=26.0; *P*=.67), but significant differences were observed in the need for self-reflection subscale (*z*=1.0; *P*=.05), indicating worse results.

#### Sense of Autonomy (IAF)

The IAF is an SDT-based self-report measure of experiences of a sense of autonomy, with increases in scoring indicating positive change. The mean (SD) indicates that most participants reported moderately satisfactory levels of autonomy, with an average distribution of 12.5 for each subscale and 37.5 for total score. Intervention group posttreatment mean (SD) was higher for every measure, with the highest being total score at both assessment moments. Conversely, the highest averages of each pair of subscales were not always observed posttreatment in the control group, with only the control subscale demonstrating high averages. The highest averages were observed for interest, followed by authorship, and then control in both groups. Wilcoxon signed-rank text showed there were no significant differences in authorship (*z*=50.0; *P*=.13), control (*z*=39.0; *P*=.24), or interest (*z*=35.5; *P*=.41), with the closest to significance being total (*z*=52.5; *P*=.08) for the intervention group; and in authorship (*z*=20.5; *P*=.81), control (*z*=23.5; *P*=.91), interest (*z*=10.0; *P*=.25), or total (*z*=13.0; *P*=.48) for the control group.

#### Within Session Individual Outcome Measure (PQ)

The individual outcome measure analysis was performed on both groups independently, with the control group serving as a replication group undergoing the intervention. A total of 2 approaches were used for PQ data analysis. In an item-based approach, each problem was compared session by session within each group, and the widest and narrowest ranges of the scale selected by participants were compared (Table 4). This measure was useful, as participants used the full range of the scale. In terms of levels of distress, examination of the range of medians indicated higher levels of distress in the intervention group than in the control group.

**Table 4:** ADAPT (Autonomy, Design, Awareness, Psychoeducation, and Training Integration for Sustainable Change) framework pilot study: personal questionnaire measure of range and median comparison of individual outcomes.

Measure	Intervention	Control
Largest range	7 (P1T1; P8T5; P9T2)	6 (P8T7)
Narrowest range	2 (P1T8)	1 (P1T4; P4T6-P6T10; P5T2, P5T4, and P5T6-P5T10)
Highest median	6 (P2T1; P5T3 and P5T6; P7T2; P9T1)	6 (P6T1)
Lowest median	3 (P10T9)	2 (P9T8; P10T8)

**Figure 3:**
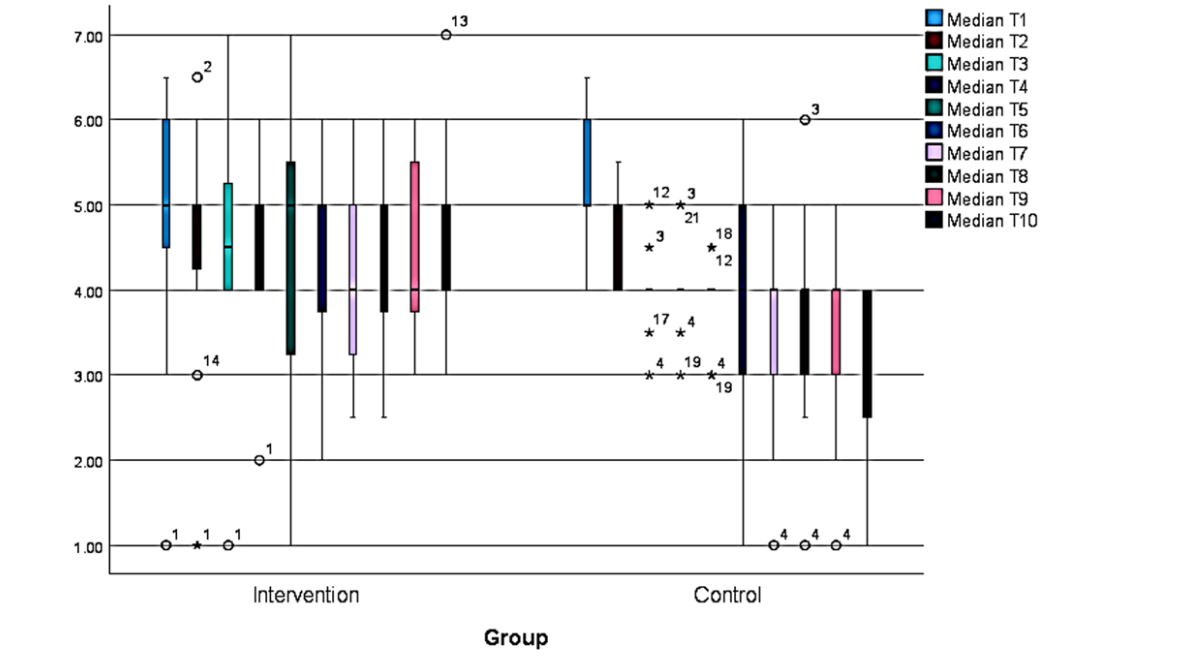
ADAPT (Autonomy, Design, Awareness, Psychoeducation, and Training Integration for Sustainable Change) framework pilot study: personal questionnaire summary of median variables across within-session assessment moments (T1-T10).

In a time-related, median-based approach, medians for the 10 moments (T1 to T10) were determined for every participant and used to compare groups. T6 functioned as the latest assessment moment at which all participants identified an issue; however, comparisons up to T10 are included. Greater levels of distress were indicated in the intervention group; however, a larger range of the intensity of distress was observed in the control group in mean (SD; Table 5). These findings remain consistent when analysis of missing data is included, despite only 5 of 11 (45%) participants contributing ratings from the intervention group and 3 of 9 (33%) from the control group. A small observable decrease in distress was seen in the control group (Figure 3).

**Table 5:** ADAPT (Autonomy, Design, Awareness, Psychoeducation, and Training Integration for Sustainable Change) framework pilot study: personal questionnaire measure of mean (SD) of medians (T1 to T10) of individual outcomes.

Moments	Intervention			Control	
	Frequency, n	Value, mean (SD)	Frequency, n		Value, mean (SD)
Median T1	11	4.82 (1.65)	9		5.39 (0.78)
Median T2	11	4.55 (1.49)	9		4.67 (0.56)
Median T3	11	4.59 (1.51)	9		4.00 (0.56)
Median T4	11	4.41 (1.02)	9		4.06 (0.64)
Median T5	11	4.41 (1.72)	9		3.89 (0.55)
Median T6	11	4.36 (1.19)	9		3.89 (1.45)
Median T7	11	4.18 (1.10)	9		3.33 (1.23)
Median T8	11	4.36 (1.42)	9		3.67 (1.44)
Median T9	11	4.50 (1.16)	9		3.39 (1.22)
Median T10	11	4.55 (1.21)	8		3.19 (1.13)

A Friedman test compared item-based ratings problem by problem (Table 6). Statistically significant differences were detected in the control group for every problem except T9 and T10, where the sample size was small (n=3). Multiple comparisons were performed to identify which pairs contributed to notable differences, with significance values adjusted using the Bonferroni correction for multiple tests. The only problems that remained significant were: P1, between T1 and T10 (*P*=.04); P2, between T2 and T9 (*P*=.02); P4, between T2 and T10 (*P*=.04); P5, between T1 and T4 (*P*=.02), T1 and T9 (*P*=.003), and T1 and T10 (*P*=.02); and P7, between T1 and T10 (*P*=.002) and T2 and T10 (*P*=.008).

**Table 6:** ADAPT (Autonomy, Design, Awareness, Psychoeducation, and Training Integration for Sustainable Change) framework pilot study: personal questionnaire results—Friedman χ² test for T1 to T10 across individual outcomes.

Problem	Intervention			Control	
	Chi-square (*df*)	*P* value	Chi-square (*df*)		*P* Value
P1 T1-T10	14.24 (9)	.11	29.33 (9)		≤.01
P2 T1-T10	5.96 (9)	.74	29.92 (9)		≤.001
P3 T1-T10	7.24 (9)	.61	24.25 (9)		.04
P4 T1-T10	10.66 (9)	.30	29.97 (9)		≤.001
P5 T1-T10	13.51 (9)	.14	41.27 (9)		≤.001
P6 T1-T10	7.07 (9)	.63	35.93 (9)		≤.001
P7 T1-T10	3.26 (9)	.95	32.4 (9)		≤.001
P8 T1-T10	10.19 (9	0.36	20.84 (9)		.01
P9 T1-T10	9.99 (9)	0.35	13.69 (9)		.13
P10 T1-10	7.23 (9)	.61	14.31 (9)		.11

**Figure 4:**
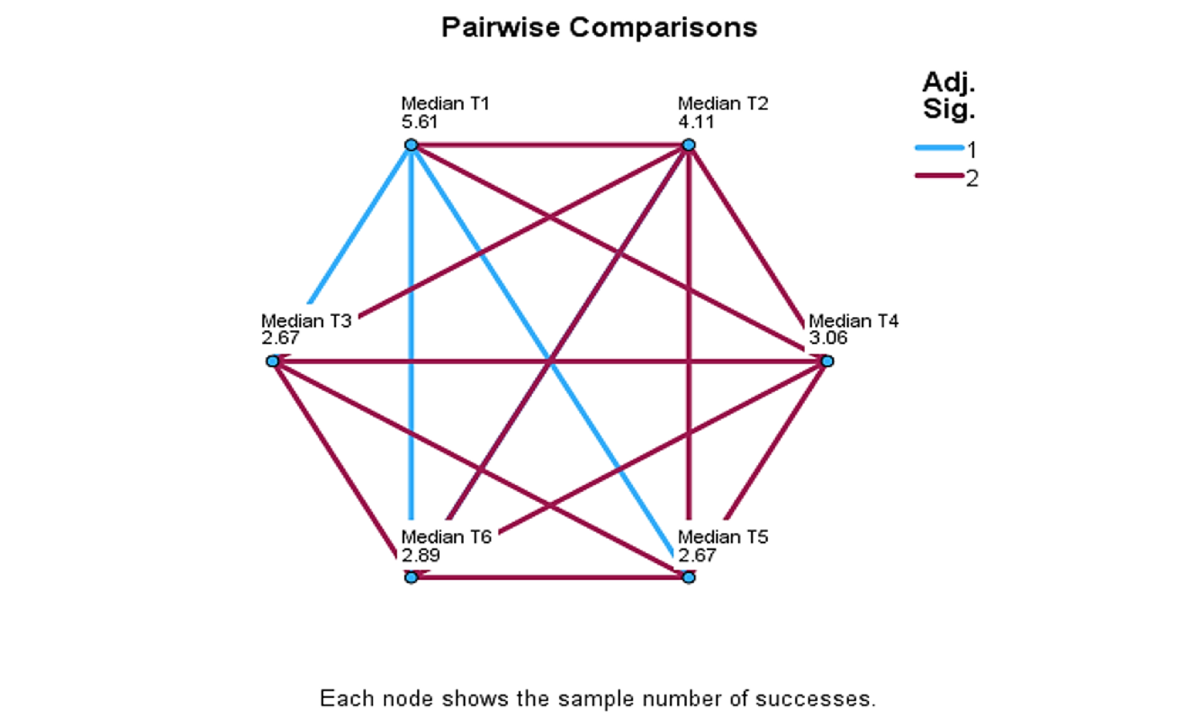
ADAPT (Autonomy, Design, Awareness, Psychoeducation, and Training Integration for Sustainable Change) framework pilot study: personal questionnaire measure of multiple comparisons for T1-T6 within-session assessment moments for the Control group.

**Figure 5:**
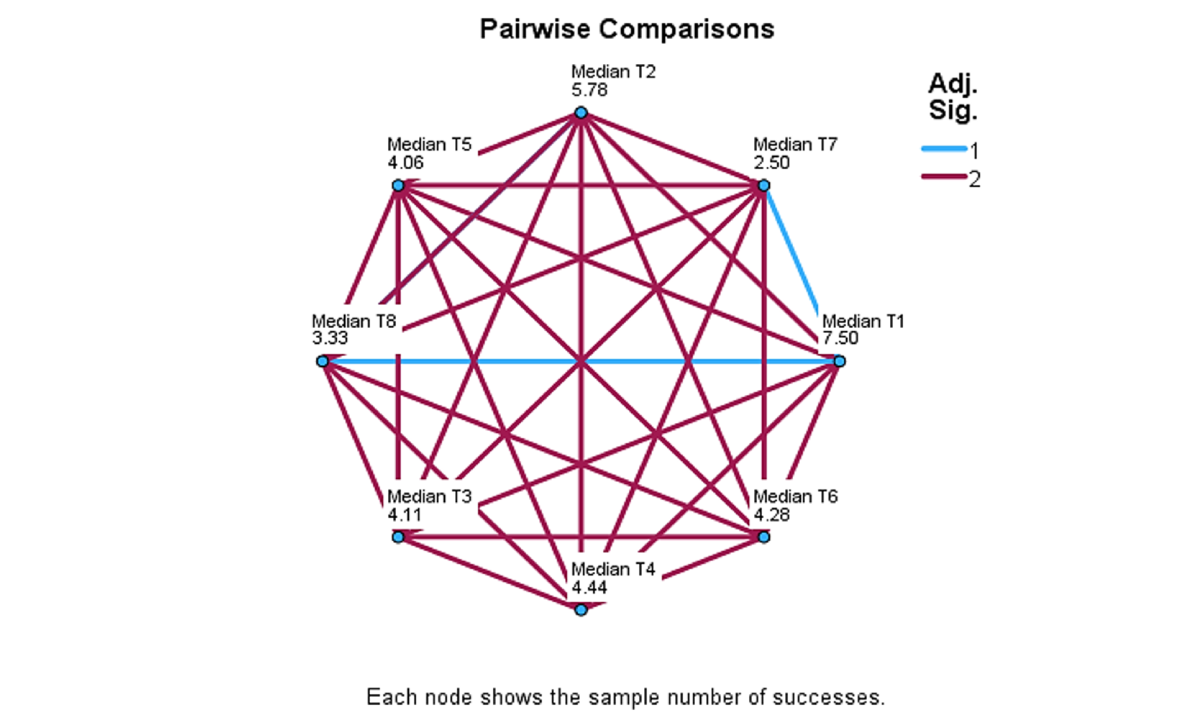
ADAPT (Autonomy, Design, Awareness, Psychoeducation, and Training Integration for Sustainable Change) framework pilot study: personal questionnaire measure of multiple comparisons for T1-T8 within-session assessment moments for the Control group.

**Figure 6:**
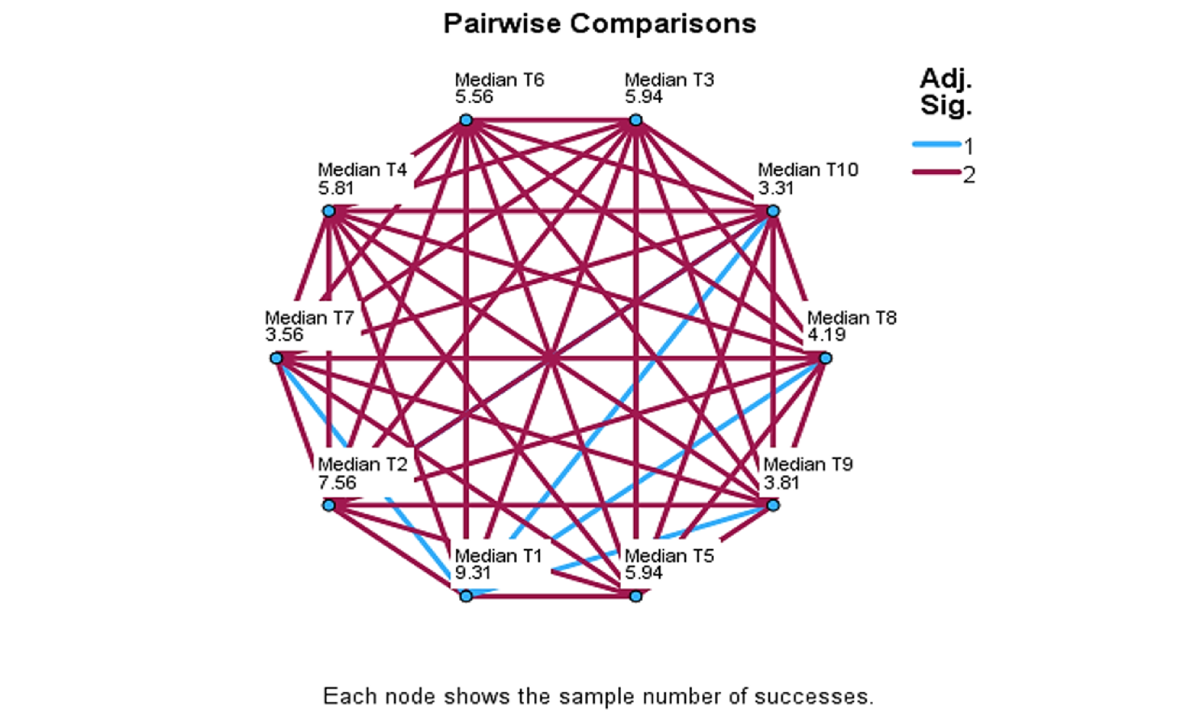
ADAPT (Autonomy, Design, Awareness, Psychoeducation, and Training Integration for Sustainable Change) framework pilot study: personal questionnaire measure of multiple comparisons for T1–T10 within-session assessment moments for the Control group.

Differences associated with assessment moments were examined using the set of time-related, median-based variables, median T1 to median T10. Significant differences were detected in the control group, but not in the intervention group, and comparisons further apart yielded stronger results. In multiple comparison analysis (Figures 4-6), significant differences detected in the control group are identified by blue lines. Overall, these results show that, when comparing T1 to T6, T1 differed significantly from T3 (*χ*²_5_=2.93; *P*=.13), T5 (*χ*²_5_=2.94; *P*=.13), and T6 (*χ*²_5_=2.72; *P*=.03). When comparing T1 to T8, these significant differences were T1 to T7 (*χ*²_7_=5.0; *P*≤.001) and to T8 (*χ*²_7_=4.17; *P*=.009). When comparing T1 to T10, significant differences were found between T1 and T7 (*χ*²_9_=5.75; *P*=.007), T8 (*χ*²_9_=5.125; *P*=.03), T9 (*χ*²_9_=5.5; *P*=.01), and T10 (*χ*²_9_=6.0; *P*=.03).

### Qualitative Analysis for Acceptability

During the final session, all participants were asked to provide intervention acceptability data by answering the question: “How did you find the experience? Was it useful?” All participants who completed the intervention agreed (Multimedia Appendix 6 for excerpts). Data analysis was conducted using thematic analysis principles outlined by Braun and Clarke [52].

#### Data Analysis

**Figure 7:**
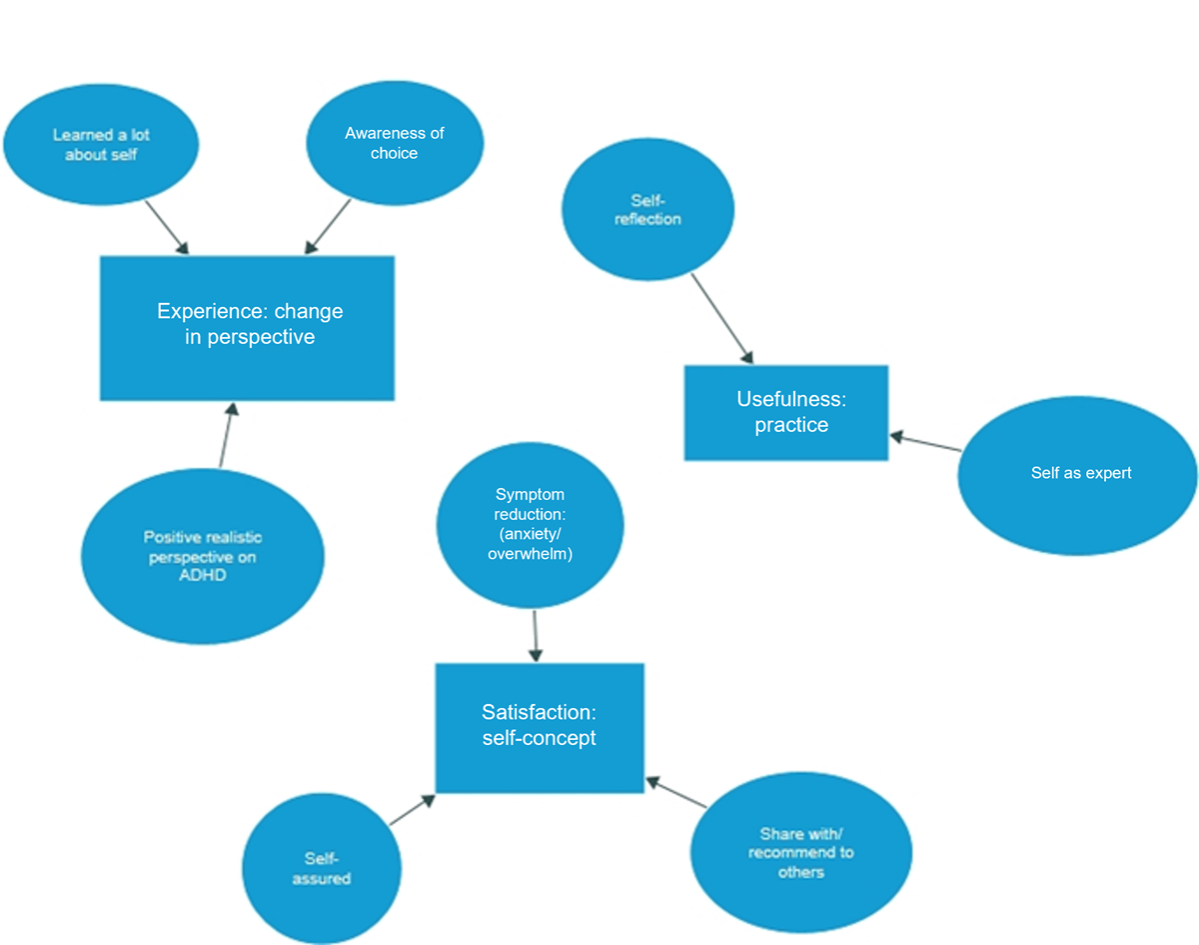
ADAPT (Autonomy, Design, Awareness, Psychoeducation, and Training Integration for Sustainable Change) framework pilot study: qualitative analysis of themes and subthemes. ADHD: attention-deficit/hyperactivity disorder.

Audio recordings of all 20 final sessions were transcribed verbatim and uploaded into NVivo (QSR International). Line-by-line coding focused on sections of the transcript referring to participants’ experiences, usefulness, and satisfaction with the intervention. A theme was identified for each aim explored in the analysis. Review and reflection were conducted midway through the data (participant 12) and again at the three-quarter stage (participant 18) to identify initial themes. The initial phase produced 55 codes from 20 interviews, which were grouped into 3 themes and 8 subthemes (Figure 7).

#### Findings

##### Experience

The emergent theme for intervention experience was a Change in Perspective. The subthemes were learning about self, positive perspective on ADHD, and awareness of choice. Participants found working the framework easy to integrate and provided new insights into their behaviors, new perspectives on options, and a sense of self-awareness around actions that altered their understanding.

Actually it's something - it's a problem I had … around the I think you call it like the disorder model of this. It was that like having an idea of the neurobiology. My first reaction to that, as a result of learning those was to be like, ‘OK well, my brain works differently and that's my excuse. I guess that's just … how things are.’ And then, ‘OK, well, I guess just things are worse for me. And I'll just have to just make peace with the fact that this is where things are.’ Whereas in the sessions we've had here, it's been more about - OK, cool. Well, let's look at that, let's actually look at overcoming some things or using the gifts that you have. I mean - It's also not the same as, like, ‘People with ADHD are able to use hyper focus’ - it's not like seeing ADHD as superpowers either. It feels like a more accurate model to me in a way. And not trying to like you know, just blow smoke at you here, but it just felt a bit more real.

Participant 7

This new perspective also supported participants’ exploration and experimentation with approaches to meet their needs, including changes in internal processes and in seeking external support through self-advocacy.

This… whole process has just been amazing because I feel like I've just understand so much and I can see what areas, maybe not need improvement, but where I need help or where I need to learn more or just do some more work and things that can get put in place, like in the workplace, which is really good…And just learning more about why I am with the way to do things, the way I need to plan things out beforehand, is just amazing. And I've learned so much about myself and it just makes things so much easier, which is really nice and like when let's say RSD [Rejection Sensitivity Dysphoria] kicks in, I know what that is and you can rationalise it so much easier than despair, which is really nice.

Participant 23

##### Usefulness

The emergent theme for intervention usefulness was Practice. The subthemes were self-expertise and self-reflection. The program was described as “useful” and “helpful” by 18 of 20 (90%) participants. Participants commented on tool development or skills learning with a focus on need satisfaction through tool adaptation, facilitating ongoing use and supporting improved functioning. Many participants highlighted self-reflection as a key factor in developing a supportive practice. A total of 2 participants found the process difficult due to extenuating circumstances, highlighting the impact of environmental support on task engagement. However, both participants identified impact factors and felt the intervention was useful despite external challenges.

No, it is going well. It's going really well. I can definitely see a change, just like I said the last time, just to thinking about it, the thought of it, you know, me sitting there and thinking about those tools that for me is really because that then gives me an option. You know, it gives me a good option and I feel like I'm not, My love, I'm being honest. I'm not overstretching myself anymore. Oh, I haven't been. For the last, oh, several weeks, I've not overstretched myself, you know.

Participant 15

##### Satisfaction

The emergent theme for intervention satisfaction was Self-concept. The subthemes that emerged were self-assured, symptom reduction, and share with/recommend to others. Participants expressed surprise when reflecting on the experience and satisfaction with gaining a positive sense of self and perspective on their capabilities. Some participants commented specifically on changes in managing anxiety and overwhelm and recommended the program to other adults with ADHD. Participants considered passing on their learned experience and helping others with similar challenges a benefit.

Honestly, I think that in itself, it just helps to keep spirits up I suppose. Instead of going into that instant self-blame. I'm kind of like, ‘Just stop right there. Let me think about this.’ I'm not just absorbing like a sponge …Like, going back and thinking of things and forgiving myself…I think there's so many things and so many times over years that I've just taken the blame or I've been doing the one in the wrong and it's me. Me, me, me and never been me in a positive light. It's always been me that is the problem. So I think one thing that I have realized is now I'm on a much better track and I'm in a much better place, and I've got all these skills for me to utilize. And this still a lot of forgiveness. Past me needs to just to be able to let go and move on and … now that I've got the knowledge, give myself the recognition that I always needed, but I wasn't able to do before.

Participant 19

## Discussion

### Overview

This research represents the first assessment of the ADAPT framework for adults with ADHD. The primary objectives of this study were to evaluate the feasibility and acceptability of an SDT-based therapeutic coaching intervention relative to a waitlist control group. Positive participation rates, low attrition, and highly positive qualitative feedback indicate that the ADAPT framework is both feasible and acceptable to participants, including the use of multiple measures with an adult ADHD population. Regarding the appropriateness of measures, ADHD QoL was the most sensitive for analyzing the intervention; however, individual outcome measures, psychological distress, and ADHD symptom severity all contributed valuable information regarding participant experiences. A total of 5 of 7 measures showed statistical significance, indicating potential effectiveness of the intervention, although not all effects were observed in the intervention group. This may be attributed to the impact of COVID-19 on improved results over time for participants during the study. An additional finding was the correlation of recent diagnosis date with stronger ADHD symptoms and poorer self-reflection and health state when comparing pretreatment and posttreatment data, suggesting that better self-understanding leads to improved functioning. The following sections will examine these results in more detail. Potential effectiveness of the intervention will also be discussed; however, in the absence of a formal power calculation, these results should be interpreted with caution.

### Feasibility and Acceptability

Participation rates were within the accepted range at 76.67%, and attrition rates were low, with 91.6% (10/11) of participants completing the Intervention and 81.8% (9/11) in the control group completing the study, indicating that the study was acceptable to participants. The significant correlation between medication use and fewer dropouts is consistent with current research and treatment recommendations for adults with ADHD, which show that psychological interventions yield better outcomes when combined with medication in multimodal treatment [10,13]. In terms of missing values, across all measures the amount was insignificant, and there were fewer missing values in the control group than in the intervention group, indicating that both inputting of responses through online forms and the number of measures required were not fatiguing and were accessible to participants. Possible reasons for missing variables are (1) known challenges with ADHD inattention on the part of the participant—each form had to be selected from a series of online links to complete the set of forms within a live session; and (2) technical issues where participants were able to complete the online form and exit or close the form without saving the data. Future studies should include steps in the protocol for the researcher to check with the participant within the session and confirm that all measures have been completed correctly, especially when delivered remotely.

Qualitative feedback from 90% (20/23) of participants indicated that the intervention as “useful” or “helpful.” Participants described a new positive perspective on ADHD and an increase in self- awareness and self-understanding. The core themes of Change in Perspective, Practice, and Self-concept support participant acceptability through learning and engagement with the intervention. Subthemes such as self-expertise, self-assured, and awareness of choice indicate that participants felt more confident in their ability to identify and support their own needs. This is further supported by the subthemes of share with/recommend to others, indicating participants not only found it useful for themselves but were also naturally sharing their experiences of the intervention with others in similar circumstances. Participant expression of increased self-awareness and understanding may be accounted for in part by the placebo effect of the therapeutic alliance [80-82]. Validation of experience and recognition of struggle for individuals who have experienced a history of stigma and rejection for differences in presentation can have a positive effect and could facilitate positive change and growth by creating a supportive environment for participants’ inherent motivation toward actualization [83].

### Measure Appropriateness

Analysis of clinical distress measures showed that individual outcome, psychological distress, and ADHD symptom severity were the most effective at capturing participant experience. Of the self-development measures, ADHD QoL was the most effective at detecting impact aimed at enhancing well-being, and self-awareness provided a unique and unexpected perspective that was highly beneficial to the analysis. As multiple measures were shown to be acceptable to participants, future research should consider additional measures focusing on self-development to evaluate the effectiveness of the intervention.

### Potential Effectiveness of the Intervention

Out of the 7 measures, health-related QoL and sense of autonomy did not show any statistically significant differences between assessment moments. The remaining scales showed statistically significant differences, but not all for the intervention group.

Of the remaining scales measuring clinical distress, both psychological distress and ADHD symptom severity showed positive change toward reducing clinical distress in both groups. However, psychological distress showed significant improvement for the intervention group in the subscales of well-being, problems, and functions, and in the ADHD symptom severity measure the improvement was slightly greater for the intervention group. These findings are particularly interesting as the ADAPT framework does not target specific ADHD symptoms but instead centralizes autonomy-support and satisfaction of the basic psychological needs of autonomy, competence, and relatedness. This is consistent with research demonstrating basic psychological needs satisfaction improves wellness, meaning, and vitality, as well as increasing internalization and intrinsic motivation [84]. Satisfaction of basic psychological needs, particularly autonomy, is seen as a vehicle for organization of the personality [22], supporting the formation of intrinsic preferences and thereby providing a foundation for the AIC [60].

The final measure of clinical distress, the individual outcome measure, showed higher levels of distress in the intervention group overall and significant change toward reducing clinical distress in the control group while participating in the intervention. This is an interesting finding, suggesting that the confounding variable in this analysis is related to COVID-19. In the United Kingdom, government COVID-19 safety restrictions were completely lifted on February 24, 2022 [85]. The first session for the first participant in the intervention group was May 27, 2022—only 2 months after restrictions were lifted. The first session for the first participant in the control group was October 17, 2022—a full 8 months after restrictions. Recent research on the impact of COVID-19 on individuals with ADHD showed negative impacts on mental health, sleep, and well-being outcomes, as well as treatment access [86]. Analysis results in this study also indicate that time was a factor in improved results in the intervention, as individual outcome measure assessment moment comparisons further apart for both groups yielded stronger results. Findings demonstrating statistically significant changes generally, and specifically in problems identified by participants in the individual outcome measure as outcomes for change, may be a result of the ADAPT framework’s semistructured focus on client-led session design and application of model elements. Traditional treatment models and recommendations are structured to focus on symptom reduction and skills development [4,25,87-91] rather than autonomy-supportive, client-led problem identification, context-oriented strategy development, and support for basic psychological needs. Therefore, it is suggested that the differences seen in the efficacy of the intervention between the two groups were due to COVID-19 negatively impacting the intervention group, and further investigation of the intervention is warranted.

In the remaining scales for self-development, ADHD QoL showed changes in both groups, suggesting that this is also reflective of the impact of COVID-19 on QoL during the intervention versus control waitlist comparison. However, the intervention group showed an improved outlook compared with the control group, suggesting that this was the result of the intervention. This finding is notable, as most interventions for adults with ADHD showing positive results in QoL are offered for up to 15 weeks or include additional booster sessions beyond 12 weeks [92-95], whereas this intervention was offered for 11 sessions and delivered just after COVID-19 restrictions were lifted. It is recommended that future studies incorporate awareness of the circumstances and environment of participants into treatment protocols and outcomes.

The final measure, self-awareness, showed significant differences only in the control group, specifically in the need for self-reflection subscale. This finding indicates that, conversely, the intervention group recognized the value and importance of self-reflection, even if they did not significantly recognize that they were actively engaging in self-reflection. It is important to note that self-reflection was highlighted as a subtheme by participants in the qualitative analysis. Indicators of this self-awareness may be attributable to treatment components focused on self-reflection, including integrative emotion regulation, reflective AIC facilitation, and reflection on barriers to task engagement. Research shows that integrative emotion regulation relates positively to openness to experience, authenticity, reflection [96], and well-being [97,98] and, like mindfulness, indicates active emotional exploration of experiences to determine their meaning and value in order to make informed choices about subsequent actions [99]. AIC facilitation research shows a firm sense of the AIC contributes to a sense of self-coherence and continuity and may reduce the need for dependency on external approval for self-esteem and the susceptibility to introjected internalization of goals or behaviors to maintain positive relationships [60,100]. Change process research highlights reflection as a principal vehicle of change, with potential application of active interest and reflection to low motivation, leading to movement toward health [73,74,101,102]. Elements of the model that prioritize autonomy support alongside increased self-awareness could help reduce discrepancies in self-concept, facilitating positive change [76,77,101]. One additional finding of interest was reported in the overall pretreatment and posttreatment comparisons, which showed a correlation between more recent ADHD diagnosis and stronger ADHD symptoms, poorer self-reflection, and poorer health state. This suggests that as participants gain a better understanding of themselves, they learn to cope with their difficulties over the long term and may benefit from treatments that support this process. This finding is of potential importance both to this study and to ADHD research overall, and further investigation is recommended.

### Limitations and Recommendations

#### Generalizability

Recruitment aims for this study were to ensure a wide age range and gender inclusion. However, the small sample size and narrow geographic location of recruitment limit generalizability to a broad population of adults with ADHD. A formal sample size was not recommended; therefore, future studies should calculate the required sample size for a definitive randomized trial. The small sample size also influenced the decision not to perform a demographic data comparison for effectiveness of the intervention; this is likewise recommended for future studies.

#### Design

Results of this study show that additional self-development measures would be both acceptable to participants and beneficial for data capture. Future investigations should address the impact of potential factors, such as the therapeutic alliance, and include more specific measures to identify change factors, such as the emotion regulation scale [103,104], AIC measures [35], the life crafting scale [38], emotion crafting scale [29], and need crafting scale [105], some of which were not available when this study was designed and received ethical approval.

Additionally, the ADAPT framework is a novel treatment intervention that has only been applied in this study in an individual therapeutic context. The model has potential to be useful in other contexts, such as group interventions, in coaching or mentoring contexts, and with other client groups, including students, parents, and partners. Future research should investigate alternative formats, group delivery, and different client groups experiencing the impact of ADHD.

#### Data Collection and Reporting

This study was limited by the number of staff and researchers available to collect data and trained to deliver the intervention. This study has some risk of bias due to the lead researcher’s role in both recruitment and assessment. Treatment outcomes were also primarily general self-report measures, and although one observer measure was used, it was administered by the primary researcher. Future studies should use blinding for assessment and analysis, and possibly also for intervention delivery, to reduce the risk of bias.

Finally, missing data impacted data analysis procedures in ways that can be improved. Adjusting the protocol to include confirmed measure completion and submission by the researcher would help reduce data collection issues. Additionally, requiring participants to identify a specific number of issues to evaluate in the PQ would reduce complexity and facilitate clarity in data analysis. Both changes are recommended for future studies.

### Conclusion

Treatment approaches for ADHD primarily focus on compensation for EF deficit–related impairment of functioning and self-regulation and provide techniques for environmental scaffolding and skills development to promote personal strengths [4,7,25,87,106]. Heterogeneity and high rates of comorbidity alongside ADHD create challenges for the design of effective nonpharmacological treatment approaches [3,16,107], and EF remains heavily debated as a core feature of ADHD etiology, particularly as EF deficits are not unique to ADHD [10-12]. SDT provides an alternative transdiagnostic approach to psychopathology, shifting the focus from EF deficits to supporting basic psychological needs as a foundation for identity development and self-regulation [23,24,29]. This study demonstrated that the ADAPT framework is a feasible and acceptable neuroaffirmative intervention for developing naturally occurring approaches and skills in identity construction, intrinsic motivation, and self-regulation for adults with ADHD. Results indicate that the intervention is not only acceptable and accessible to participants, but participants also described the capability of passing information on to others in similar circumstances as beneficial. As the intervention does not target symptom reduction outcomes, this study suggests that outcomes of well-being and self-development have a beneficial impact on symptoms in this client group. This study also suggests that context and environment have a significant impact on self-development, as indicated by the effect of COVID-19 on outcomes over time. Results indicated that the 11-session intervention was beneficial to participants during circumstances that increased distress and at a shorter duration than other interventions for adults with ADHD targeting QoL outcomes. A key finding was participant awareness of self-reflection, demonstrated in both quantitative and qualitative results. Some increases in self-awareness and the need for self-reflection may be attributable to the therapeutic alliance; however, findings highlight both self-awareness and self-reflection as promising variables for future research in this client group. Supported by the unusual pretreatment and posttreatment comparison finding of a correlation between recent diagnosis date and stronger ADHD symptoms, poorer self-reflection, and poorer health, it is suggested that diagnosis provides an alternative perspective of self and understanding of behaviors that improves outcomes naturally over time, which should be supported by interventions. Further research in randomized trials with greater statistical power is recommended to fully measure mediators and moderators of treatment effectiveness. These encouraging results may have significant practical implications for alternative approaches to the treatment of ADHD and transdiagnostic presentations of psychopathology.

## Appendix

Multimedia Appendix 1 CONSORT 2010 Checklist for RCT and nonpharmacological interventions.

Multimedia Appendix 2 ADAPT Framework Therapist Guide.

Multimedia Appendix 3 Ethical approvals and participant forms.

Multimedia Appendix 4 Statistics report.

Multimedia Appendix 5 Measures.

Multimedia Appendix 6 Thematic analysis quotes.

## Abbreviations

AAQoL: Attention-Deficit/Hyperactivity Disorder Quality of Life Scale
ADAPT: Autonomy, Design, Awareness, Psychoeducation, and Training Integration for Sustainable Change
ADHD: attention-deficit/hyperactivity disorder
ADHDRS-IV-Inv: Attention-Deficit/Hyperactivity Disorder Rating Scale, Investigator-Administered
AIC: authentic inner compass
CBT: cognitive behavioral therapy
CONSORT: Consolidated Standards of Reporting Trials
CORE-OM: Clinical Outcomes in Routine Evaluation–Outcome Measure
DSM-5-TR: Diagnostic and Statistical Manual of Mental Disorders (Fifth Edition, Text Revision)
EF: executive function
IAF: Index of Autonomous Functioning
NHS: National Health Service
PQ: personal questionnaire
QoL: quality of life
RCT: randomized controlled trial
SDT: Self-Determination Theory
SR&I: Self-Reflection and Insight Scale
