# Correction: A Neuroaffirmative, Self-Determination Theory–Based Psychosocial Intervention for Adults With Attention-Deficit/Hyperactivity Disorder: Randomized Feasibility Study

**DOI:** 10.2196/87679

**Published:** 2025-12-03

**Authors:** Rebecca Elizabeth Champ, Rita Wengorovius Meneses, Marios Adamou, Warren Gillibrand, Sally Arrey, Barry Tolchard

**Affiliations:** 1 University of Huddersfield Huddersfield United Kingdom; 2 Marketing Aplicado Lda (Portugal) Lisbon Portugal; 3 University of Teeside North Yorkshire Middlesbrough United Kingdom

In “A Neuroaffirmative, Self-Determination Theory–Based Psychosocial Intervention for Adults With Attention-Deficit/Hyperactivity Disorder: Randomized Feasibility Study” the authors noted one error.

In the originally published manuscript, an incorrect image appeared for Figure 1. The originally published image is available in Multimedia Appendix 1.

In the corrected manuscript, Figure 1 appears as follows:

**Figure 1:**
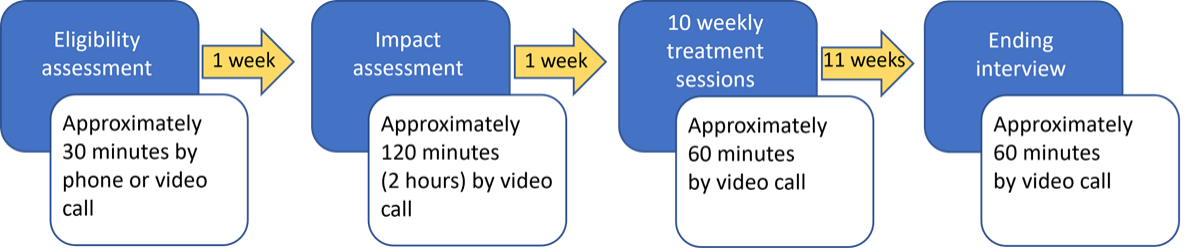
Overview of ADAPT Framework pilot study assessment time and frequency of intervention sessions.

The correction will appear in the online version of the paper on the JMIR Publications website on December 4th 2025, together with the publication of this correction notice. Because this was made after submission to PubMed, PubMed Central, and other full-text repositories, the corrected article has also been resubmitted to those repositories.

## Appendix

Multimedia Appendix 1 Originally published figure.

