# Supplementary material for: A Neuroaffirmative, Self-Determination Theory–Based Psychosocial Intervention for Adults With Attention-Deficit/Hyperactivity Disorder: Randomized Feasibility Study
Source: JMIR Form Res. 2025 Oct 29;9:e69943. doi: 10.2196/69943 (PMC12612647; doi:10.2196/69943)
Supplement: Multimedia Appendix 1 [file formative_v9i1e69943_app1.docx]

# **Supplemental Material 1: CONSORT 2010 checklist for reporting a pilot or feasibility study including nonpharmacologic treatment extension**

| **Section/Topic** | **Item No.** | **Checklist item** | **Reported on page No.** |
| --- | --- | --- | --- |
| **Title and abstract** | | |  |
|  | 1a | Identification as a pilot or feasibility randomised trial in the title  NP*: description of the experimental treatment, comparator, care providers, centers, and blinding status. | 1 |
|  | 1b | Structured summary of pilot trial design, methods, results, and conclusions (for specific guidance see CONSORT abstract extension for pilot trials) | 2 |
| **Introduction** | | | |
| Background and objectives | 2a | Scientific background and explanation of rationale for future definitive trial, and reasons for randomised pilot trial | 3-7 |
|  | 2b | Specific objectives or research questions for pilot trial | 10 |
| **Methods** | | | |
| Trial design | 3a | Description of pilot trial design (such as parallel, factorial) including allocation ratio | 11 |
|  | 3b | Important changes to methods after pilot trial commencement (such as eligibility criteria), with reasons | N/A |
| Participants | 4a | Eligibility criteria for participants  NP*: Eligibility criteria for centres and training and skill level of those performing the interventions | 15  8-9 |
|  | 4b | Settings and locations where the data were collected | 8-9 |
|  | 4c | How participants were identified and consented | 10, 16 |
| Interventions | 5a | The interventions for each group with sufficient details to allow replication, including how and when they were actually administered  NP*: Precise details of both experimental treatment and comparator | Supplement 2  8-9 |
|  | 5b | NP*: Description of the different components of the interventions and, when applicable, description of the procedure for tailoring the interventions to individual participants | 8-9 |
|  | 5c | NP*: Details of how the interventions were standardized | 8-9 |
|  | 5d | NP*: Details of how adherence of care providers with the protocol was assessed or enhanced | N/A |
| Outcomes | 6a | Completely defined prespecified assessments or measurements to address each pilot trial objective specified in 2b, including how and when they were assessed | 11-13 |
|  | 6b | Any changes to pilot trial assessments or measurements after the pilot trial commenced, with reasons | N/A |
|  | 6c | If applicable, prespecified criteria used to judge whether, or how, to proceed with future definitive trial | N/A |
| Sample size | 7a | Rationale for numbers in the pilot trial  NP*: When applicable, details of whether and how the clustering by care providers or centers was addressed | 8  N/A |
|  | 7b | When applicable, explanation of any interim analyses and stopping guidelines | N/A |
| **Section/Topic** | **Item No.** | **Checklist item** | **Reported on page No.** |
| **Randomisation** | | | |
| Sequence  generation | 8a | Method used to generate the random allocation sequence  NP*: When applicable, how care providers were allocated to each trial group | 16  N/A |
|  | 8b | Type of randomisation(s); details of any restriction (such as blocking and block size) | 16 |
| Allocation  concealment  mechanism | 9 | Mechanism used to implement the random allocation sequence (such as sequentially numbered containers), describing any steps taken to conceal the sequence until interventions were assigned | 16 |
|  |  |  |  |
| Implementation | 10 | Who generated the random allocation sequence, who enrolled participants, and who assigned participants to interventions | 16 |
| Blinding | 11a | If done, who was blinded after assignment to interventions (for example, participants, care providers, those assessing outcomes) and how  NP*: Whether or not those administering co-interventions were blinded to group assignment | N/A  16 |
|  | 11b | If relevant, description of the similarity of interventions | N/A |
|  | 11c | NP*: If blinded, method of blinding and description of the similarity of interventions | N/A |
| Statistical methods | 12 | Methods used to address each pilot trial objective whether qualitative or quantitative  NP*: When applicable, details of whether and how the clustering by care providers or centers was addressed | 11-13  N/A |
| **Results** | | | |
| Participant flow (a diagram is strongly recommended) | 13a | For each group, the numbers of participants who were approached and/or assessed for eligibility, randomly assigned, received intended treatment, and were assessed for each objective  NP*: The number of care providers or centers performing the intervention in each group, and the number of patients treated by each care provider or in each center | 16-17  N/A |
|  | 13b | For each group, losses and exclusions after randomisation, together with reasons | 16 |
| Recruitment | 14a | Dates defining the periods of recruitment and follow-up | 16 |
|  | 14b | Why the pilot trial ended or was stopped | 16 |
| Baseline data | 15 | A table showing baseline demographic and clinical characteristics for each group  NP*: When applicable, a description of care providers (case volume, qualification, expertise, etc.) and centers (volume) in each group | 19  N/A |
| Numbers analysed | 16 | For each objective, number of participants (denominator) included in each analysis. If relevant, these numbers should be by randomised group | 18-33 |
|  |  |  |  |
| **Section/Topic** | **Item No.** | **Checklist item** | **Reported on page No.** |
| Outcomes and estimation | 17 | For each objective, results including expressions of uncertainty (such as 95% confidence interval) for any  estimates. If relevant, these results should be by randomised group | 15, 18-33 |
|  |  |  |  |
| Ancillary analyses | 18 | Results of any other analyses performed that could be used to inform the future definitive trial | 18-33 |
|  |  |  |  |
| Harms | 19a | All important harms or unintended effects in each group (for specific guidance see CONSORT for harms) | 9-11 |
|  | 19b | If relevant, other important unintended consequences | N/A |
| **Discussion** | | | |
| Limitations | 20 | Pilot trial limitations, addressing sources of potential bias and remaining uncertainty about feasibility | 36-37 |
| Generalisability | 21 | Generalisability (applicability) of pilot trial methods and findings to future definitive trial and other studies  NP*: Generalizability (external validity) of the trial findings according to the intervention, comparators, patients, care providers, and centers involved in the trial | 36 |
| Interpretation | 22a | Interpretation consistent with pilot trial objectives and findings, balancing potential benefits and harms, and  considering other relevant evidence  NP*: In addition, take into account the choice of the comparator, lack of or partial blinding*, and unequal expertise of care providers or centers in each group | 31-37  31-37 |
|  | 22b | Implications for progression from pilot to future definitive trial, including any proposed amendments | 31-37 |
| **Other information** | | | |
| Registration | 23 | Registration number for pilot trial and name of trial registry | 9 |
| Protocol | 24 | Where the pilot trial protocol can be accessed, if available | 9 |
| Funding | 25 | Sources of funding and other support (such as supply of drugs), role of funders | N/A |
|  | 26 | Ethical approval or approval by research review committee, confirmed with reference number | 9 |

Eldridge et al. (2016) , Boutron et al. (2008)

*NP: Nonpharmacologic treatment extension guidance
