# Supplementary material for: A Neuroaffirmative, Self-Determination Theory–Based Psychosocial Intervention for Adults With Attention-Deficit/Hyperactivity Disorder: Randomized Feasibility Study
Source: JMIR Form Res. 2025 Oct 29;9:e69943. doi: 10.2196/69943 (PMC12612647; doi:10.2196/69943)
Supplement: Multimedia Appendix 2 [file formative_v9i1e69943_app2.docx]

# Supplement 2: The ADAPT Framework Practitioner Intervention Guide

# Introduction and Foundations

## Overview of the ADAPT Framework

The ADAPT Framework addresses maladaptive behaviors and functional impairments in ADHD by attributing them to psychopathology stemming from misunderstandings of atypical neurobiological development. This leads to needs neglect, thwarting, and lack of autonomy support for identity exploration and self-development.

- **Core Aims**: Provide autonomy-supported exploration of neurobiological, psychological, and individual needs; facilitate experimentation with identity construction and need-supportive strategy design across contexts.
- **Key Components**: The ADAPT Framework offers a neurobiological model for foundational understanding; supports psychological need satisfaction (autonomy, competence, relatedness) to identify authentic preferences and goals; supports development of client "authentic inner compass" (AIC) for self-direction, intrinsic motivation, and identity integration; and equips clients with a flexible "toolbox" of principles for contextual engagement.

## Foundations

ADHD presentations are multi-layered, involving behavioral self-regulation, comorbidities, mental health, trauma, career, self-esteem, identity, and relationships. Newly diagnosed individuals often grieve while integrating the diagnosis (Young et al., 2008). While lived experience of ADHD would be useful for practitioners, the framework is accessible via universal philosophical/theoretical perspectives in a variety of contexts.

- **Philosophical Perspectives**: Existential-phenomenological and humanistic, emphasizing human freedom, personal responsibility, individual-environment relationships, subjective experience, and meaning creation.
- **Self-Determination Theory (SDT) Perspective:** (Deci & Ryan, 1985; Ryan & Vansteenkiste, 2023) Organismic approach viewing humans as open systems sustaining via environmental exchanges. Assumes organizational (maintenance/extension) and developmental (growth) tendencies; and is person-centered, focusing on both an internal frame of reference alongside an external point of view (Ryan et al., 2021).
  - **Mini-Theories**:
    - *Cognitive Evaluation Theory (CET):* Intrinsic motivation energised via need satisfaction; influenced by informational, controlling, or amotivating evaluations; dual-process model highlights key elements of approach to motivating styles are separation and combined application of level of autonomy support and level of interpersonal control.
    - *Organismic Integration Theory (OIT):* Differentiates quantity of behavioural energy and quality of behavioural motivation; internalization of non-intrinsically motivated behaviours facilitated by need support.
    - *Causality Orientations Theory (COT):* Orientations along a continuum of engagement with environmental cues have individual differences (autonomy, controlled, impersonal); predicts motivational tendencies of environmental engagement.
    - *Goal Contents Theory (GCT):* Intrinsic goals (growth, health, relationships) enhance wellness; extrinsic goals impede it.
    - *Relationships Motivation Theory (RMT):* Mutual need fulfillment fosters optimal relationships and pro-relationship behaviours.
  - **Subjective Vitality**: Healthy functioning demonstrates behaviour characterised by awareness (clear perception) and excitement (energy mobilisation), providing evidence for internal positive energy for purposeful actions. Subjective vitality is an indicator of wellness which is enhanced by autonomy, intrinsic goals, need satisfaction and influenced by physiological factors (e.g., sleep, exercise).
  - **Integrated Emotion Regulation (IER)**: Emotion regulation is critical for ADHD emotional intensity and a key component in both SDT and the ADAPT Framework. IER identifies dual dimensions, non-judgmental attention and volitional exploration, as central to regulation. Styles include integrative (autonomous), suppressive (controlled), and dysregulated (nonvolitional) (Roth et al., 2019).

# Theoretical Perspectives and Psychopathology

## Gestalt Theory Perspective

Gestalt theory structure aligns with SDT and supports the ADAPT Framework aims through integration of consciousness theory, scientific theory, and a model for therapist-client relationship.

**Pillars:** Phenomenology (awareness in present), Field Theory (contextual influence), Dialogue (emergent contact).

- **Holism**: Views experience as organismic synthesis (body, feeling, intellect, imagination) linked to environment; perceives in patterned wholes, driving gestalt formation for sense-making.
- **Awareness, Contact, and Identity**: Centralizes awareness for change via Paradoxical Theory of Change (Beisser, 1970); contact as ongoing meaning-making; boundary functions connect/separate for needs and autonomy. Creative adjustments maintain equilibrium.
- **Field Perspective**: Dynamic interaction of context (Lewin, 1936); perception via contrast/figures-ground; contact-withdrawal rhythm and flow of experience measure health.
- **Creative Adjustment**: Reciprocal self-environment negotiation; differentiation through the polarity principle (Friedlander, 1918); and development of awareness skills: open/undirected (receptive, curious) and active/directed (analytical).

## Ericksonian Mindset

Influences practitioner perspective for therapeutic relationship in a nondirective, naturalistic style.

- **Key Concepts**: Individualized approach (tailored, responsive); flexible response (adapt to client); resourceful client (innate growth potential; focus on potential/resources/results over pathology/problems/reasons); utilization (integrate all experiences; methods: reframing, paradoxical interventions); altering patterns (shift inflexibility via small changes).

## Psychopathology

- **Need Frustration and Chronic Thwarting (SDT)**: Pervasive thwarting of basic psychological needs during development impairs autonomy and hinders curiosity, empathy, and executive functioning; leads to pressure/failure/isolation; fosters maladaptive need substitutes and comping mechanisms (Vansteenkiste & Ryan, 2013).
- **Resistance and Experiments (Gestalt)**: Resistance as contact moderation due to disrupted awareness/support; viewed as lack of integration ("knowing about" vs. owning one’s actions); addressed via experiments for data/insight, not control; promotes awareness in safe environment.

# Role of the Practitioner

The ADAPT Framework approach is humanistic and person-centered, drawing from SDT (autonomy-supportive, modeling authenticity), Gestalt (core attitude: actuality/awareness/responsibility; present-orientation, trusting process), mindfulness, and Ericksonian models.

**Focus:** Collaborative exploration; need support for internalization; curiosity in goal meanings. Flexible application: Therapeutic (identity/self-regulation) or coaching (goal-specific, bounded). Practitioners working in a coaching context should refer if therapeutic needs arise.

Framework Elements and Treatment Design

## ADAPT Framework Elements

- **Autonomy-Support**: Nurtures AIC via practices (perspective-taking, empathy, nurturing interests, choices, rationales); counters ADHD controlling history; includes inherent value demonstration (IVD) and reflective facilitation (Assor et al., 2023).
- **Design**: Supports self-development via:
  - Identity coherency: Neuroaffirmative neurobiological model for validation/exploration.
  - Authentic self-direction: Affect feedback, awareness for AIC strengthening.
  - Specific context orientation: Explore motivations in relation to context variability.
  - Life crafting: Proactive alignment of domains (job/home/leisure/need/emotion) to values/needs; Crafting efforts aligned with approach/avoidance motives (de Bloom et al., 2020).
  - Goal persistence: Autonomy-supported exploration; present-moment progress; co-design of experiments.
- **Awareness**: Promotes IER (curiosity/exploration) for information gathering; Establish integration preconditions: autonomy, open access, non-defensiveness; mindfulness for nonjudgmental attention which research shows enhances neurocognitive changes in ADHD (Cairncross & Miller, 2016).
- **Psychoeducation**: Clarifies neuroaffirmative ADHD neurobiology (Champ et al., 2022); introduces CAT (polar consciousness, self-regulation) for validation/pattern identification (Champ et al., 2024)(Figure 1).
- **Training**: Practitioners support skills training with scaffolding for independence (collaborative goals, zone of proximal development, gradual removal) through autonomy-supportive practice. Primary skills development in the intervention are: time management, organization (project/maintenance/space), planning (financial/dietary), academic (writing/studying/notes). Training sessions are framed with meaningful rationales.

**Figure 1:** Polar model of ADHD self-regulation


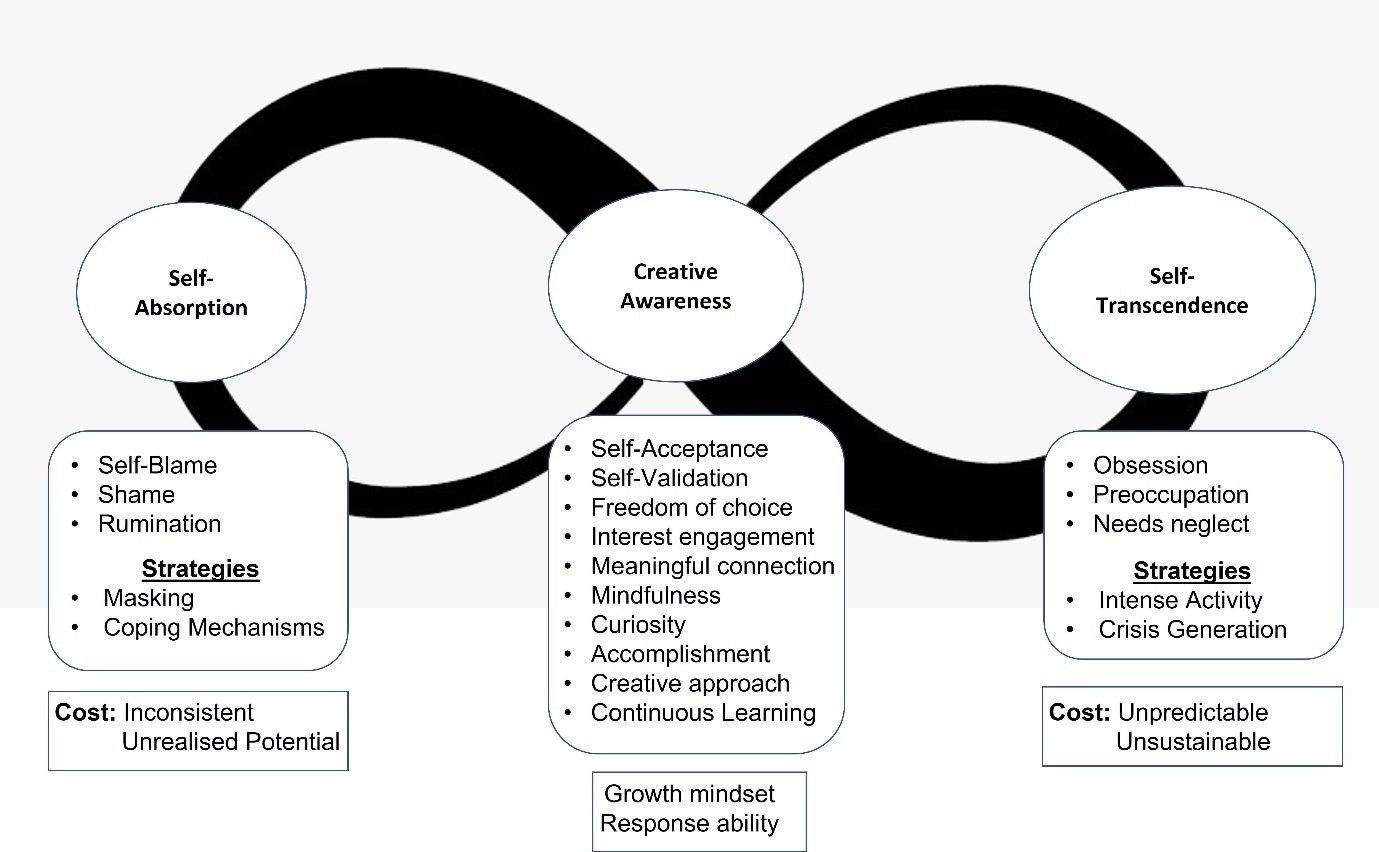


## Treatment Design

The ADAPT Farmwork prioritises a client-led structure. The recommended format is a 2-hour assessment + 10 weekly 60-minute sessions (final includes review).

- **Assessment Session**: Semi-structured review of client history encouraging sharing their story and challenges; exploration of lifestyle contexts (hyperactivity, impulsivity, risk comfort, distractibility, emotional dysregulation, family history, self-care, hobbies) with open-ended prompts for current relevant experiences. Practitioner introduces neuroaffirmative neurobiology model (last 30-45 min) and need-based outcomes are identified.
- **Treatment Sessions**: Sessions consist of primarily client-initiated discussions where practitioner guides the model application. Only Session 1 is structured, focusing on time management principles/tools (neuroaffirmative ADHD-specific). Subsequent sessions address client-led challenges and suggest awareness experiments (no mandatory homework). Sessions should aim to evolve to client agency.
- **Client Outcomes**: Need-based comparisons are used to frame client outcomes. Goal-oriented outcomes should arise autonomously from discussions.

## Conclusion

The ADAPT Framework empowers ADHD individuals to understand their neuroaffirmative neurobiology, leverage strengths, build their AIC, and develop Creative Awareness for self-regulation. Autonomy-supportive practitioner relationships foster need satisfaction, flexible strategies, and independent adaptation.

## References

Assor, A., Benita, M., & Geifman, Y. (2023). The authentic inner compass as an important motivational experience and structure: Antecedents and benefits. In R. M. Ryan (Ed.), *The Oxford Handbook of Self-Determination Theory* (pp. 363–386). Oxford University Press.

Beisser, A. R. (1970). The paradoxical theory of change. In J. Fagan & I. L. Shepherd (Eds.), *Gestalt Therapy Now* (pp. 77–80). Harper & Row.

Cairncross, M., & Miller, C. J. (2016). The effectiveness of mindfulness-based therapies for ADHD: A meta-analytic review. *Journal of Attention Disorders*, 108705471562530. https://doi.org/10.1177/1087054715625301

Champ, R. E., Adamou, M., Gillibrand, W., Arrey, S., & Tolchard, B. (2024). The creative awareness theory: A grounded theory study of inherent self-regulation in Attention Deficit Hyperactivity Disorder. *Journal of Clinical Medicine*, *13*, 5963. https://doi.org/https://doi.org/10.3390/jcm13195963

Champ, R. E., Adamou, M., & Tolchard, B. (2022). Seeking connection, autonomy, and emotional feedback: A self-determination theory of self-regulation in attention-deficit hyperactivity disorder. *Psychological Review*, 1–34. https://doi.org/10.1037/rev0000398

de Bloom, J., Vaziri, H., Tay, L., & Kujanpää, M. (2020). An identity-based integrative needs model of crafting: Crafting within and across life domains. *Journal of Applied Psychology*, *105*(12), 1423–1446. https://doi.org/10.1037/apl0000495

Deci, E. L., & Ryan, R. M. (1985). *Intrinsic motivation and self-determination in human behaviour*. Plenum Press.

Friedlander, S. (1918). *Schopferische Indifferenz*. Georg Muller.

Leary-Joyce, J. (2014). *The fertile void: Gestalt coaching at work*. Academy of Executive Coaching Press.

Lewin, K. (1936). *Principles of topological psychology*. McGraw-Hill.

Roth, G., Vansteenkiste, M., & Ryan, R. M. (2019). Integrative emotion regulation: Process and development from a self-determination theory perspective. *Development and Psychopathology*, 1–12. https://doi.org/10.1017/S0954579419000403

Ryan, R. M., Deci, E. L., Vansteenkiste, M., & Soenens, B. (2021). Building a science of motivated persons: Self-determination theory’s empirical approach to human experience and the regulation of behavior. *Motivation Science*, *7*(2), 97–110. https://doi.org/10.1037/mot0000194

Ryan, R. M., & Vansteenkiste, M. (2023). Self-Determination theory: Metatheory, methods and meaning. In R. M. Ryan (Ed.), *The Oxford handbook of self-determination theory* (pp. 1–30). Oxford University Press.

Vansteenkiste, M., & Ryan, R. M. (2013). On psychological growth and vulnerability: Basic psychological need satisfaction and need frustration as a unifying principle. *Journal of Psychotherapy Integration*, *23*(3), 263–280. https://doi.org/10.1037/a0032359

Young, S. J., Bramham, J., Gray, K., & Rose, E. (2008). A phenomenological analysis of the experience of receiving a diagnosis and treatment of ADHD in adulthood: a partner’s perspective. *Journal of Attention Disorders*, *12*(4), 299–307. https://doi.org/10.1177/1087054707311659
