# Supplementary material for: A Neuroaffirmative, Self-Determination Theory–Based Psychosocial Intervention for Adults With Attention-Deficit/Hyperactivity Disorder: Randomized Feasibility Study
Source: JMIR Form Res. 2025 Oct 29;9:e69943. doi: 10.2196/69943 (PMC12612647; doi:10.2196/69943)
Supplement: Multimedia Appendix 3 [file formative_v9i1e69943_app3.docx]

# Supplemental Material 2: Ethical approvals and participant forms

Contents

[SREIC Approval 2](#_Toc152679689)

[HRA and HRCW Approval 3](#_Toc152679690)

[Phase 2 Consent Form 4](#_Toc152679691)

[Phase 2 Patient information sheet 5](#_Toc152679692)

[Phase 2 Interview guide 9](#_Toc152679693)

[Phase 2 Invitation to interview 11](#_Toc152679694)

[Phase 3 Counselling agreement 12](#_Toc152679695)

[Phase 3 Information sheet 13](#_Toc152679696)

[Phase 3 Consent form 17](#_Toc152679697)

## SREIC Approval

Dear Rebecca,

**School Research Ethics and Integrity Committee (SREIC) Application**

**Rebecca Champ – PGR, School of Human and Health Sciences**

**Research Project Title:  Could a strength-based approach to treatment improve self-management in adults with Attention Deficit Hyperactivity Disorder?**

**SREIC Reference:  SREIC/2020/107**

The Panel Reviewers (Prof Ann Caress and Stephen Hogarth) have asked me to confirm that your ethics application as detailed above has now been **approved outright** and you may proceed with submitting your IRAS application.

This approval is subject to the content of the application in its current form.  If your research changes, from that in the application, it is incumbent on you as the researcher to update and seek further approval from SREIC/IRAS.  Without a re-approval your research is not supported by the University.

With best wishes for the success of your research project.

Regards,

Kirsty Thomson

(on behalf of the School Research Ethics and Integrity Committee (SREIC))

**School Research & Enterprise Office**


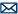
: [hhs_srep@hud.ac.uk](mailto:hhs_srep@hud.ac.uk)


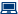
: [www.hud.ac.uk](https://eur02.safelinks.protection.outlook.com/?url=http%3A%2F%2Fwww.hud.ac.uk%2F&data=04%7C01%7CRebecca.Champ%40hud.ac.uk%7Cf8d35e86d7884e51db1f08d8e7bfb5b5%7Cb52e9fda06914585bdfc5ccae1ce1890%7C0%7C0%7C637514157861423087%7CUnknown%7CTWFpbGZsb3d8eyJWIjoiMC4wLjAwMDAiLCJQIjoiV2luMzIiLCJBTiI6Ik1haWwiLCJXVCI6Mn0%3D%7C1000&sdata=L7oTmFJ7%2BG5v%2Br6ePvbbHP14ogw6Ih0psoyVaJskgZo%3D&reserved=0)

School of Human and Health Sciences R&E Office
University of Huddersfield | Queensgate | Huddersfield | HD1 3DH

## HRA and HRCW Approval

## Phase 2 Consent Form


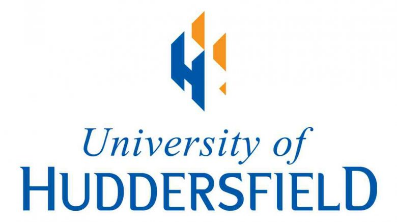


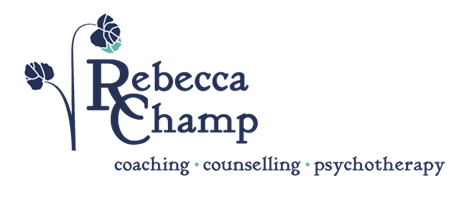


Phone: 07855 298536

Email: Rebecca.champ@hud.ac.uk

**General Consent and Right to Withdraw**

**Version 3 12/02/21 – IRAS Project ID: 291103**

**Title of Project: Could a strength- based treatment improve self-management in adults with Attention Deficit Hyperactivity Disorder?**

**Name of Researcher: Rebecca Champ**

**Please indicate that you agree to the following statements by putting your initials in the box(es) to the right:**

1. I confirm that I have read and understood the participant information sheet

dated ……………….(version 1, 2 etc.) for the above study and all my

questions have been answered satisfactorily.

1. I understand that my participation is voluntary and that I am free

to withdraw at any time before the study data is aggregated, without giving

any reason, and without my psychotherapeutic care or legal rights being affected.

1. I understand that anonymised sections of any of my psychotherapeutic

case notes may be looked at by individuals from the University of

Huddersfield, from regulatory authorities or from the NHS Trust, where it is

relevant to my taking part in this research.

1. I agree to take part in the above study.
2. I give permission to the researcher to contact my GP to inform them of my participation in this study.
3. I understand that my anonymised data may be used in an anonymous,

aggregated form to contribute to scientific articles, presentations and

publications.

……………………….. …………………….. …………………………..

Name Date Signature

……………………….. …………………….. …………………………

Name of person taking Date Signature

consent (if different from

researcher)

…………………………. ……………………. ………………………….

Researcher Date Signature

## Phase 2 Patient information sheet


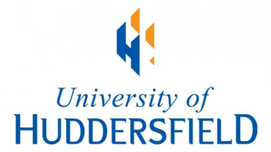


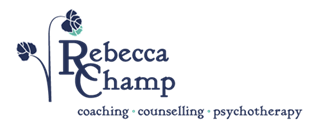


**Could a strength- based treatment improve self-management in adults with Attention Deficit Hyperactivity Disorder?**

*Information sheet for individuals with lived experience of ADHD*

*Version 4 22/05/21 – IRAS Project ID: 291103*

You are being invited to take part in a PhD Research Study. This leaflet explains why the research is being done and what taking part will involve. Please read the following information carefully and discuss it with others if you wish. You are welcome to talk to the researchers and ask questions before you decide.

After reading this leaflet, if you decide to take part please sign the consent form. It will not affect the standard of health care you receive if you decide not to take part.

**This information sheet is for you to keep.**

***Key Facts:***

- We are seeking to identify if there are natural strengths that come with ADHD
- We are interviewing people with ADHD to understand their experience of ADHD
- We hope to incorporate development of strengths into an ADHD treatment program
- We are seeking a wide range of experiences to get a good picture of living with ADHD
- Audio and video of interviews will be recorded, anonymised, and used for study purposes only
- Participation is voluntary and confidential, and you can withdraw before the data is aggregated
- You will receive the published results
- You will be entered into a draw for a £50 gift voucher

***What is the purpose of the study?***

Much of the current research on ADHD is focused on deficits and impairments of life with ADHD. This research explores the experiences of adults with ADHD who overcome daily challenges and find ways to be successful. It is hoped this project will highlight strengths of ADHD that can be supported and developed as skills in a therapeutic coaching model of support. This will provide an evidence base for effective therapy for adults with ADHD, increasing the options available to those looking for support separate from, or as an addition to, treatment with medication.

***What are the benefits and risks of taking part?***

By taking part in the research your experience of the symptoms of ADHD will be combined with other participants in order to develop a clearer picture of how those with ADHD overcome their impairments. This will help isolate common strengths and attributes that assist those with ADHD in being successful and will contribute to development of a treatment program to support and build on those strengths.

In addition, all interview participants will be entered into a draw for a £50 gift voucher.

The research only involves taking part in an interview. It will not involve taking any new medications or making any changes to your usual treatment. So we do not anticipate there being any risks to your health from taking part in this research. However, we recognise that the topic of the research may be sensitive for some individuals and our policy is not to send participants away from any research feeling distressed. If you feel upset by any issues being discussed, the researcher will provide support for you in the first instance and has received training in order to do this. We will also provide you with information, at the time of the interview, about where you can find support if you feel upset.

***Why have I been invited?***

I am keen to explore a wide variety of circumstances where opportunities have been available for those with ADHD to demonstrate their strengths. You have been offered a place in the study because you attend an organisation which supports people with ADHD. Your lived experience of ADHD presents a specific example of individual experience which contributes to a broad perspective for the research.

***Do I have to take part?***

Taking part in this research is entirely voluntary. If you do decide to take part you will be given this information sheet to keep and be asked to sign a consent form. You can stop being part of the study at any time, without giving a reason, but we will keep information about you that we already have. We need to manage your records in specific ways for the research to be reliable. This means that we won’t be able to let you see or change the data we hold about you. If you withdraw before the data has been anonymised and aggregated, all information about you will be destroyed. This will not affect the standard of care you receive.

***What will happen to me if I take part?***

Following a 30-minute introductory chat, you will be offered a 60-90 minute online interview with the researcher to explore your experience of living with ADHD. The researcher will ask you about what qualities help you to manage your ADHD successfully and explore their experience of these qualities. The interview will be conversational and free form to allow a picture of your lived experience of ADHD strengths and/or successes to develop from the interview. A guide of example questions is available if requested. Audio and video of the interview will be recorded and transcribed. The transcriptions will be anonymised, aggregated alongside others and analysed to extract the elements that pertain to the strengths that ADHD might help develop.

***How will we use information about you?***

We will need to use information from you for this research project. This information will include your name and contact details. People will use this information to do the research or to check your records to make sure that the research is being done properly. People who do not need to know who you are will not be able to see your name or contact details. Your data will have a code number instead. We will keep all information about you safe and secure. Once we have finished the study, we will keep some of the data so we can check the results. We will write our reports in a way that no-one can work out that you took part in the study.

All electronic documentation and recordings will be stored securely with password protection at the University of Huddersfield, and any paper documentation will be kept in a locked cabinet to which only the researcher will have access. All recorded research data information and consent forms will be archived for 10 years as per the University of Huddersfield Research Data Management Policy. There is potential for direct quotations from respondents to be published, but all will be anonymised.

***Where can you find out more about how your information is used?***

You can find out more about how we use your information:

- at www.hra.nhs.uk/information-about-patients/
- by asking one of the research team
- by sending an email to rebecca.champ@hud.ac.uk, or
- by ringing us on 07855 298536

***What if there is a problem?***

We will do our very best to ensure that no problems occur during your participation in this research. However, if you do have any concerns, the research team will be happy to discuss these with you – details for the lead researcher can be found at the end of this leaflet. If you have concerns or complaints arising from your experience of participating in this research that you do not wish to discuss with the research team directly, you can contact either the Associate Dean for Research in the School of Human and Health Sciences or, if your complaint relates to handling of your personal data, the University Solicitor at the University of Huddersfield or the Information Commissioner’s Office – details for all of these can be found at the end of this leaflet.

***What will happen to the results of the research study?***

The results will be submitted to the University of Huddersfield for assessment of the award of a Doctorate in Philosophy from the School of Human and Health Sciences. You will be provided with a copy of the published results as soon as they are available.

We plan to share the results of this research as widely as possible, as we very much want the experiences of people with ADHD to be heard. We will distribute summaries of the findings to ADHD charities/patient organisations, healthcare professionals, researchers, mass media (TV and newspaper) outlets and the general public. We will share the study’s results via our research team partners’ websites and social media outlets (e.g. Twitter, Facebook, LinkedIn). We will also communicate the study’s findings through papers in academic journals and presentations locally, nationally and internationally. We hope that the findings will be acted on by relevant parties (like policy makers and healthcare providers), but we can’t guarantee this.

***Who is organising and funding this research?***

The research is being led by researchers from the University of Huddersfield, which is also acting as the sponsor and data controller for the study (meaning that they are responsible for ensuring good conduct of the research and for looking after your data properly). The University of Huddersfield is responsible for overall management of this research and is providing insurance and indemnity. Use of ‘we’ throughout refers to the lead researcher and sponsor.

The research has not yet received any funding.

***Who has reviewed the study?***

The study has been approved by the University of Huddersfield School of Human and Health Sciences – School Research Ethics and Integrity Committee (SREIC) and by an NHS Research Ethics Committee (TBD).

***Contact for further information***

Thank you very much for your participation in this study. If you have any queries, please contact:

Rebecca Champ

Email: [Rebecca.champ@hud.ac.uk](mailto:Rebecca.champ@hud.ac.uk)

Mobile: 07855 298536

If you have a concern or complaint about the conduct of the research, please contact:

Prof Nick Hardiker, Associate Dean for Research, School of Human and Health Sciences, Room HW2/05 Harold Wilson Building, University of Huddersfield, Queensgate, HD1 3DH

Email: [n.hardiker@hud.ac.uk](mailto:n.hardiker@hud.ac.uk)

If your complaint relates to handling of your personal data, please contact

Rachel Main, University Solicitor, University of Huddersfield
E-mail: [Rachel.main@hud.ac.uk](mailto:Rachel.main@hud.ac.uk)

or

The Information Commissioner’s Office

See <https://ico.org.uk/make-a-complaint/your-personal-information-concerns/> or Ring 0303 123 1113.

***Thank you for taking time to read this information sheet and for considering taking part in our research***

## Phase 2 Interview guide

Initial Open-ended Questions:

1. How would you describe ADHD?
2. How do you think ADHD impacts those who have it?
3. What do you think people who have ADHD put in place to cope with it and reduce its impact on their lives?
4. How would you describe the relationship between those qualities and having ADHD?
5. How does having that experience feel?
6. Are there any experiences that you have with ADHD that you don’t hear other people with ADHD talking about much?

Intermediate Questions:

1. When did you first notice you had these experiences?
2. What was it like? If you recall, what were you feeling then?
3. Can you describe circumstances or events where you find you have these experiences?
4. If you recall, could you tell me about how you learned you could handle these experiences?
5. Are there tasks or circumstances that you feel suit you or you do well? Could you tell me about your thoughts and feelings when that is happening?
6. Could you describe a typical day for you when this is happening?
7. Are there tasks or situations where you notice or feel you are better equipped at handling than others around you?
8. Has anyone noticed when you are having this experience and communicated that to you? How do they describe these qualities?
9. What might others who know you say are your greatest strengths?
10. Do you know other people who have ADHD?
11. Do you know of anyone, personally or publicly, who is successful and has ADHD? Are there any positive qualities you notice in them that that might be familiar to yourself or others with ADHD?
12. What is it like to spend time with other people with ADHD?
13. Can you describe an environment (home, work or leisure) where you’ve felt that you were performing at your very best?

Ending Questions:

1. Could you tell me how your understanding has changed since you had a diagnosis?
2. What advice would you give someone who has just discovered they have ADHD?
3. Is there something that you might not have thought about before that occurred to you during this interview? Have you realised anything new about yourself or ADHD while talking to me today?
4. Has learning about ADHD, how it affects you, and how to work with instead of against it had an impact on your relationship with yourself? how you speak to and see yourself?
5. Is there something else you think I should know to understand your experience of ADHD better?
6. Is there anything you would like to ask me?

## Phase 2 Invitation to interview


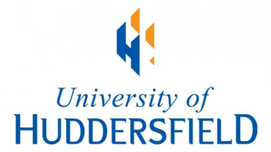


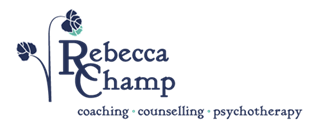


Phone: 07855 298536

Email: Rebecca.champ@hud.ac.uk

[DATE]

Dear .................

I am writing to invite you to participate in a research project as part of a PhD with the University of Huddersfield. You have been invited to participate because you attend an organisation which supports people with ADHD. I enclose a participant information sheet, which explains the title and aims of the project. Your unique experience could help us better understand ADHD life and this will help us develop our knowledge and support for people living with ADHD across the UK.

We are exploring circumstances where individuals with an ADHD diagnosis feel they were able to demonstrate and utilise their strengths. This will involve an in person online interview.

If you are willing to be interviewed, the interview would take between 60 and 90 minutes.

The aim is a relaxed and open conversation of your positive experiences and how you relate to your understanding of ADHD. We confirm that:

- Anything you say is confidential
- Audio and video will be recorded for study purposes only and destroyed afterwards
- Any notes made as a result of the interview will be destroyed afterwards
- Your personal data will be anonymised so you cannot be identified
- The online interview can be booked at a convenient time for you

In addition, all interview participants will be entered into a draw for a £50 gift voucher.

If you would like to be interviewed please contact the main researcher, Rebecca Champ, via email at [Rebecca.champ@hud.ac.uk](mailto:Rebecca.champ@hud.ac.uk) to arrange a 30 minute introductory chat. If you decide not to be involved, I would like to assure you that your care will not be affected in any way. If you would prefer not to be involved, there is no need to respond and we will not contact you again. I appreciate your time and attention to this exciting contribution to research and support for adults with ADHD, and look forward to sharing the results with you publicly.

Yours sincerely,


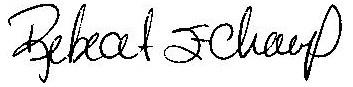


## Phase 3 Counselling agreement


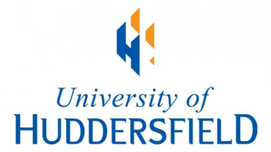


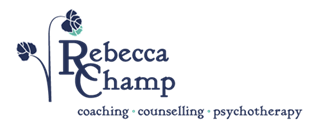


Phone: 07855 298536

Email: Rebecca.champ@hud.ac.uk

**Counselling Agreement**

| Name |  | Date of Birth |  |
| --- | --- | --- | --- |
| Address |  | | |
| Day/Evening Phone |  | Mobile Phone |  |
| Email |  | Skype Name |  |
| Name/Address of GP |  | | |
| Domestic situation | (co-habiting, single, moving etc.) | | |
| Medical History | (Undiagnosed physical symptoms, history of epilepsy, long term conditions, mental health issues etc.) | | |
| Any current medication/  Drug use |  | | |

**Our Confidentiality**

As a professional coach and therapist, I respect your confidentiality and adhere to a strict code of ethics. Unless required by law, I will not share details of our conversations with anyone in any way or divulge the nature of our relationship without your prior permission. I am collecting your phone number, email and personal health information for the purpose of maintaining communication, clear contracting, and reference notes on our work together. It may become necessary to share your data for the lawful reason of risk of harm to self or court order. I will only share your information with official legal bodies and /or relevant professionals under my Duty of Care. I will store your personal data in my secure locked storage and will store it for our agreed duration for the purpose of staying in contact during our work together. If we are no longer working together, I will dispose of your personal data as confidential waste immediately.

**Termination:** 24 hours notice by either party.

Your Appointments

**Scheduling Appointments:** Bookings set by agreed program. If required, confirmations and reminders of your appointments will be sent via email/text/telephone as agreed. You may cancel or reschedule your appointments with 24 hours notice.

**Calling in:** Please call me at the scheduled time on **07855 298536** or via the online video link provided. If you call in and get my voicemail, please call back after two full minutes. Please do not leave a message and wait for me to call you back.

**Cancellations:**  Cancellations without 24 hours notice are considered forfeiture of your scheduled therapy time and cannot be rescheduled. In case of an emergency, I will do my best to reschedule.

*____________________________________________________________________________*

*Client Signature Date*

## Phase 3 Information sheet


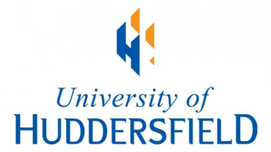

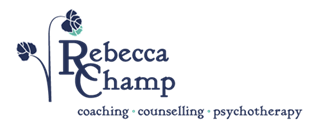


**Could a strength- based treatment improve self-management in adults with Attention Deficit Hyperactivity Disorder?**

*Information sheet for individuals with ADHD experiencing challenges in daily living*

*Version 4 22/05/21 – IRAS Project ID: 291103*

You are being invited to take part in a PhD Research Study. This leaflet explains why the research is being done and what taking part will involve. Please read the following information carefully and discuss it with others if you wish. You are welcome to talk to the researchers and ask questions before you decide.

After reading this leaflet, if you decide to take part please sign the consent form. It will not affect the standard of health care you receive if you decide not to take part.

**This information sheet is for you to keep.**

***Key Facts:***

- We are testing the feasibility, acceptability and efficacy of a new treatment approach for ADHD
- This approach is based on positive perspectives of ADHD as a difference instead of a deficit
- The program aims to develop strengths of ADHD and improve self-awareness
- There will be a 30-60 minute online assessment session before the treatment, and a 60 minute online exit interview after the treatment
- The program is delivered online, and consists of one 2-hour therapeutic coaching session and 10 1-hour weekly therapeutic coaching sessions over a period of 12 weeks
- Personal information of all participants will be anonymised, and used for study purposes only
- Participation is voluntary and confidential, and you can withdraw before the data is aggregated
- You will receive the published results

***What is the purpose of the study?***

Currently, those diagnosed with ADHD are offered treatment based on a perspective that sees ADHD only as a limiting and negative factor in people’s lives. This is based on a medical model that focuses on differences as malfunctions that need to be corrected to achieve normal and socially acceptable behaviour. Traditional treatment consists of medication and interventions designed to manage and reduce visible symptoms.

This research aims at offering a different perspective and approach to ADHD, one that stems from the understanding of the lived experience and resources of those with ADHD who have been able to harness what potential for development it has to offer.

***What is Therapeutic Coaching?***

This short-term therapeutic approach combines information to help understand how the ADHD brain is wired differently from others, therapy aimed at understanding the emotional experience of living with ADHD and its impact on daily life, and coaching based on strengths to develop specific skills tailored for individual goals. The focus is on helping you feel confident to make choices and take action toward what you want out of life. It does this though developing self-awareness, new skills and creating new ways of doing things alongside a coach.

***Why have I been invited?***

You have been offered a place on the study because you have a diagnosis of ADHD and staff at the Adult ADHD Clinic at the Southwest Yorkshire Partnership NHS Foundation Trust have identified you as a potential participant. Research shows individuals with ADHD experience challenges with symptoms that negatively impact daily functioning, quality of life, self-esteem and ability to achieve success.

***Do I have to take part?***

Taking part in this research is entirely voluntary. If you do decide to take part, you will be given this information sheet to keep and be asked to sign a consent form You can stop being part of the study at any time, without giving a reason, but we will keep information about you that we already have. We need to manage your records in specific ways for the research to be reliable. This means that we won’t be able to let you see or change the data we hold about you. If you withdraw before the data has been anonymised and aggregated, all information about you will be destroyed. This will not affect the standard of care you receive.

***What will I be asked to do?***

Following an introductory 30–60-minute online assessment, you will be offered a 120-minute (2 hour) online impact-assessment session. This will be followed by 10 individual 60 minute online therapeutic coaching sessions at weekly intervals, plus an ending session interview. During these sessions your experience of the symptoms of ADHD will be explored and treated using the multi-modal approach. All sessions will be recorded and transcribed.

Both your GP and the South West Yorkshire Partnership NHS Foundation Trust Adult ADHD Clinic will be informed of your participation. Should any issues arise as a result of participation in the study, additional support will be requested.

The study aims to compare how you feel before and after treatment and before each session. We will do that using several research methods which are detailed below this list:

- The Weiss Functional Impairment Rating Scale Self-Report (WFIRS-S)
- The Patient Health Questionnaire (PHQ-9)
- The Generalised Anxiety Disorder Questionnaire (GAD-7)
- Harter’s Self Perception Profile
- The Index of Autonomous Functioning (IAF)
- The Perceived Choice and Awareness Scale (PCASS)
- The Personal Questionnaire forms

***Ending Interview***

One week after the last session we will complete the study by conducting an Ending Interview. The focus of this 60-minute session will be to fill out forms to measure any difference between before treatment started and after treatment.

***How much time will I spend in study activities?***

**Overview of Pilot Study Assessment Time and Frequency**

**
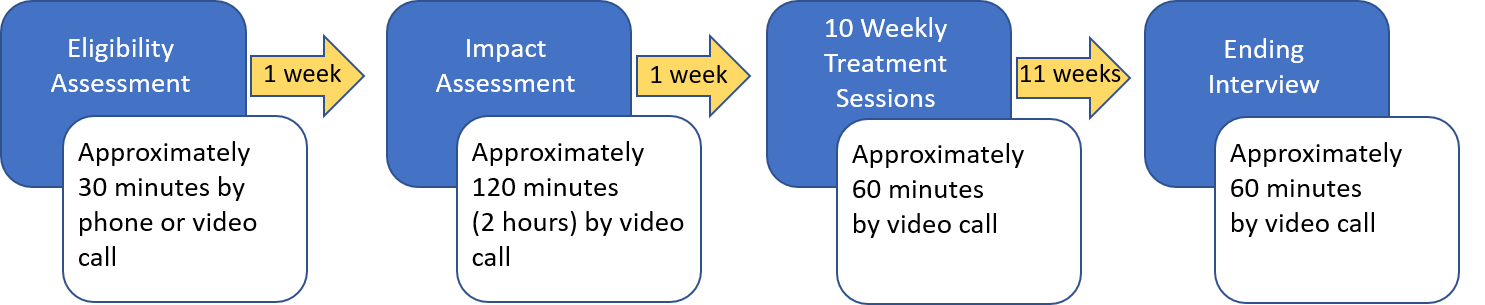
**

***How will we use information about you?***

We will need to use information from you for this research project. This information will include your name and contact details. People will use this information to do the research or to check your records to make sure that the research is being done properly. People who do not need to know who you are will not be able to see your name or contact details. Your data will have a code number instead. We will keep all information about you safe and secure. Once we have finished the study, we will keep some of the data so we can check the results. We will write our reports in a way that no-one can work out that you took part in the study.

All electronic documentation and recordings will be stored securely with password protection at the University of Huddersfield, and any paper documentation will be kept in a locked cabinet to which only the researcher will have access. The sessions will be recorded for random selection review by university supervisors to ensure fidelity of the intervention, which will be destroyed on review completion. All recorded research data information and consent forms will be archived for 10 years as per the University of Huddersfield Research Data Management Policy. There is potential for direct quotations from respondents to be published, but all will be anonymised.

***Where can you find out more about how your information is used?***

You can find out more about how we use your information:

- at www.hra.nhs.uk/information-about-patients/
- by asking one of the research team
- by sending an email to rebecca.champ@hud.ac.uk, or
- by ringing us on 07855 298536

***What are the possible benefits and risks of taking part?***

By taking part in the research, you will receive a psychotherapeutic treatment specifically designed to address challenges experienced by individuals with an ADHD diagnosis. Your results will be combined with other participants in order to examine the feasibility, acceptability and efficacy of the treatment approach for individuals with ADHD.

The research only involves taking part in therapeutic coaching sessions. It will not involve taking any new medications or making any changes to your usual treatment. So we do not anticipate there being any risks to your health from taking part in this research. However, we recognise that the topic of the research may be sensitive for some individuals and our policy is not to send participants away from any research feeling distressed. If you feel upset by any issues being discussed, the researcher will provide support for you in the first instance and has received training in order to do this. We will also provide you with information, at the time of the interview, about where you can find support if you feel upset.

***What if there is a problem?***

We will do our very best to ensure that no problems occur during your participation in this research. However, if you do have any concerns, the research team will be happy to discuss these with you – details for the lead researcher can be found at the end of this leaflet. If you have concerns or complaints arising from your experience of participating in this research that you do not wish to discuss with the research team directly, you can contact either the Associate Dean for Research in the School of Human and Health Sciences or, if your complaint relates to handling of your personal data, the University Solicitor at the University of Huddersfield or the Information Commissioner’s Office – details for all of these can be found at the end of this leaflet.

***What will happen to the results of the research study?***

The results will be submitted to the University of Huddersfield for assessment of the award of a Doctorate in Philosophy from the School of Human and Health Sciences. You will be provided with a copy of the published results as soon as they are available.

We plan to share the results of this research as widely as possible, as we very much want the experiences of people with ADHD to be heard. We will distribute summaries of the findings to ADHD charities/patient organisations, healthcare professionals, researchers, mass media (TV and newspaper) outlets and the general public. We will share the study’s results via our research team partners’ websites and social media outlets (e.g. Twitter, Facebook, LinkedIn). We will also communicate the study’s findings through papers in academic journals and presentations locally, nationally and internationally. We hope that the findings will be acted on by relevant parties (like policy makers and healthcare providers), but we can’t guarantee this.

***Who is organising and funding this research?***

The research is being led by researchers from the University of Huddersfield, which is also acting as the sponsor and data controller for the study (meaning that they are responsible for ensuring good conduct of the research and for looking after your data properly). The University of Huddersfield is responsible for overall management of this research and is providing insurance and indemnity. Use of ‘we’ throughout refers to the lead researcher and sponsor.

The research has not yet received any funding.

***Who has reviewed the study?***

The study has been approved by the University of Huddersfield School of Human and Health Sciences – School Research Ethics and Integrity Committee (SREIC) and by an NHS Research Ethics Committee (TBD).

***Contact for further information***

Thank you very much for your participation in this study. If you have any queries, please contact:

Rebecca Champ

Email: [Rebecca.champ@hud.ac.uk](mailto:Rebecca.champ@hud.ac.uk)

Mobile: 07855 298536

If you have a concern or complaint about the conduct of the research, please contact:

Prof Nick Hardiker, Associate Dean for Research, School of Human and Health Sciences, Room HW2/05 Harold Wilson Building, University of Huddersfield, Queensgate, HD1 3DH

Email: [n.hardiker@hud.ac.uk](mailto:n.hardiker@hud.ac.uk)

If your complaint relates to handling of your personal data, please contact

Rachel Main, University Solicitor, University of Huddersfield
E-mail: [Rachel.main@hud.ac.uk](mailto:Rachel.main@hud.ac.uk)

or

The Information Commissioner’s Office

See <https://ico.org.uk/make-a-complaint/your-personal-information-concerns/>

***Thank you for taking time to read this information sheet and for considering taking part in our research***

## Phase 3 Consent form


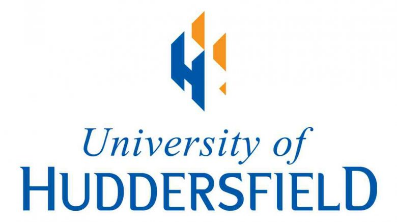


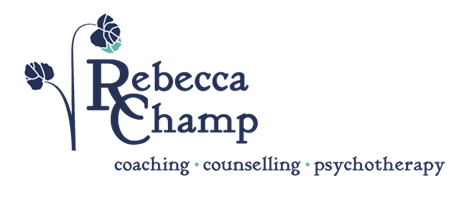


Phone: 07855 298536

Email: Rebecca.champ@hud.ac.uk

**General Consent and Right to Withdraw**

**Version 3 12/02/21 – IRAS Project ID: 291103**

**Title of Project: Could a strength- based treatment improve self-management in adults with Attention Deficit Hyperactivity Disorder?**

**Name of Researcher: Rebecca Champ**

**Please indicate that you agree to the following statements by putting your initials in the box(es) to the right:**

1. I confirm that I have read and understood the participant information sheet

dated ……………….(version 1, 2 etc.) for the above study and all my

questions have been answered satisfactorily.

1. I understand that my participation is voluntary and that I am free

to withdraw at any time before the study data is aggregated, without giving

any reason, and without my psychotherapeutic care or legal rights being affected.

1. I understand that anonymised sections of any of my psychotherapeutic

case notes may be looked at by individuals from the University of

Huddersfield, from regulatory authorities or from the NHS Trust, where it is

relevant to my taking part in this research.

1. I agree to take part in the above study.
2. I give permission to the researcher to contact my GP to inform them of my participation in this study.
3. I understand that my anonymised data may be used in an anonymous,

aggregated form to contribute to scientific articles, presentations and

publications.

……………………….. …………………….. …………………………..

Name Date Signature

……………………….. …………………….. …………………………

Name of person taking Date Signature

consent (if different from

researcher)

…………………………. ……………………. ………………………….

Researcher Date Signature
