# Supplementary material for: A Neuroaffirmative, Self-Determination Theory–Based Psychosocial Intervention for Adults With Attention-Deficit/Hyperactivity Disorder: Randomized Feasibility Study
Source: JMIR Form Res. 2025 Oct 29;9:e69943. doi: 10.2196/69943 (PMC12612647; doi:10.2196/69943)
Supplement: Multimedia Appendix 4 [file formative_v9i1e69943_app4.docx]

# **Supplemental Material 4: Statistical analysis report**

## Sample

The pilot study comprised 23 informed and consenting NHS adult patients, with a confirmed diagnosis of ADHD, and no known mental health comorbidity. Their age varied between 20 and 56 years-old, but the oldest person was identified as an outlier. This was the participant with an ID=6. Then, most participants were young adults. Their mean was 33.35 years (SD=10.1), and was not normally distributed (W(23)=0.889; p=0.015).


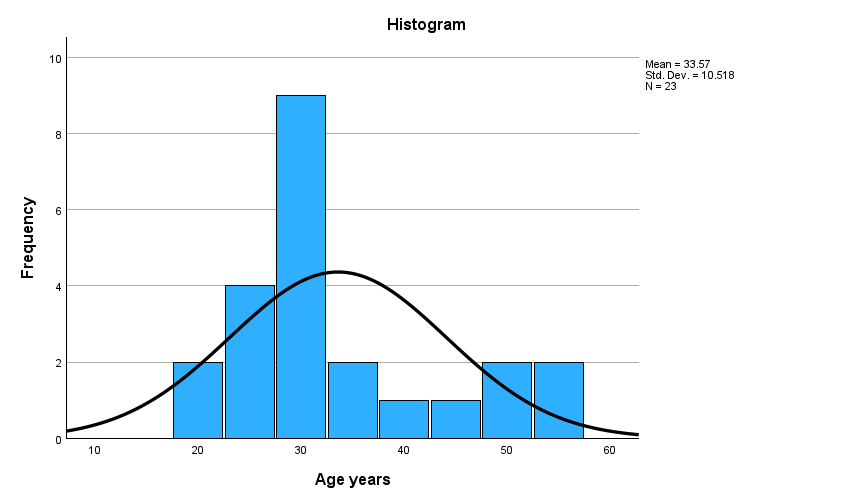


**Figure 1:** Distribution of age

In terms of gender, as self-ascribed, there were 13 males (56.53%), eight females (34.78%), and two transgender (8.7%). Most participants, namely 20 (87%), further self-identified as White British citizens. The remainder identified as “Portuguese”, ID=21, which is a nationality, one identified as “White South African”, ID=, and another as White American, ID=7. These answers represent a mixture of race and nationality. Therefore, a not ‘White and British’ category was created to group these participants in the same race/ethnicity group.

Regarding their clinical characteristics, seven (30.43%) participants stated that their diagnosis was inattentive, two (8.7%) that it was hyperactive, and 14 (60.9%), the majority, presented a mixture of hyperactivity and inattention symptoms, or “combined” in the present study. The date when participants had received their clinical diagnosis varied between 1999 and 2022. Figure 2, below, illustrates the distribution of dates. It was apparently not normally distributed (W(23)=0.734; p≤0.001). A closer inspection further identifies the participant with an ID=11, with the oldest diagnosis, as an outlier.


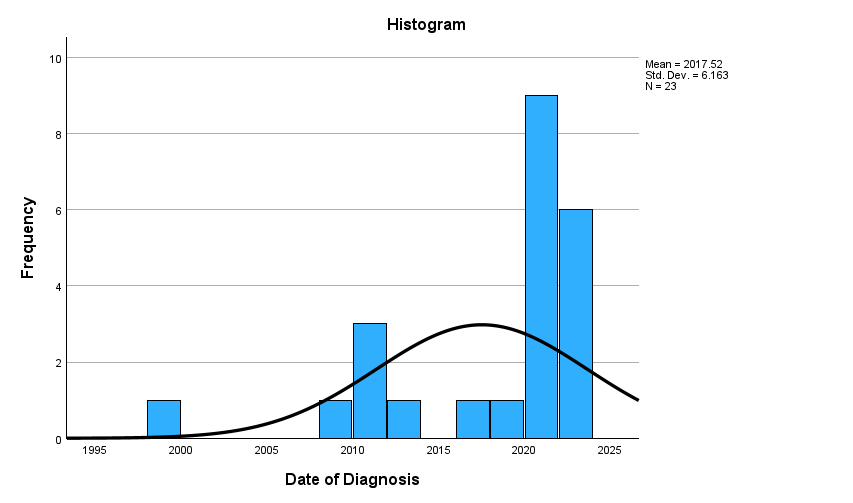


**Figure 2:** Distribution of the frequency of the date of diagnosis

Additionally, most diagnoses were received after 2020. For this reason, as unpacked in the Descriptive Statistics section, participants were also categorized into two groups: diagnosed before and diagnosed after 2020. There were 15 participants diagnosed after 2020, and almost half in the very long period that preceded this date.

Regarding medication, there were only five participants who were not medicated. Therefore, the majority, namely 18 (78.26%), were medicated. Finally, the months elapsed between pre-and post-test were, in average, 4.94 (SD=0.9) months. It did not have a normal distribution (SW(20)=0.31; p≤0.001).

The Descriptive Statistics section details how demographic variables were operationalized in the present study. Tables 3 and 4 offer more detailed demographic information for the whole sample than that which is presented in this section. The following section describes the groups under comparison in some detail.

## Data collection procedures

Between June and September of 2022, pretest assessments took place. This is the baseline or pretest stage. In this stage, Control and Intervention groups completed the following measures: Demographics; EQ-5D-5L; CORE-OM; ADHD-RS-Inv; AAQoL; SRI; and IAF.

Intervention 1 started immediately after pretest assessments for the Intervention group. It elapsed between May and December of 2022, depending on the date of recruitment. Where relevant, it involved the completion of ten PQ assessments, referred to as “T1”, “T2”, and so forth until “T10”, for up to a maximum of ten problems. This intervention lasted for three months. On the other hand, the Control group waited three months after the pretest stage. Therefore, in both groups, three months had elapsed after the pretest when the post-test was expected to start. At post-test, the same measures (Demographics; EQ-5D-5L; CORE-OM; ADHD-RS-Inv; AAQoL; SRI; and IAF) were used.

Post-test was expected to happen exactly three months after the pretest stage in both groups, whether or not they had been subjected to the Intervention. This did not always happen. As illustrated in Figure 3 below, there was apparently greater dispersion across the post-test data assessment dates of the Intervention group, compared to the Control group. Yet, there was not a single outlier or post-test never happened more than six months after the pretest.

**Figure 3:** Dispersion of data collection dates, per group


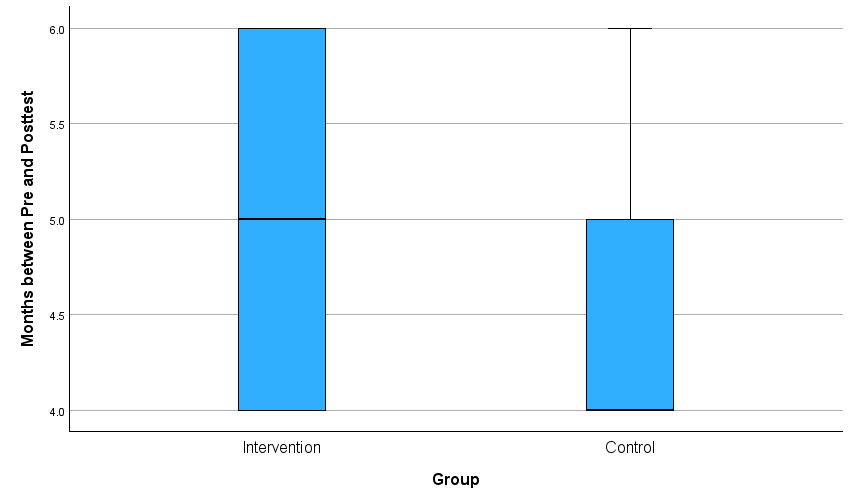


Post-test also involved completing the ten PQ assessment moments, for a maximum of ten problems. Since this survey was a part of the Intervention procedures, it cannot be used to compare both groups. Instead, it can only be used to compare the efficacy of the intervention in two different groups of participants. It was then to serve as a means of replication of the impact of the intervention, as evaluated specifically by PQ. A summary of these data collection steps is illustrated in the following Figure 4.

**Figure 4:** Data Collection Timeline


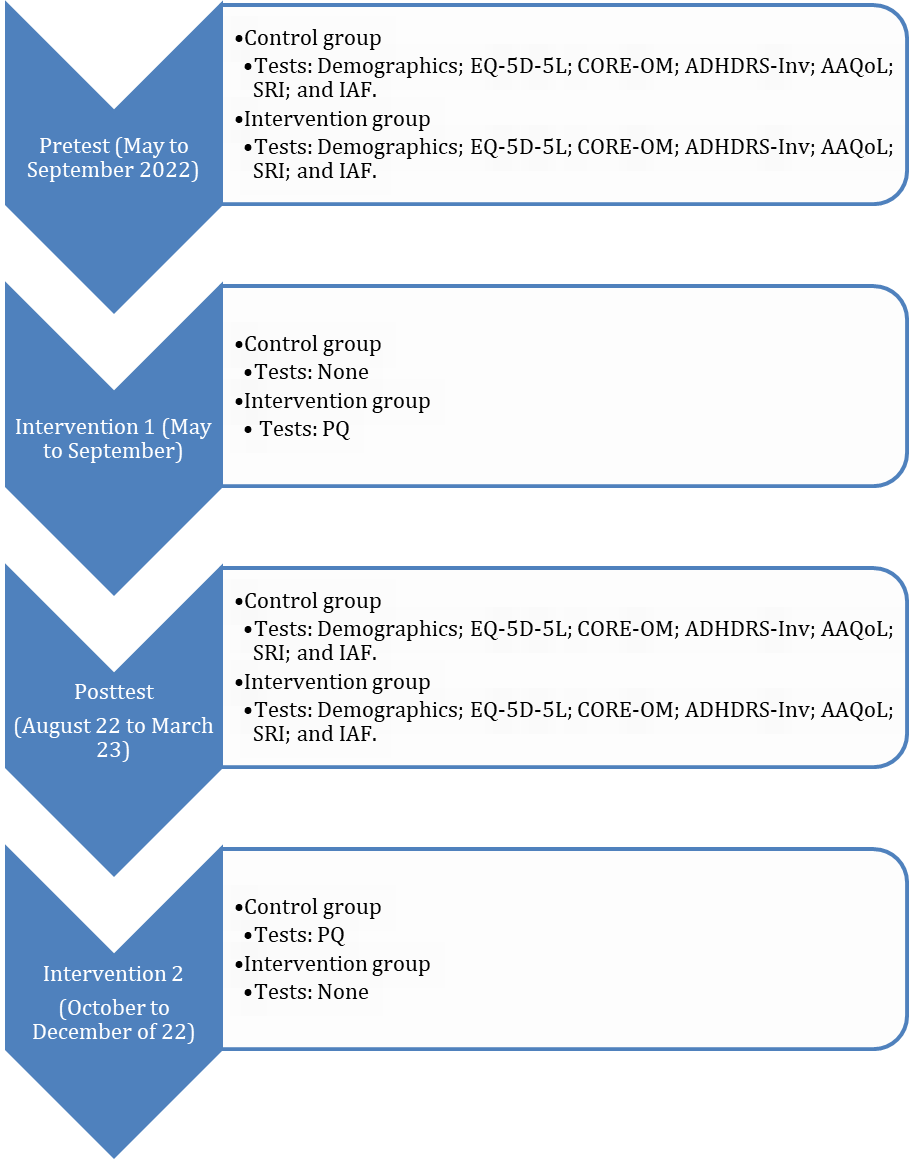


# List of variables and their SPSS classification and coding

**Table 1.** List of variables and their SPSS classification and coding

| N. | Variable Name | Measure/ Scale (Total items) | Subscale (Total items) | Variable Type | Operationalization |
| --- | --- | --- | --- | --- | --- |
| 1 | Group | Demographics (1) | N/A^1^ | Nominal | 1= ‘Intervention’; and  2= ‘Control’ |
| 2 | Dropout participants | Demographics (1) | N/A^1^ | Nominal | 1= ‘Participant’  2=’Dropout’ |
| 3 | Gender | Demographics (1) | N/A^1^ | Nominal | 1=‘Male’;  2=‘Female’; and  3=’Transgender’ |
| 4 | Age | Demographics (1) | N/A^1^ | Scale, where higher numbers indicate older ages | Number of Years of Life |
| 5 | Age Group | Demographics (1) | N/A^1^ | Ordinal, where lower values indicate younger persons | 1= ‘33 years-old or less’  2= ‘more than 33 years-old’ |
| 6 | Race-Nationality | Demographics (1) | N/A^1^ | Nominal | 1= ‘White British’;  2= ‘White South African’; and  3= ‘Portuguese’. |
| 7 | Race-Nationality groups | Demographics (1) | N/A^1^ | Nominal | 1= ‘White British’; and  2= ‘Not White, or not British’. |
| 8 | Diagnosis | Demographics (1) | N/A^1^ | Nominal | 1=’Inattentive’;  2=’Hyperactive’; and  3=’Combined’. |
| 9 | Date of Diagnosis | Demographics (1) | N/A^1^ | Discrete quantitative | Year when the diagnosis was made. |
| 10 | Date of Diagnosis group | Demographics (1) | N/A^1^ | Ordinal, where greater ratings indicate more recent diagnosis. | 1= ‘Before 2020’; and  2= ’During or after 2020’. |
| 11 | Medication | Demographics (1) | N/A^1^ | Nominal | 0=’None’; and  1=’Prescribed’. |
| 12 | Months between Assessments | Research Logs (1) | N/A^1^ | Discrete quantitative | Number of months between pretest and post-test |
| 13 | Mobility | EQ-5D-5L^3^ (5) | Mobility (1) | Ordinal, where high ratings indicate poorer health states | Ratings on the scale below.  1= ‘No problems’;  2= ‘Slight problems’;  3= ‘Moderate problems’;  4= ‘Severe problems’; and  5= ‘Extreme problems’. |
| 14 | Self-Care | EQ-5D-5L^3^ (5) | Self-Care (1) | Ordinal, where high ratings indicate poorer health states | Ratings on the scale below.  1= ‘No problems’;  2= ‘Slight problems’;  3= ‘Moderate problems’;  4= ‘Severe problems’; and  5= ‘Extreme problems’. |
| 15 | Activities | EQ-5D-5L^3^ (5) | Activities (1) | Ordinal^2^ , where high ratings indicate poorer health states | Ratings on the scale below.  1= ‘No problems’;  2= ‘Slight problems’;  3= ‘Moderate problems’;  4= ‘Severe problems’; and  5= ‘Extreme problems’. |
| 16 | Pain | EQ-5D-5L^3^ (5) | Pain/Discomfort (1) | Ordinal, where high ratings indicate poorer health states | Ratings on the scale below.  1= ‘No problems’;  2= ‘Slight problems’;  3= ‘Moderate problems’;  4= ‘Severe problems’; and  5= ‘Extreme problems’. |
| 17 | Anxiety | EQ-5D-5L^3^ (5) | Anxiety/Depression (1) | Ordinal, where high ratings indicate poorer health states | Ratings on the scale below.  1= ‘No problems’;  2= ‘Slight problems’;  3= ‘Moderate problems’;  4= ‘Severe problems’; and  5= ‘Extreme problems’. |
| 18 | EQ5D5L Total | EQ-5D-5L^3^ (5) | EQ5D5L Total (5) | Discrete quantitative, where high ratings indicate poorer health states | Sum the ratings given to every scale item, namely 1 to 5, using the scale below.  1= ‘No problems’;  2= ‘Slight problems’;  3= ‘Moderate problems’;  4= ‘Severe problems’; and  5= ‘Extreme problems’. |
| 19 | Profile | EQ-5D-5L^3^ (5) | EQ-5D-5L^3^ (5) | Nominal | Aggregate the ratings given to every scale item, in the order of the presentation of the survey (Mobility; Self-Care;  Activities; Pain/ Discomfort; and  Anxiety/Depression), as a single five digit number, varying from 11111 to 55555 in a nominal scale, where 11111 indicates good health and 55555 poor health in every domain. |
| 20 | Index Value | EQ-5D-5L^3^ (5) | EQ-5D-5L^3^ Index (5) | Discrete quantitative, where high ratings indicate poorer health states | Transform the profile of each participant into an index value, with the help of a nation-by-nation standardized dataset, through the procedures specified in Table 2. |
| 21 | Productivity | AAQoL^4^ (29) | Life Productivity (11) | Discrete quantitative, where higher scores indicate poorer quality of life | 1) Convert the ratings into a 0-100 scale (1=0; 2=25; 3=50; 4=75; 5=100);  2) Sum the ratings for the items 1r^2^, 2r^2^, 3r^2^, 4r^2^, 5r^2^, 6r^2^, 7r^2^, 8r^2^, 9r^2^, 10r^2^, and 11r^2^; using the scale below; and    3) Divide the total by the total number of items in the scale, 11.  1=’Never’:  2=’Almost never’;  3=’Sometimes’;  4=’Fairly often’; and  5=’Very often’. |
| 22 | Mental health | AAQoL^4^ (29) | Mental Health/ Psychological Health (6) | Discrete quantitative, where higher scores indicate poorer quality of life | 1) Convert the ratings into a 0-100 scale (1=0; 2=25; 3=50; 4=75; 5=100);  2) Sum the ratings for items 12r^2^, 13r^2^, 14r^2^, 15r^2^, 16r^2^, and 17r^2^, using the scale below; and  3) Divide by the total number of items in the scale, 6.  1=’Never’:  2=’Almost never’;  3=’Sometimes’;  4=’Fairly often’; and  5=’Very often’. |
| 23 | Outlook | AAQoL^4^ (29) | Life Outlook (7) | Discrete quantitative, where higher scores indicate poorer quality of life | 1) Convert the ratings into a 0-100 scale (1=0; 2=25; 3=50; 4=75; 5=100);  2) Sum the ratings for items 18, 19, 20, 21, 22, 23, and 24, using the scale below; and  3) Divide by the total number of items in the scale, 7.  1=’Never’:  2=’Almost never’;  3=’Sometimes’;  4=’Fairly often’; and  5=’Very often’. |
| 24 | Relationships | AAQoL^4^ (29) | Relationships (5) | Discrete quantitative, where higher scores indicate poorer quality of life | 1) Convert the ratings into a 0-100 scale (1=0; 2=25; 3=50; 4=75; 5=100);  2) Sum the ratings for items 25r^2^, 26r^2^, 27r^2^, 28r^2^, and 29r^2^, using the scale below; and  3) Divide by the total number of items in the scale, 5.  1=’Never’:  2=’Almost never’;  3=’Sometimes’;  4=’Fairly often’; and  5=’Very often’. |
| 25 | AAQoL Total | AAQoL^4^ (29) | Total (29) | Discrete quantitative, where higher scores indicate poorer quality of life | 1) Convert the ratings into a 0-100 scale (1=0; 2=25; 3=50; 4=75; 5=100);  2) Sum the ratings for every scale item, namely, 1r^2^, 2r^2^, 3r^2^, 4r^2^, 5r^2^, 6r^2^, 7r^2^, 8r^2^, 9r^2^, 10r^2^, 11r^2^,12r^2^, 13r^2^, 14r^2^, 15r^2^, 16r^2^, 17r^2^, 18, 19, 20, 21, 22, 23, 24, 25r^2^, 26r^2^, 27r^2^, 28r^2^, and 29r^2^, using the scale below, and  3) Divide by the total number of items in the scale, 29.  1=’Never’:  2=’Almost never’;  3=’Sometimes’;  4=’Fairly often’; and  5=’Very often’. |
| 26 | Wellbeing | CORE-OM^5^ (34) | Subjective well-being deficits (4) | Discrete quantitative, where higher scores indicate greater distress | 1) Sum the ratings for items 4r^2^, 14, 17, and 31r^2^, using the scale below; and  2) Divide by the total number of items in scale, 4.  0= ‘Not at all’;  1=‘ Only occasionally’;  2= ‘Sometimes’;  3= ‘Often’; and  4= ‘Most or all the time’. |
| 27 | Problems | CORE-OM^5^ (34) | Problems/Symptoms (12) | Discrete quantitative, where higher scores indicate greater distress | 1) Sum the ratings for items 2, 5, 8, 11, 13, 15, 18, 20, 23, 27, 28, and 30, using the scale below; and  2) Divide by the total number of items in scale, 12.  0= ‘Not at all’;  1=‘ Only occasionally’;  2= ‘Sometimes’;  3= ‘Often’; and  4= ‘Most or all the time’. |
| 28 | Functional issues | CORE-OM^5^ (34) | Life functioning déficts (12) | Discrete quantitative, where higher scores indicate greater distress | 1) Sum the ratings for items 1, 3r^2^, 7r^2^, 10, 12r^2^, 19r^2^, 21r^2^, 25, 26, 29, 32r^2^, and 33, using the scale below; and  2) Divide by the total number of items in scale, 12.  0= ‘Not at all’;  1=‘ Only occasionally’;  2= ‘Sometimes’;  3= ‘Often’; and  4= ‘Most or all the time’. |
| 29 | Risk | CORE-OM^5^ (34) | Risk/harm (6) | Discrete quantitative, where higher scores indicate greater distress | 1) Sum the ratings for items 6, 9, 16, 22, 24, and 34, using the scale below; and  2) Divide by the total number of items in scale, 6, using the scale below.  0= ‘Not at all’;  1=‘ Only occasionally’;  2= ‘Sometimes’;  3= ‘Often’; and  4= ‘Most or all the time’. |
| 30 | CORE-OM Total | CORE-OM^5^ (34) | CORE-OM^4^ (34) | Discrete quantitative, where higher scores indicate greater distress | 1) Sum every scale rating, namely, for items 1, 2, 3r^2^, 4r^2^, 5, 6, 7r^2^, 8, 9, 10, 11, 12r^2^, 13, 14, 15, 16, 17, 18, 19r^2^, 20, 21r^2^, 22, 23, 24, 25, 26, 27, 28, 29, 30, 31r^2^, 32r^2^, 33, and 34, using the scale below; and  2) Divide by the total number of scale items, namely, 34.  0= ‘Not at all’;  1=‘ Only occasionally’;  2= ‘Sometimes’;  3= ‘Often’; and  4= ‘Most or all the time’. |
| 31 | CORE-OM Non-Risk Total | CORE-OM^5^ (34) | Non-Risk (28) | Discrete quantitative, where higher scores indicate greater distress | 1) Sum the ratings for items 1, 2, 3r^2^, 4r^2^, 5, 7r^2^, 8, 10, 11, 12r^2^, 13, 14, 15, 17, 18, 19r^2^, 20, 21r^2^, 23, 25, 26, 27, 28, 29, 30, 31r^2^, 32r^2^, and 33, using the scale below; and  2) Divide by the total number of items, namely 28.  0= ‘Not at all’;  1=‘ Only occasionally’;  2= ‘Sometimes’;  3= ‘Often’; and  4= ‘Most or all the time’. |
| 32 | CORE-OM  Risk to Self | CORE-OM^5^ (34) | Risk to Self (4) | Discrete quantitative, where higher scores indicate greater distress | 1) Sum the ratings for items 9, 16, 24, and 34, using the scale below; and  2) Divide by the total number of items in scale, 4.  0= ‘Not at all’;  1=‘ Only occasionally’;  2= ‘Sometimes’;  3= ‘Often’; and  4= ‘Most or all the time’. |
| 33 | CORE-OM  Risk to Others | CORE-OM^5^ (34) | Risk to Others (2) | Discrete quantitative, where higher scores indicate greater distress | 1) Sum the ratings for items 6, and 22, using the scale below; and  2) Divide by the total number of items, namely, 2.  0= ‘Not at all’;  1=‘ Only occasionally’;  2= ‘Sometimes’;  3= ‘Often’; and  4= ‘Most or all the time’. |
| 34 | Authorship | IAF^6^ (15) | Authorship (5) | Discrete quantitative, where higher scores indicate greater autonomous authorship and self-congruence. | 1) Sum the ratings for items 1, 4, 8, 10, and 15, using the scale below.  1= ‘Not at all true’;  2= ‘A bit true’;  3= ‘Somewhat true’;  4= ‘Mostly true’; and  5= ‘Completely true’. |
| 35 | Control | IAF^6^ (15) | Control (5) | Discrete quantitative, where higher scores indicate greater autonomous lack of susceptibility to control. | 1) Sum the ratings for items 2r^2^, 6r^2^, 7r^2^, 11r^2^, and 14r^2^, using the scale below.  1= ‘Not at all true’;  2= ‘A bit true’;  3= ‘Somewhat true’;  4= ‘Mostly true’; and  5= ‘Completely true’. |
| 36 | Interest taking | IAF^6^ (15) | Interest (5) | Discrete quantitative, where higher scores indicate greater autonomous interest-taking. | 1) Sum the ratings for items 3, 5, 9, 12, and 13, using the scale below.  1= ‘Not at all true’;  2= ‘A bit true’;  3= ‘Somewhat true’;  4= ‘Mostly true’; and  5= ‘Completely true’. |
| 37 | IAF Total | IAF^6^ (15) | IAF^9^ (15) | Discrete quantitative, where higher scores indicate greater autonomy. | 1) Sum the ratings for items 1, 2r^2^, 3, 4, 5, 6r^2^, 7r^2^, 8, 9, 10, 11r^2^, 12, 13, 14r^2^, and 15, using the scale below.  1= ‘Not at all true’;  2= ‘A bit true’;  3= ‘Somewhat true’;  4= ‘Mostly true’; and  5= ‘Completely true’. |
| 38 | Self-reflection | SRI^7^ (20) | Engagement in Self reflection (6) | Discrete quantitative, where higher ratings indicate greater and more frequent reflection and insight skills | 1) Sum the ratings for items 1r^2^, 2r^2^, 3, 4r^2^, 5, and 6; and  2) Divide by the total number of items, namely, 6.  1= ’Strongly disagree’;  2= ’Moderately disagree’;  3=’Slightly disagree’; 4=’Slightly agree’; 5=’Moderately agree’; and  6= ‘Strongly agree’. |
| 39 | Need Reflection | SRI^7^ (20) | Need for self reflection (6) | Discrete quantitative, where higher ratings indicate greater and more frequent reflection and insight skills | 1) Sum the ratings for items 7r^2^, 8, 9, 10, 11, 12 , using the scale below; and  2) Divide by the total number of items, namely, 6.  1= ’Strongly disagree’;  2= ’Moderately disagree’;  3=’Slightly disagree’; 4=’Slightly agree’; 5=’Moderately agree’; and  6= ‘Strongly agree’. |
| 40 | Insight | SRI^7^ (20) | Insight (8) | Discrete quantitative, where higher ratings indicate greater and more frequent reflection and insight skills | 1) Sum the ratings for items 13, 14r^2^, 15, 16r^2^, 17r^2^, 18r^2^, 19r^2^, and 20, using the scale below; and  2) Divide by the total number of items, namely, 8.  1= ’Strongly disagree’;  2= ’Moderately disagree’;  3=’Slightly disagree’; 4=’Slightly agree’; 5=’Moderately agree’; and  6= ‘Strongly agree’. |
| 42 | SRI Total | SRI^7^ (20) | SRI Total (20) | Discrete quantitative, where higher ratings indicate greater and more frequent reflection and insight skills | 1) Sum the ratings for every scale rating, namely, for items 1r^2^, 2r^2^, 3, 4r^2^, 5, 6, 7r^2^, 8, 9, 10, 11, 12, 13, 14r^2^, 15, 16r^2^, 17r^2^, 18r^2^, 19r^2^, and 20, using the scale below; and    2) Divide by the total number of scale items, namely, 20.  1= ’Strongly disagree’;  2= ’Moderately disagree’;  3=’Slightly disagree’; 4=’Slightly agree’; 5=’Moderately agree’; and  6= ‘Strongly agree’. |
| 43 | Inattention | ADHDRS-I^8^ (18) | Inattention/Memory Problems (9) | Discrete quantitative, where higher ratings indicate more and more severe symptoms of ADHD. | Sum the ratings for items 1, 6, 7, 8, 11, 12, 15, 17, and 18, using the scale below.  0= ‘Not at all, never’;  1= ‘Just a little, once in a while’;  2= ‘Pretty much, often’; and  3= ‘Very much, frequently’. |
| 44 | Hyperactivity | ADHDRS-I^8^ (18) | Hyperactivity/Restlessness (9) | Discrete quantitative, where higher ratings indicate more and more severe symptoms of ADHD. | Sum the ratings 2, 3, 4, 5, 9, 10, 13, 14, and 16, using the scale below.  0= ‘Not at all, never’;  1= ‘Just a little, once in a while’;  2= ‘Pretty much, often’; and  3= ‘Very much, frequently’. |
| 45 | ADHDRS_Total | ADHDRS-I^8^ (18) | ADHDRS^5^ (18) | Discrete quantitative, where higher ratings indicate more and more severe symptoms of ADHD. | Sum the ratings to every item, namely, items 1, 2, 3, 4, 5, 6, 7, 8, 9, 10, 11, 12, 13, 14, 15, 16, 17, and 18, using the scale below.  0= ‘Not at all, never’;  1= ‘Just a little, once in a while’;  2= ‘Pretty much, often’; and  3= ‘Very much, frequently’. |
| 46 | PQ P1, week 1 to week 10 | PQ^9^ (100) | Problem 1 (10) | Ordinal, where higher ratings indicate greater difficulties | Ten weekly assessments of P1, using the scale below.  1= ‘Not at all’;  2= ‘Very little’;  3= ‘Little’;  4= ‘Moderately’;  5= ‘Considerably’;  6= ‘Very considerably’; and  7=’Maximum possible’. |
| 47 | PQ P2, week 1 to week 10 | PQ^9^ (100) | Problem 2 (10) | Ordinal, where higher ratings indicate greater difficulties | Ten weekly assessments of P2 using the scale below.  1= ‘Not at all’;  2= ‘Very little’;  3= ‘Little’;  4= ‘Moderately’;  5= ‘Considerably’;  6= ‘Very considerably’; and  7=’Maximum possible’. |
| 48 | P3, week 1 to week 10 | PQ (100) | Problem 3 (10) | Ordinal, where higher ratings indicate greater difficulties | Ten weekly assessments of P3 using the scale below.  1= ‘Not at all’;  2= ‘Very little’;  3= ‘Little’;  4= ‘Moderately’;  5= ‘Considerably’;  6= ‘Very considerably’; and  7=’Maximum possible’. |
| 49 | P4, week 1 to week 10 | PQ^9^ (100) | Problem 4 (10) | Ordinal, where higher ratings indicate greater difficulties | Ten weekly assessments of P4, using the scale below.  1= ‘Not at all’;  2= ‘Very little’;  3= ‘Little’;  4= ‘Moderately’;  5= ‘Considerably’;  6= ‘Very considerably’; and  7=’Maximum possible’. |
| 50 | P5, week 1 to week 10 | PQ^9^ (100) | Problem 5 (10) | Ordinal, where higher ratings indicate greater difficulties | Ten weekly assessments of P5, using the scale below.  1= ‘Not at all’;  2= ‘Very little’;  3= ‘Little’;  4= ‘Moderately’;  5= ‘Considerably’;  6= ‘Very considerably’; and  7=’Maximum possible’. |
| 51 | P6, week 1 to week 10 | PQ^9^ (100) | Problem 6 (10) | Ordinal, where higher ratings indicate greater difficulties | Ten weekly assessments of 6, using the scale below.  1= ‘Not at all’;  2= ‘Very little’;  3= ‘Little’;  4= ‘Moderately’;  5= ‘Considerably’;  6= ‘Very considerably’; and  7=’Maximum possible’. |
| 51 | P7, week 1 to week 10 | PQ^9^ (100) | Problem 7 (10) | Ordinal, where higher ratings indicate greater difficulties | Ten weekly assessments of P7 using the scale below.  1= ‘Not at all’;  2= ‘Very little’;  3= ‘Little’;  4= ‘Moderately’;  5= ‘Considerably’;  6= ‘Very considerably’; and  7=’Maximum possible’. |
| 53 | P8, week 1 to week 10 | PQ (100) | Problem 8 (10) | Ordinal, where higher ratings indicate greater difficulties | Ten weekly assessments of P8 using the scale below.  1= ‘Not at all’;  2= ‘Very little’;  3= ‘Little’;  4= ‘Moderately’;  5= ‘Considerably’;  6= ‘Very considerably’; and  7=’Maximum possible’. |
| 54 | P9, week 1 to week 10 | PQ^9^ (100) | Problem 9 (10) | Ordinal, where higher ratings indicate greater difficulties | Ten weekly assessments of P9, using the scale below.  1= ‘Not at all’;  2= ‘Very little’;  3= ‘Little’;  4= ‘Moderately’;  5= ‘Considerably’;  6= ‘Very considerably’; and  7=’Maximum possible’. |
| 55 | P10, week 1 to week 10 | PQ^9^ (100) | Problem 10 (10) | Ordinal, where higher ratings indicate greater difficulties | Ten weekly assessments of P10, using the scale below.  1= ‘Not at all’;  2= ‘Very little’;  3= ‘Little’;  4= ‘Moderately’;  5= ‘Considerably’;  6= ‘Very considerably’; and  7=’Maximum possible’. |
| 56 | Median Moment 1 (T1) | PQ^9^ (100) | MeT1 (10) | Scale, where higher ratings indicate greater distress | Median of ratings for T1, across rated problems. |
| 57 | Median Moment 2 (T2) | PQ^9^ (100) | MeT2 (10) | Scale, where higher ratings indicate greater distress | Median of ratings for T2, across rated problems. |
| 58 | Median Moment 3 (T3) | PQ^9^ (100) | MeT3 (10) | Scale, where higher ratings indicate greater distress | Median of ratings for T3, across rated problems. |
| 59 | Median Moment 4 (T4) | PQ^9^ (100) | MeT4 (10) | Scale, where higher ratings indicate greater distress | Median of ratings for T4, across rated problems. |
| 60 | Median Moment 5 (T5) | PQ^9^ (100) | MeT5 (10) | Scale, where higher ratings indicate greater distress | Median of ratings for T5, across rated problems. |
| 61 | Median Moment 6 (T6) | PQ^9^ (100) | MeT6 (10) | Scale, where higher ratings indicate greater distress | Median of ratings for T6, across rated problems. |
| 61 | Median Moment 7 (T7) | PQ^9^ (100) | MeT7 (10) | Scale, where higher ratings indicate greater distress | Median of ratings for T7, across rated problems. |
| 62 | Median Moment 8 (T8) | PQ^9^ (100) | MeT8(10) | Scale, where higher ratings indicate greater distress | Median of ratings for T8, across rated problems. |
| 63 | Median Moment 9 (T9) | PQ^9^ (100) | MeT9 (10) | Scale, where higher ratings indicate greater distress | Median of ratings for T9, across rated problems. |
| 64 | Median Moment 10 (T10) | PQ^9^ (100) | MeT10 (10) | Scale, where higher ratings indicate greater distress | Median of ratings for T10, across rated problems. |
|  | Notes:  1. N/A = Non-Applicable  2. r = Reversed scale item  3. EQ-5D-5L = EuroQuom - five Dimensions - five Levels  4. AAQoL = Attention Deficit Hyperactivity Disorder Quality of Life Scale  5. CORE-OM = Clinical Outcomes in Routine Evaluation – Outcome Measure  6. IAF = Index of Autonomous Functioning  7. SRI = Self-Reflection and Insight Scale  8. ADHDRS/ADHDRSI = Attention Deficit Hyperactivity Disorder Rating Scale, investigator rated.  9. PQ = Personal Questionnaire | | | | |

**Table 2:** Procedures for the calculation of the Index value of EQ-5D-5L

| **Data set of weights for the health profile dimensions** | **SPSS Syntax Commands^1, 2^** |
| --- | --- |
| English (ENG) Devlin value set Version 1.2 (Updated 31/08/2022) | ******************************************************************  *SPSS syntax code for the computation of index*  *values with ENG TTO value set*  ******************************************************************;  IF (mobility=1) disut_mo=0.  IF (mobility=2) disut_mo=0.058.  IF (mobility=3) disut_mo=0.076.  IF (mobility=4) disut_mo=0.207.  IF (mobility=5) disut_mo=0.274.  IF (selfcare=1) disut_sc=0.  IF (selfcare=2) disut_sc=0.050.  IF (selfcare=3) disut_sc=0.080.  IF (selfcare=4) disut_sc=0.164.  IF (selfcare=5) disut_sc=0.203.  IF (activity=1) disut_ua=0.  IF (activity=2) disut_ua=0.050.  IF (activity=3) disut_ua=0.063.  IF (activity=4) disut_ua=0.162.  IF (activity=5) disut_ua=0.184.  IF (pain=1) disut_pd=0.  IF (pain=2) disut_pd=0.063.  IF (pain=3) disut_pd=0.084.  IF (pain=4) disut_pd=0.276.  IF (pain=5) disut_pd=0.335.  IF (anxiety=1) disut_ad=0.  IF (anxiety=2) disut_ad=0.078.  IF (anxiety=3) disut_ad=0.104.  IF (anxiety=4) disut_ad=0.285.  IF (anxiety=5) disut_ad=0.289.  Compute disut_total= disut_mo +disut_sc +disut_ua +disut_pd +disut_ad.  Compute EQindex = 1-disut_total.  Formats EQindex(F8.3).  execute. |
| Notes:   1. The variables for the 5 dimensions of the EQ-5D-5L descriptive system should be named 'mobility', 'selfcare', 'activity', 'pain', and 'anxiety'. If they are given different names the syntax code below will not work properly. 2. The 5 variables should contain the values for the different dimensions in the EQ-5D health profile (i.e. 1, 2, 3, 4 or 5). | |

# Descriptive statistics

## Demographics

**Table 3:** Descriptive statistics for gender, race groups, diagnosis, date of diagnosis group and medication for the whole sample


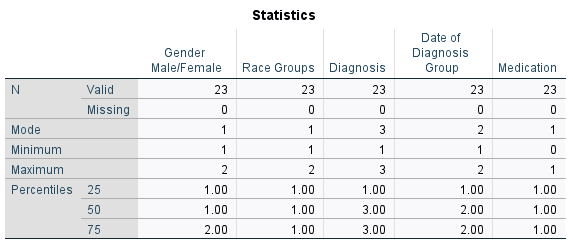


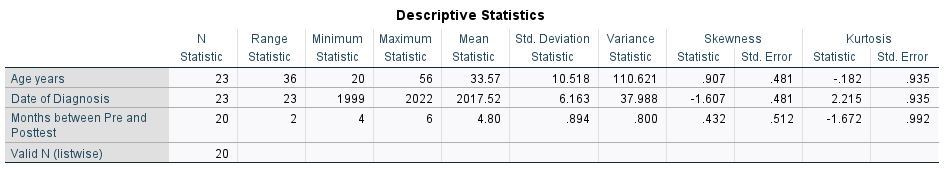
**Table 4:** Descriptive statistics for age and date of diagnosis for the whole sample

**Table 5:** Descriptive statistics for gender, race groups, diagnosis, date of diagnosis group and medication for the Intervention group


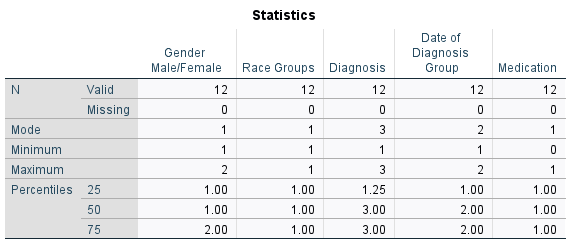


**Table 6:** Descriptive statistics for gender, race groups, diagnosis, date of diagnosis group and medication for the Intervention group


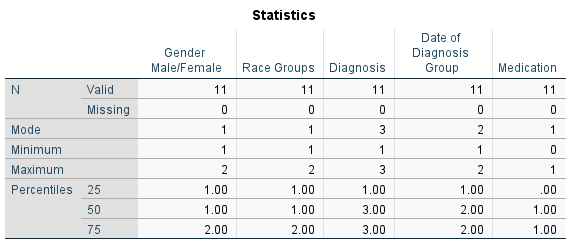


**Table 7:** Descriptive statistics for age and date of diagnosis for the Intervention group


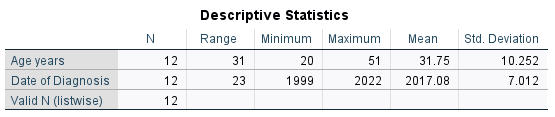


**Table 8:** Descriptive statistics for age and date of diagnosis for the Control group


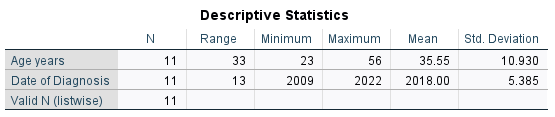


**Table 9:** Descriptive statistics (Counts and expected counts) in both groups, and overall, for qualitative variables

| **Group**  **Characteristic** | **Intervention** | **Expected Count** | **Control** | **Expected Count** | **Total** | **Expected Count** |
| --- | --- | --- | --- | --- | --- | --- |
| **Size** | 12 | 12 | 11 | 11 | 23 | 23 |
| **Gender** |  |  |  |  |  |  |
| **Male** | 7 | 6.8 | 6 | 6.2 | 13 | 13 |
| **Female** | 4 | 4.42 | 4 | 3.8 | 8 | 88 |
| **Transgender** | 1 | 1 | 1 | 1 | 2 | 2 |
| **Total** | 12 | 12 | 11 | 11 | 23 |  |
| **Age** |  |  |  |  |  |  |
| **33 or less** | 9 | 8.3 | 7 | 7.7 | 16 | 16 |
| **More than 33** | 3 | 3.7 | 4 | 3.3 | 7 | 7 |
| **Total** | 12 | 12 | 11 | 11 | 23 | 23 |
| **Race** |  |  |  |  |  |  |
| **White British** | 12 | 10.4 | 8 | 9.6 | 20 | 20 |
| **Not White British** | 0 | 1.6 | 3 | 1.4 | 3 | 3 |
| **Total** | 12 | 12 | 11 | 11 | 23 | 23 |
| **ADHD subtype** |  |  |  |  |  |  |
| **Inattentive** | 3 | 3.7 | 4 | 3.3 | 7 | 7 |
| **Hyperactive** | 1 | 1 | 1 | 1 | 2 | 2 |
| **Combined** | 8 | 7.3 | 6 | 6.7 | 14 | 14 |
| **Total** | 12 | 12 | 11 | 11 | 23 | 23 |
| **Date of Diagnosis** |  |  |  |  |  |  |
| **Before 2020** | 4 | 4.2 | 4 | 3.8 | 8 | 8 |
| **During or after 2020** | 8 | 7.8 | 7 | 7.2 | 15 | 15 |
| **Total** | 12 | 12 | 11 | 11 | 23 | 23 |
| **Medication** |  |  |  |  |  |  |
| **None** | 2 | 2.6 | 3 | 2.4 | 5 | 5 |
| **Prescribed** | 10 | 9.4 | 8 | 8.6 | 18 | 18 |
| **Total** | 12 | 12 | 11 | 11 | 23 | 23 |

**Table 10:** Spearman correlations between dropouts and demographic and clinical attributes

|  | | | **Participant (1) / Dropout (2)** | **Gender Male/Female/Transgender** | **Age years** | **Race Groups** | **Diagnosis** | **Date of Diagnosis** | **Medication** |
| --- | --- | --- | --- | --- | --- | --- | --- | --- | --- |
| **Spearman's rho** | **Gender Male/**  **Female/Trans gender** | **Correlation Coefficient** | -.099 |  |  |  |  |  |  |
|  |  | **Sig. (2-tailed)** | .652 |  |  |  |  |  |  |
|  |  | **N** | 23 |  |  |  |  |  |  |
|  | **Age years** | **Correlation Coefficient** | -.039 | -.109 |  |  |  |  |  |
|  |  | **Sig. (2-tailed)** | .860 | .619 |  |  |  |  |  |
|  |  | **N** | 23 | 23 |  |  |  |  |  |
|  | **Race Groups** | **Correlation Coefficient** | -.150 | .011 | .351 |  |  |  |  |
|  |  | **Sig. (2-tailed)** | .495 | .960 | .101 |  |  |  |  |
|  |  | **N** | 23 | 23 | 23 |  |  |  |  |
|  | **Diagnosis** | **Correlation Coefficient** | .124 | -.002 | .035 | .023 |  |  |  |
|  |  | **Sig. (2-tailed)** | .573 | .994 | .874 | .919 |  |  |  |
|  |  | **N** | 23 | 23 | 23 | 23 |  |  |  |
|  | **Date of Diagnosis** | **Correlation Coefficient** | -.010 | .230 | -.332 | -.279 | .277 |  |  |
|  |  | **Sig. (2-tailed)** | .964 | .291 | .122 | .197 | .200 |  |  |
|  |  | **N** | 23 | 23 | 23 | 23 | 23 |  |  |
|  | **Medication** | **Correlation Coefficient** | -.422^*^ | -.117 | .326 | .204 | -.037 | -.163 |  |
|  |  | **Sig. (2-tailed)** | .045 | .595 | .129 | .350 | .868 | .458 |  |
|  |  | **N** | 23 | 23 | 23 | 23 | 23 | 23 |  |
|  | **Months between Pre and Post-test** | **Correlation Coefficient** | . | -.275 | .013 | -.013 | .118 | .111 | -.053 |
|  |  | **Sig. (2-tailed)** | . | .241 | .957 | .956 | .622 | .642 | .824 |
|  |  | **N** | 20 | 20 | 20 | 20 | 20 | 20 | 20 |

**Table 11:** Confidence Intervals (BLA) for Spearman correlations between demographic and clinical attributes

|  | **Spearman's rho** | **Significance(2-tailed)** | **95% Confidence Intervals (2-tailed)^a,b^** | |
| --- | --- | --- | --- | --- |
|  |  |  | **Lower** | **Upper** |
| **Groups with dropouts in one category - Gender Male/Female/Transgender** | -.099 | .652 | -.501 | .338 |
| **Groups with dropouts in one category - Age years** | -.039 | .860 | -.454 | .390 |
| **Groups with dropouts in one category - Race Groups** | -.150 | .495 | -.539 | .291 |
| **Groups with dropouts in one category - Diagnosis** | .124 | .573 | -.316 | .520 |
| **Groups with dropouts in one category - Date of Diagnosis** | -.010 | .964 | -.431 | .415 |
| **Groups with dropouts in one category - Medication** | -.422 | .045 | -.717 | .001 |
| **Groups with dropouts in one category - Months between Pre and Post-test** | .^c^ | . | . | . |
| **Gender Male/Female/Transgender - Age years** | -.109 | .619 | -.509 | .329 |
| **Gender Male/Female/Transgender - Race Groups** | .011 | .960 | -.414 | .432 |
| **Gender Male/Female/Transgender - Diagnosis** | -.002 | .994 | -.424 | .422 |
| **Gender Male/Female/Transgender - Date of Diagnosis** | .230 | .291 | -.214 | .595 |
| **Gender Male/Female/Transgender - Medication** | -.117 | .595 | -.515 | .322 |
| **Gender Male/Female/Transgender - Months between Pre and Post-test** | -.275 | .241 | -.648 | .204 |
| **Age years - Race Groups** | .351 | .101 | -.085 | .674 |
| **Age years - Diagnosis** | .035 | .874 | -.394 | .451 |
| **Age years - Date of Diagnosis** | -.332 | .122 | -.662 | .106 |
| **Age years - Medication** | .326 | .129 | -.112 | .658 |
| **Age years - Months between Pre and Post-test** | .013 | .957 | -.444 | .464 |
| **Race Groups - Diagnosis** | .023 | .919 | -.404 | .441 |
| **Race Groups - Date of Diagnosis** | -.279 | .197 | -.628 | .163 |
| **Race Groups - Medication** | .204 | .350 | -.239 | .577 |
| **Race Groups - Months between Pre and Post-test** | -.013 | .956 | -.464 | .443 |
| **Diagnosis - Date of Diagnosis** | .277 | .200 | -.165 | .627 |
| **Diagnosis - Medication** | -.037 | .868 | -.453 | .392 |
| **Diagnosis - Months between Pre and Post-test** | .118 | .622 | -.355 | .542 |
| **Date of Diagnosis - Medication** | **-.163** | **.458** | **-.548** | **.279** |
| **Date of Diagnosis - Months between Pre and Post-test** | **.111** | **.642** | **-.361** | **.538** |
| **Medication - Months between Pre and Post-test** | **-.053** | **.824** | **-.495** | **.410** |
| a. Estimation is based on Fisher's r-to-z transformation. | | | | |
| b. Estimation of standard error is based on the formula proposed by Fieller, Hartley, and Pearson. | | | | |
| c. Cannot be computed because at least one of the variables is constant. | | | | |

## EQ-5D-5L: Profiles - Descriptive statistics results

**Figure 5:** Frequencies of profiles at pre- and post-test, by group


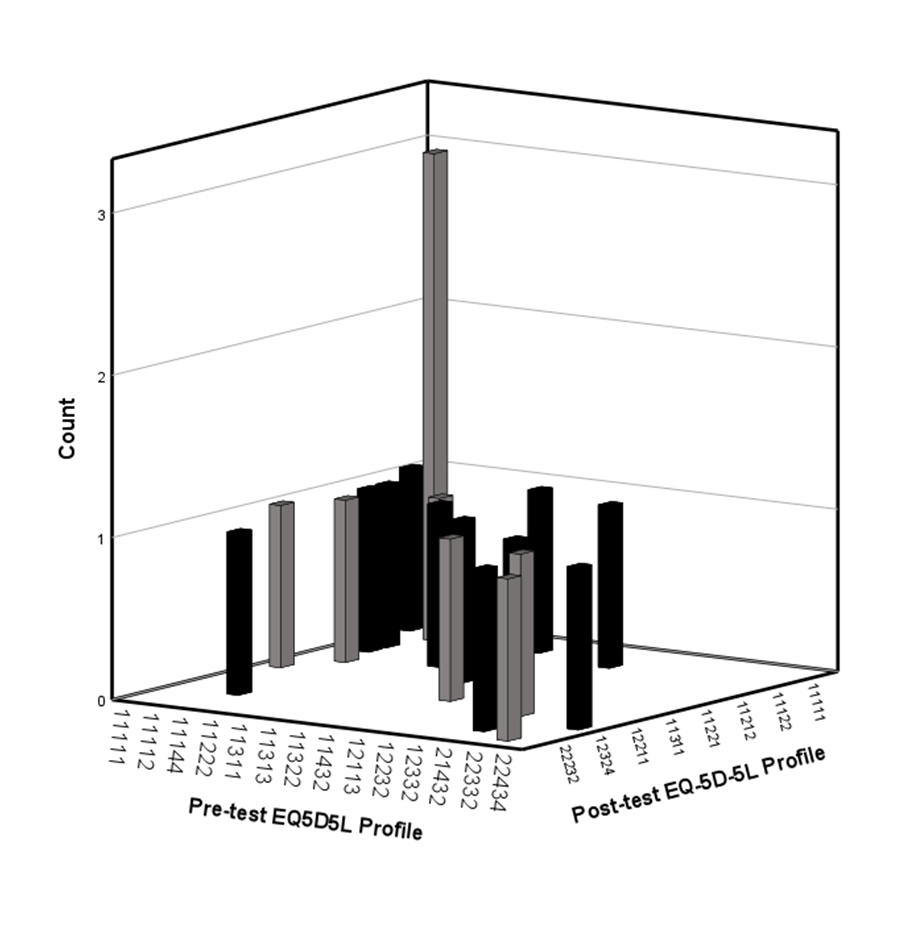


## EQ-5D-5L: Descriptive statistics results


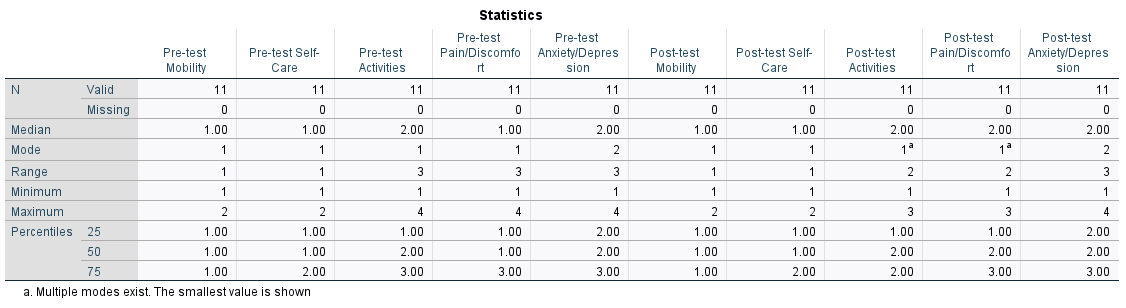
**Table 12:** Descriptive statistics for the dimensions of EQ-5D-5L in the Intervention group, at pre- and post-test


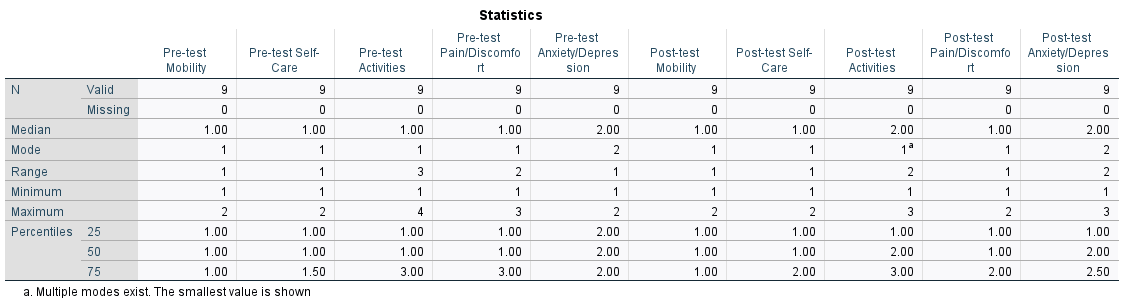
**Table 13:** Descriptive statistics for the dimensions of EQ-5D-5L in the Control group, at pre- and post-test

**Table 14:** Case summaries for profiles at pre- and post-test, per group**
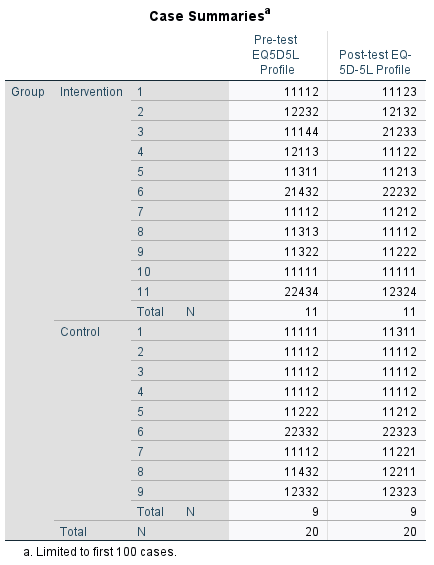
**

**Table 15:** Profile frequencies, by group and assessment moment


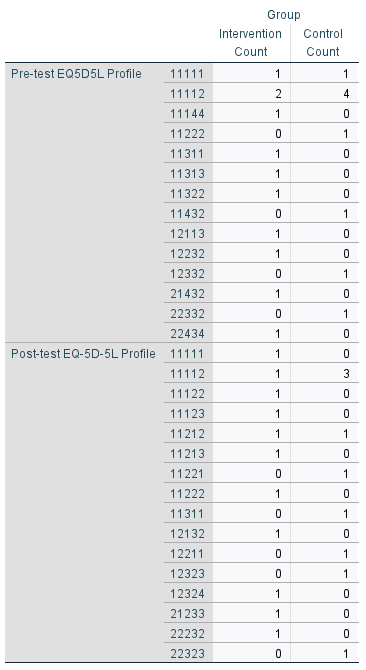


**Table 16:** Profile frequencies and cumulative percents, in the Intervention group, at pre-test


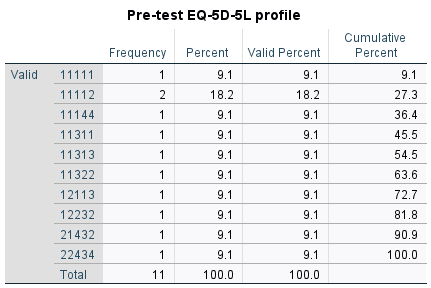


**Table 17:** Profile frequencies and cumulative percents, in the Intervention group, at post-test

**
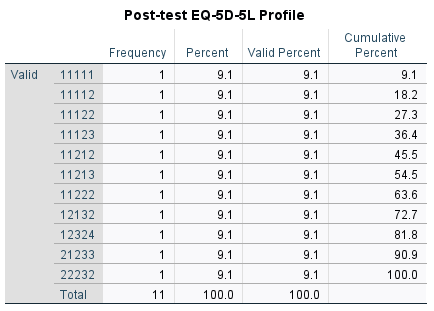
**

**Table 18:** Profile frequencies and cumulative percents, at pre-test, in the Control group.


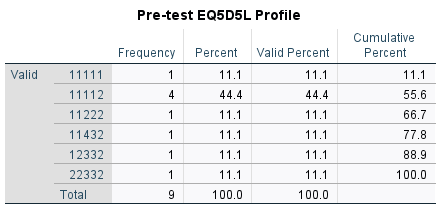


**Table 19:** Profile frequencies and cumulative percents, at post-test, in the Control group


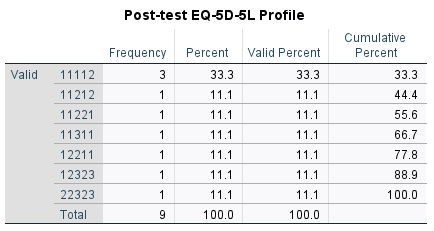


**Table 20:** Descriptive statistics for the EQ5D5L’s Total and Index value in the Intervention group at pre- and post-test


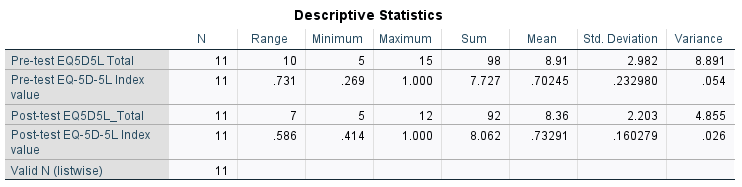


**Table 21:** Descriptive statistics for the EQ5D5L’s Total and Index value in the Control group at pre- and post-test


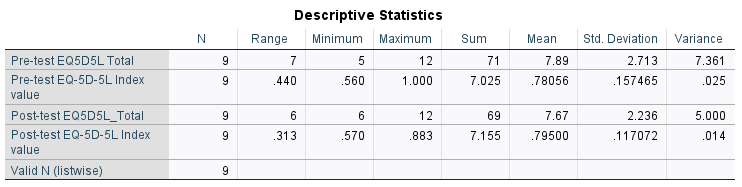


## CORE-OM: Descriptive statistics

**Table 22:** Descriptive statistics for CORE-OM measures in the Intervention group, pre- and post-test

**
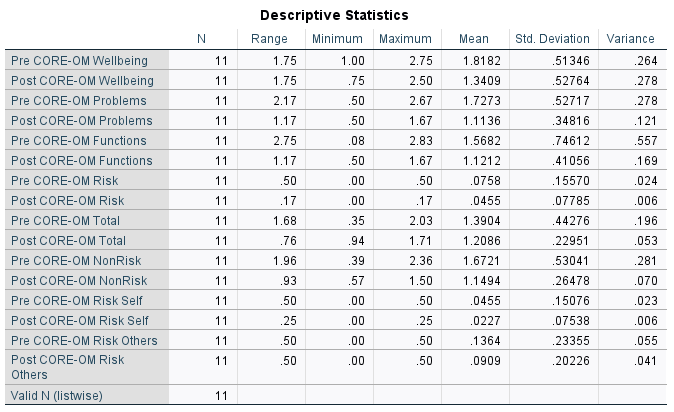
**

**Table 23:** Descriptive statistics for CORE-OM measures in the Control group, pre- and post-test


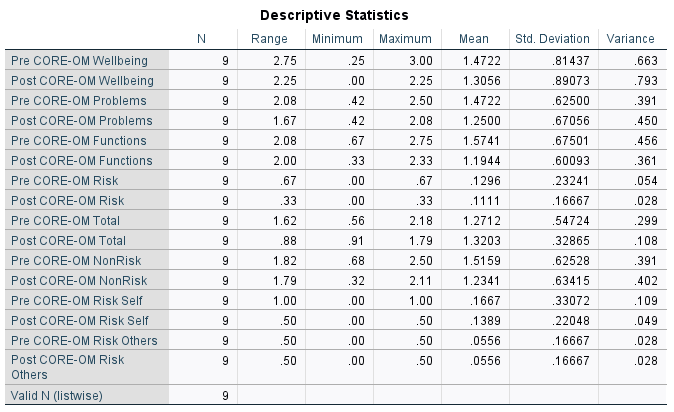


## ADHDRS: Descriptive Statistics

**Table 24:** Descriptive statistics for the items of ADHDRS in the Intervention group at pre-and post-test (quartiles)

|  | N | | Percentiles | | |
| --- | --- | --- | --- | --- | --- |
|  | Valid | Missing | 25 | 50 | 75 |
| Q1 Pre- Loses things necessary for tasks and activities | 11 | 0 | 1.00 | 2.00 | 3.00 |
| Q2 Pre - Talks too much | 11 | 0 | 1.00 | 3.00 | 3.00 |
| Q3 Pre - Gets rowdy or boisterous doing leisure activities | 11 | 0 | 1.00 | 1.00 | 3.00 |
| Q4 Pre - Leaves seat when not supposed to | 11 | 0 | .00 | 2.00 | 2.00 |
| Q5 Pre - Has trouble waiting in line or taking turns with others | 11 | 0 | .00 | 2.00 | 2.00 |
| Q6 Pre - Has trouble keeping attention focused when working or at leisure | 11 | 0 | 1.00 | 2.00 | 3.00 |
|  | N | |  | Percentiles |  |
|  | Valid | Missing | 25 | 50 | 75 |
| Q7 Pre - Is forgetful in daily activities | 11 | 0 | 2.00 | 2.00 | 3.00 |
| Q8 Pre - Has trouble listening to what other people are saying | 11 | 0 | 1.00 | 2.00 | 2.00 |
| Q9 Pre - Is always ‘on the go’ | 11 | 0 | 2.00 | 2.00 | 3.00 |
| Q10 Pre - Fidgets with hands or feet or squirms in seat | 11 | 0 | 2.00 | 2.00 | 3.00 |
| Q11 Pre - makes careless mistakes or has trouble paying close attention to detail | 11 | 0 | 1.00 | 2.00 | 3.00 |
| Q12 Pre - Does not like academic studies/work projects where effort at thinking a lot is required | 11 | 0 | .00 | 1.00 | 3.00 |
| Q13 Pre - Is restless or overactive | 11 | 0 | 2.00 | 2.00 | 3.00 |
| Q14 Pre - Gives answers to questions before the questions have been completed | 11 | 0 | 1.00 | 2.00 | 3.00 |
| Q15 Pre - Has trouble finishing job tasks or school work | 11 | 0 | 1.00 | 2.00 | 3.00 |
| Q16 Pre - Interrupts others when they are working or busy | 11 | 0 | 1.00 | 2.00 | 3.00 |
| Q17 Pre - Appears distracted when things are going on around him/her | 11 | 0 | 2.00 | 2.00 | 3.00 |
| Q18 Pre - Has problems organising tasks and activities | 11 | 0 | 1.00 | 2.00 | 3.00 |
| Q1 Post - Loses things necessary for tasks and activities | 11 | 0 | 1.00 | 1.00 | 2.00 |
| Q2 Post - Talks too much | 11 | 0 | 1.00 | 2.00 | 2.00 |
| Q3 Post - Gets rowdy or boisterous doing leisure activities | 11 | 0 | .00 | 1.00 | 2.00 |
| Q4 Post - Leaves seat when not supposed to | 11 | 0 | .00 | 1.00 | 1.00 |
|  |  |  |  |  |  |
|  | N | |  | Percentiles |  |
|  | Valid | Missing | 25 | 50 | 75 |
| Q5 Post - Has trouble waiting in line or taking turns with others | 11 | 0 | .00 | 1.00 | 2.00 |
| Q6 Post - Has trouble keeping attention focused when working or at leisure | 11 | 0 | 1.00 | 1.00 | 2.00 |
| Q7 Post - Is forgetful in daily activities | 11 | 0 | 1.00 | 2.00 | 2.00 |
| Q8 Post - Has trouble listening to what other people are saying | 11 | 0 | 1.00 | 2.00 | 2.00 |
| Q9 Post - Is always ‘on the go’ | 11 | 0 | 1.00 | 2.00 | 2.00 |
| Q10 Post - Fidgets with hands or feet or squirms in seat | 11 | 0 | 1.00 | 2.00 | 3.00 |
| Q11 Post - makes careless mistakes or has trouble paying close attention to detail | 11 | 0 | 1.00 | 1.00 | 2.00 |
| Q12 Post - Does not like academic studies/work projects where effort at thinking a lot is required | 11 | 0 | 1.00 | 1.00 | 2.00 |
| Q13 Post - Is restless or overactive | 11 | 0 | 1.00 | 1.00 | 2.00 |
| Q14 Post - Gives answers to questions before the questions have been completed | 11 | 0 | 1.00 | 2.00 | 3.00 |
| Q15 Post - Has trouble finishing job tasks or school work | 11 | 0 | 1.00 | 1.00 | 2.00 |
| Q16 Post - Interrupts others when they are working or busy | 11 | 0 | .00 | 1.00 | 2.00 |
| Q17 Post - Appears distracted when things are going on around him/her | 11 | 0 | 1.00 | 2.00 | 3.00 |
| Q18 Post - Has problems organising tasks and activities | 11 | 0 | 1.00 | 1.00 | 2.00 |

**Table 25:** Descriptive statistics for the items of ADHDRS in the Control group at pre-and post-test (quartiles)

|  | N | | Percentiles | | |
| --- | --- | --- | --- | --- | --- |
|  | Valid | Missing | 25 | 50 | 75 |
| Q1 Pre- Loses things necessary for tasks and activities | 9 | 0 | 1.00 | 2.00 | 3.00 |
| Q2 Pre - Talks too much | 9 | 0 | 1.50 | 2.00 | 3.00 |
| Q3 Pre - Gets rowdy or boisterous doing leisure activities | 9 | 0 | .00 | 1.00 | 2.00 |
| Q4 Pre - Leaves seat when not supposed to | 9 | 0 | 1.00 | 2.00 | 2.00 |
| Q5 Pre - Has trouble waiting in line or taking turns with others | 9 | 0 | .50 | 2.00 | 2.00 |
| Q6 Pre - Has trouble keeping attention focused when working or at leisure | 9 | 0 | 2.00 | 3.00 | 3.00 |
| Q7 Pre - Is forgetful in daily activities | 9 | 0 | 1.50 | 2.00 | 3.00 |
| Q8 Pre - Has trouble listening to what other people are saying | 9 | 0 | 1.00 | 2.00 | 3.00 |
| Q9 Pre - Is always ‘on the go’ | 9 | 0 | 1.00 | 2.00 | 3.00 |
| Q10 Pre - Fidgets with hands or feet or squirms in seat | 9 | 0 | 2.50 | 3.00 | 3.00 |
| Q11 Pre - makes careless mistakes or has trouble paying close attention to detail | 9 | 0 | 1.00 | 2.00 | 2.00 |
| Q12 Pre - Does not like academic studies/work projects where effort at thinking a lot is required | 9 | 0 | 1.00 | 2.00 | 2.50 |
| Q13 Pre - Is restless or overactive | 9 | 0 | 1.00 | 2.00 | 3.00 |
| Q14 Pre - Gives answers to questions before the questions have been completed | 9 | 0 | 2.00 | 3.00 | 3.00 |
| Q15 Pre - Has trouble finishing job tasks or school work | 9 | 0 | 1.50 | 2.00 | 3.00 |
| Q16 Pre - Interrupts others when they are working or busy | 9 | 0 | 1.50 | 2.00 | 3.00 |
|  | N | |  | Percentiles |  |
|  | Valid | Missing | 25 | 50 | 75 |
| Q17 Pre - Appears distracted when things are going on around him/her | 9 | 0 | 2.00 | 2.00 | 3.00 |
| Q18 Pre - Has problems organising tasks and activities | 9 | 0 | 1.50 | 3.00 | 3.00 |
| Q1 Post - Loses things necessary for tasks and activities | 9 | 0 | 1.00 | 2.00 | 2.50 |
| Q2 Post - Talks too much | 9 | 0 | 1.50 | 2.00 | 2.00 |
| Q3 Post - Gets rowdy or boisterous doing leisure activities | 9 | 0 | .00 | 1.00 | 2.00 |
| Q4 Post - Leaves seat when not supposed to | 9 | 0 | 1.00 | 2.00 | 2.00 |
| Q5 Post - Has trouble waiting in line or taking turns with others | 9 | 0 | 1.00 | 1.00 | 2.00 |
| Q6 Post - Has trouble keeping attention focused when working or at leisure | 9 | 0 | 1.50 | 2.00 | 3.00 |
| Q7 Post - Is forgetful in daily activities | 9 | 0 | 1.50 | 2.00 | 3.00 |
| Q8 Post - Has trouble listening to what other people are saying | 9 | 0 | 1.00 | 2.00 | 2.50 |
| Q9 Post - Is always ‘on the go’ | 9 | 0 | 1.00 | 1.00 | 2.50 |
| Q10 Post - Fidgets with hands or feet or squirms in seat | 9 | 0 | 1.50 | 3.00 | 3.00 |
| Q11 Post - makes careless mistakes or has trouble paying close attention to detail | 9 | 0 | 1.00 | 2.00 | 2.00 |
| Q12 Post - Does not like academic studies/work projects where effort at thinking a lot is required | 9 | 0 | 1.00 | 1.00 | 3.00 |
| Q13 Post - Is restless or overactive | 9 | 0 | 1.00 | 1.00 | 2.50 |
| Q14 Post - Gives answers to questions before the questions have been completed | 9 | 0 | 1.50 | 2.00 | 3.00 |
|  |  |  |  |  |  |
|  | N | |  | Percentiles |  |
|  | Valid | Missing | 25 | 50 | 75 |
| Q15 Post - Has trouble finishing job tasks or school work | 9 | 0 | 1.50 | 2.00 | 2.00 |
| Q16 Post - Interrupts others when they are working or busy | 9 | 0 | 1.00 | 2.00 | 2.50 |
| Q17 Post - Appears distracted when things are going on around him/her | 9 | 0 | 1.50 | 2.00 | 3.00 |
| Q18 Post - Has problems organising tasks and activities | 9 | 0 | 1.00 | 2.00 | 3.00 |

**Table 26:** Descriptive statistics for the dimensions of ADHDRS in the Intervention group at pre-and post-test


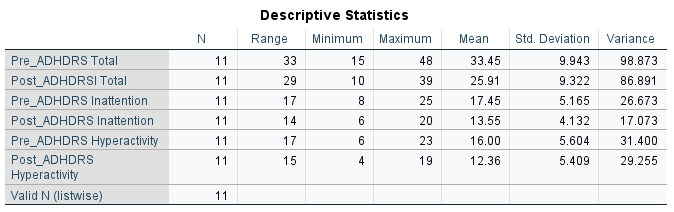


**Table 27:** Descriptive statistics for the dimensions of ADHDRS in the Control group at pre-and post-test


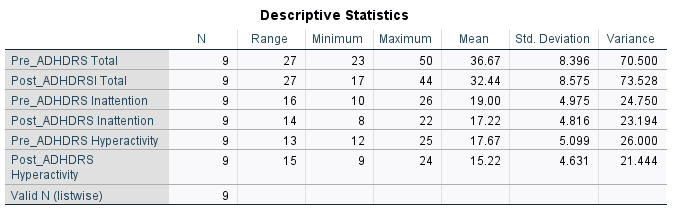


## AAQoL: Descriptive Statistics

**Table 28:** Descriptive Statistics for the dimensions of AAQoL, at pre- and post-test, in the Intervention group


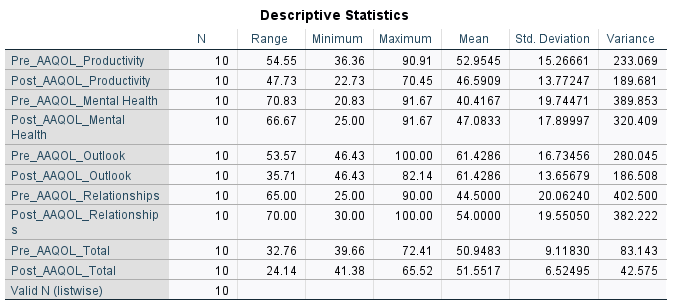


**Table 29:** Descriptive Statistics for the dimensions of AAQoL, at pre- and post-test, in the Control group


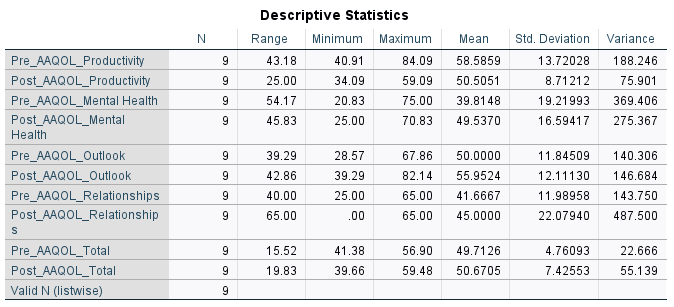


## SRI: Descriptive Results

**Table 30:** Descriptives for pre- and post-test results for the dimensions of SR&I in Intervention group


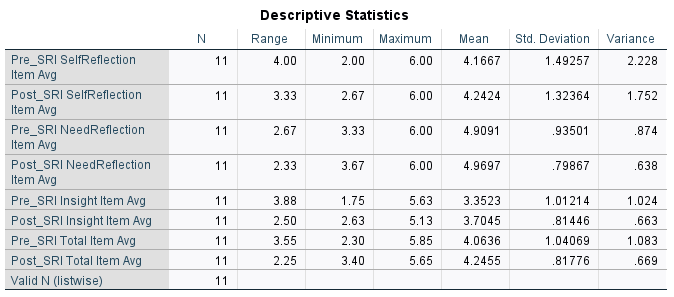


**Table 31:** Descriptives for pre- and post-test results for the dimensions of SRI in Control group


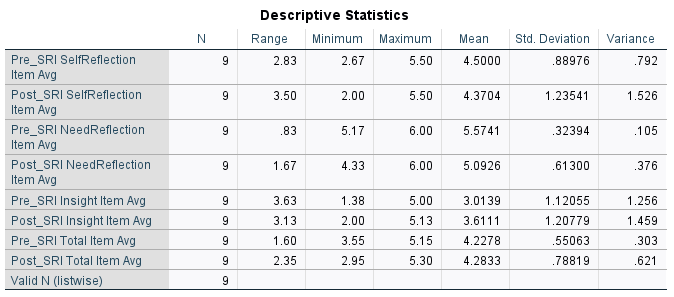


## IAF: Descriptive statistics results

**Table 32:** Descriptives for pre- and post-test results for the dimensions of IAF in Intervention group

**
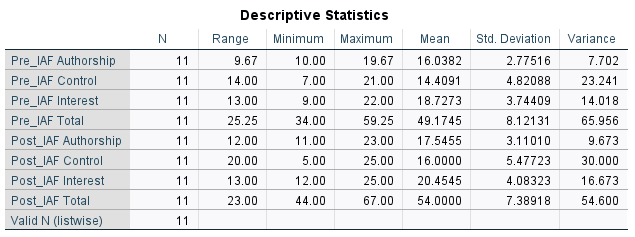
**

**Table 33:** Descriptives for pre- and post-test results for the dimensions of IAF in the Control group

**
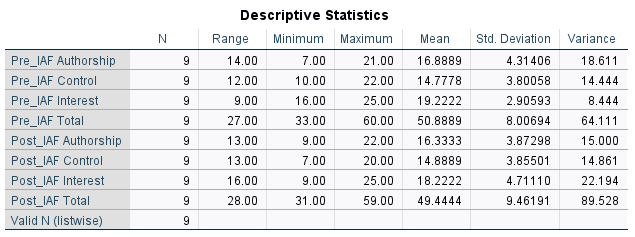
**

## PQ: Descriptive statistics

**Table 34:** Descriptive statistics for ratings to each of the ten problems in the ten weekly assessments, in the Intervention group.

|  | N | | Range | Minimum | Maximum | Percentiles | | |
| --- | --- | --- | --- | --- | --- | --- | --- | --- |
|  | Valid | Missing |  |  |  | 25 | 50 | 75 |
| P1 T2 | 11 | 0 | 4.00 | 2.00 | 6.00 | 3.0000 | 5.0000 | 6.0000 |
| P1 T3 | 11 | 0 | 6.00 | 1.00 | 7.00 | 3.0000 | 4.0000 | 6.0000 |
| P1 T4 | 11 | 0 | 4.00 | 2.00 | 6.00 | 3.0000 | 4.0000 | 6.0000 |
| P1 T5 | 11 | 0 | 4.00 | 2.00 | 6.00 | 4.0000 | 5.0000 | 5.0000 |
| P1 T6 | 11 | 0 | 3.00 | 2.00 | 5.00 | 3.0000 | 4.0000 | 5.0000 |
| P1 T7 | 11 | 0 | 3.00 | 2.00 | 5.00 | 3.0000 | 4.0000 | 4.0000 |
| P1 T8 | 11 | 0 | 2.00 | 3.00 | 5.00 | 3.0000 | 4.0000 | 5.0000 |
| P1 T9 | 11 | 0 | 3.00 | 3.00 | 6.00 | 3.0000 | 4.0000 | 6.0000 |
| P1 T10 | 11 | 0 | 2.00 | 3.00 | 5.00 | 3.0000 | 4.0000 | 5.0000 |
| P2 T1 | 11 | 0 | 6.00 | 1.00 | 7.00 | 3.0000 | 6.0000 | 6.0000 |
| P2 T2 | 11 | 0 | 5.00 | 1.00 | 6.00 | 3.0000 | 5.0000 | 6.0000 |
| P2 T3 | 11 | 0 | 6.00 | 1.00 | 7.00 | 4.0000 | 5.0000 | 5.0000 |
| P2 T4 | 11 | 0 | 5.00 | 2.00 | 7.00 | 4.0000 | 5.0000 | 5.0000 |
| P2 T5 | 11 | 0 | 6.00 | 1.00 | 7.00 | 3.0000 | 5.0000 | 6.0000 |
| P2 T6 | 11 | 0 | 4.00 | 3.00 | 7.00 | 3.0000 | 5.0000 | 5.0000 |
| P2 T7 | 11 | 0 | 3.00 | 3.00 | 6.00 | 3.0000 | 4.0000 | 5.0000 |
| P2 T8 | 11 | 0 | 4.00 | 2.00 | 6.00 | 3.0000 | 4.0000 | 6.0000 |
| P2 T9 | 11 | 0 | 3.00 | 3.00 | 6.00 | 3.0000 | 4.0000 | 6.0000 |
| P2 T10 | 11 | 0 | 3.00 | 3.00 | 6.00 | 4.0000 | 4.0000 | 5.0000 |
| P3 T1 | 11 | 0 | 5.00 | 1.00 | 6.00 | 4.0000 | 5.0000 | 6.0000 |
| P3 T2 | 11 | 0 | 6.00 | 1.00 | 7.00 | 4.0000 | 5.0000 | 5.0000 |
| P3 T3 | 11 | 0 | 5.00 | 1.00 | 6.00 | 3.0000 | 4.0000 | 6.0000 |
| P3 T4 | 11 | 0 | 4.00 | 2.00 | 6.00 | 4.0000 | 4.0000 | 5.0000 |
| P3 T5 | 11 | 0 | 6.00 | 1.00 | 7.00 | 3.0000 | 5.0000 | 5.0000 |
| P3 T6 | 11 | 0 | 4.00 | 2.00 | 6.00 | 4.0000 | 4.0000 | 5.0000 |
| P3 T7 | 11 | 0 | 4.00 | 2.00 | 6.00 | 3.0000 | 4.0000 | 5.0000 |
| P3 T8 | 11 | 0 | 5.00 | 1.00 | 6.00 | 4.0000 | 4.0000 | 5.0000 |
| P3 T9 | 11 | 0 | 3.00 | 3.00 | 6.00 | 3.0000 | 4.0000 | 5.0000 |
| P3 T10 | 11 | 0 | 3.00 | 3.00 | 6.00 | 4.0000 | 5.0000 | 5.0000 |
| P4 T1 | 11 | 0 | 5.00 | 2.00 | 7.00 | 3.0000 | 5.0000 | 6.0000 |
| P4 T2 | 11 | 0 | 5.00 | 2.00 | 7.00 | 4.0000 | 5.0000 | 6.0000 |
| P4 T3 | 11 | 0 | 6.00 | 1.00 | 7.00 | 3.0000 | 5.0000 | 5.0000 |
| P4 T4 | 11 | 0 | 3.00 | 3.00 | 6.00 | 4.0000 | 4.0000 | 5.0000 |
|  | N | |  |  |  | Percentiles | | |
|  | Valid | Missing | Range | Minimum | Maximum | 25 | 50 | 75 |
| P4 T5 | 11 | 0 | 4.00 | 2.00 | 6.00 | 3.0000 | 4.0000 | 6.0000 |
| P4 T6 | 11 | 0 | 4.00 | 3.00 | 7.00 | 3.0000 | 4.0000 | 6.0000 |
| P4 T7 | 11 | 0 | 4.00 | 2.00 | 6.00 | 3.0000 | 4.0000 | 5.0000 |
| P4 T8 | 11 | 0 | 3.00 | 3.00 | 6.00 | 3.0000 | 4.0000 | 5.0000 |
| P4 T9 | 11 | 0 | 3.00 | 3.00 | 6.00 | 3.0000 | 5.0000 | 6.0000 |
| P4 T10 | 11 | 0 | 3.00 | 3.00 | 6.00 | 3.0000 | 5.0000 | 5.0000 |
| P5 T1 | 11 | 0 | 6.00 | 1.00 | 7.00 | 5.0000 | 5.0000 | 7.0000 |
| P5 T2 | 11 | 0 | 6.00 | 1.00 | 7.00 | 4.0000 | 5.0000 | 7.0000 |
| P5 T3 | 11 | 0 | 6.00 | 1.00 | 7.00 | 4.0000 | 6.0000 | 6.0000 |
| P5 T4 | 11 | 0 | 4.00 | 2.00 | 6.00 | 3.0000 | 5.0000 | 6.0000 |
| P5 T5 | 11 | 0 | 6.00 | .00 | 6.00 | 3.0000 | 4.0000 | 6.0000 |
| P5 T6 | 11 | 0 | 6.00 | 1.00 | 7.00 | 4.0000 | 6.0000 | 6.0000 |
| P5 T7 | 11 | 0 | 5.00 | 2.00 | 7.00 | 4.0000 | 5.0000 | 5.0000 |
| P5 T8 | 11 | 0 | 5.00 | 2.00 | 7.00 | 3.0000 | 4.0000 | 6.0000 |
| P5 T9 | 11 | 0 | 6.00 | 1.00 | 7.00 | 3.0000 | 4.0000 | 6.0000 |
| P1 T1 | 11 | 0 | 7.00 | .00 | 7.00 | 4.0000 | 5.0000 | 6.0000 |
| P5 T10 | 11 | 0 | 6.00 | 1.00 | 7.00 | 4.0000 | 4.0000 | 6.0000 |
| P6 T1 | 11 | 0 | 5.00 | 2.00 | 7.00 | 3.0000 | 4.0000 | 6.0000 |
| P6 T2 | 11 | 0 | 6.00 | 1.00 | 7.00 | 4.0000 | 5.0000 | 6.0000 |
| P6 T3 | 11 | 0 | 6.00 | 1.00 | 7.00 | 4.0000 | 5.0000 | 7.0000 |
| P6 T4 | 11 | 0 | 5.00 | 2.00 | 7.00 | 3.0000 | 4.0000 | 6.0000 |
| P6 T5 | 11 | 0 | 6.00 | 1.00 | 7.00 | 3.0000 | 5.0000 | 5.0000 |
| P6 T6 | 11 | 0 | 5.00 | 2.00 | 7.00 | 4.0000 | 5.0000 | 5.0000 |
| P6 T7 | 11 | 0 | 3.00 | 3.00 | 6.00 | 3.0000 | 5.0000 | 6.0000 |
| P6 T8 | 11 | 0 | 5.00 | 1.00 | 6.00 | 3.0000 | 4.0000 | 6.0000 |
| P6 T9 | 11 | 0 | 4.00 | 3.00 | 7.00 | 4.0000 | 4.0000 | 6.0000 |
| P6 T10 | 11 | 0 | 4.00 | 3.00 | 7.00 | 3.0000 | 4.0000 | 5.0000 |
| P7 T1 | 11 | 0 | 5.00 | 1.00 | 6.00 | 5.0000 | 5.0000 | 6.0000 |
| P7 T2 | 11 | 0 | 6.00 | .00 | 6.00 | 4.0000 | 6.0000 | 6.0000 |
| P7 T3 | 11 | 0 | 4.00 | 2.00 | 6.00 | 4.0000 | 5.0000 | 6.0000 |
| P7 T4 | 11 | 0 | 4.00 | 2.00 | 6.00 | 3.0000 | 4.0000 | 6.0000 |
| P7 T5 | 11 | 0 | 6.00 | 1.00 | 7.00 | 3.0000 | 4.0000 | 6.0000 |
| P7 T6 | 11 | 0 | 4.00 | 2.00 | 6.00 | 4.0000 | 5.0000 | 6.0000 |
| P7 T7 | 11 | 0 | 4.00 | 2.00 | 6.00 | 3.0000 | 5.0000 | 6.0000 |
| P7 T8 | 11 | 0 | 5.00 | 2.00 | 7.00 | 4.0000 | 5.0000 | 5.0000 |
| P7 T9 | 11 | 0 | 6.00 | 1.00 | 7.00 | 3.0000 | 5.0000 | 6.0000 |
| P7 T10 | 11 | 0 | 6.00 | 1.00 | 7.00 | 3.0000 | 4.0000 | 5.0000 |
|  | N | |  |  |  | Percentiles | | |
|  | Valid | Missing | Range | Minimum | Maximum | 25 | 50 | 75 |
| P8 T1 | 10 | 1 | 6.00 | 1.00 | 7.00 | 4.2500 | 5.0000 | 6.2500 |
| P8 T2 | 10 | 1 | 6.00 | 1.00 | 7.00 | 3.7500 | 5.0000 | 6.2500 |
| P8 T3 | 10 | 1 | 6.00 | 1.00 | 7.00 | 3.7500 | 5.0000 | 6.2500 |
| P8 T4 | 10 | 1 | 6.00 | 1.00 | 7.00 | 4.0000 | 4.0000 | 5.2500 |
| P8 T5 | 10 | 1 | 7.00 | .00 | 7.00 | 2.7500 | 5.0000 | 5.0000 |
| P8 T6 | 10 | 1 | 5.00 | 1.00 | 6.00 | 3.0000 | 4.5000 | 6.0000 |
| P8 T7 | 10 | 1 | 5.00 | 2.00 | 7.00 | 3.0000 | 4.0000 | 5.2500 |
| P8 T8 | 10 | 1 | 5.00 | 2.00 | 7.00 | 3.0000 | 5.0000 | 6.2500 |
| P8 T9 | 10 | 1 | 5.00 | 1.00 | 6.00 | 3.7500 | 4.0000 | 6.0000 |
| P8 T10 | 9 | 2 | 4.00 | 3.00 | 7.00 | 3.5000 | 4.0000 | 5.5000 |
| P9 T1 | 9 | 2 | 4.00 | 3.00 | 7.00 | 4.0000 | 6.0000 | 6.5000 |
| P9 T2 | 9 | 2 | 7.00 | .00 | 7.00 | 4.0000 | 4.0000 | 5.5000 |
| P9 T3 | 9 | 2 | 3.00 | 3.00 | 6.00 | 3.5000 | 4.0000 | 5.0000 |
| P9 T4 | 9 | 2 | 3.00 | 3.00 | 6.00 | 4.0000 | 4.0000 | 5.5000 |
| P9 T5 | 9 | 2 | 6.00 | 1.00 | 7.00 | 3.5000 | 4.0000 | 5.5000 |
| P9 T6 | 9 | 2 | 3.00 | 3.00 | 6.00 | 3.0000 | 4.0000 | 5.5000 |
| P9 T7 | 9 | 2 | 3.00 | 3.00 | 6.00 | 3.5000 | 4.0000 | 5.5000 |
| P9 T8 | 9 | 2 | 3.00 | 3.00 | 6.00 | 3.5000 | 4.0000 | 5.5000 |
| P9 T9 | 9 | 2 | 3.00 | 3.00 | 6.00 | 3.5000 | 4.0000 | 5.5000 |
| P9 T10 | 9 | 2 | 5.00 | 2.00 | 7.00 | 3.0000 | 4.0000 | 5.0000 |
| P10 T1 | 5 | 6 | 5.00 | 2.00 | 7.00 | 2.5000 | 4.0000 | 7.0000 |
| P10 T2 | 5 | 6 | 6.00 | 1.00 | 7.00 | 2.5000 | 4.0000 | 5.5000 |
| P10 T3 | 5 | 6 | 4.00 | 3.00 | 7.00 | 3.0000 | 5.0000 | 7.0000 |
| P10 T4 | 5 | 6 | 3.00 | 3.00 | 6.00 | 3.0000 | 4.0000 | 5.0000 |
| P10 T5 | 5 | 6 | 4.00 | 2.00 | 6.00 | 3.0000 | 4.0000 | 5.5000 |
| P10 T6 | 5 | 6 | 3.00 | 3.00 | 6.00 | 3.0000 | 5.0000 | 6.0000 |
| P10 T7 | 5 | 6 | 3.00 | 3.00 | 6.00 | 3.0000 | 3.0000 | 5.0000 |
| P10 T8 | 5 | 6 | 4.00 | 3.00 | 7.00 | 3.0000 | 5.0000 | 6.0000 |
| P10 T9 | 5 | 6 | 4.00 | 3.00 | 7.00 | 3.0000 | 3.0000 | 6.0000 |
| P10 T10 | 5 | 6 | 3.00 | 3.00 | 6.00 | 3.0000 | 4.0000 | 5.5000 |

**Table 35:** Descriptive statistics for ratings to each of the ten problems in the ten weekly assessments, in the Control group.

|  | N | | Range | Minimum | Maximum | Percentiles | | |
| --- | --- | --- | --- | --- | --- | --- | --- | --- |
|  | Valid | Missing |  |  |  | 25 | 50 | 75 |
| P1 T2 | 9 | 0 | 3.00 | 3.00 | 6.00 | 4.0000 | 4.0000 | 5.0000 |
| P1 T3 | 9 | 0 | 3.00 | 3.00 | 6.00 | 3.5000 | 4.0000 | 5.0000 |
| P1 T4 | 9 | 0 | 1.00 | 4.00 | 5.00 | 4.0000 | 4.0000 | 5.0000 |
| P1 T5 | 9 | 0 | 2.00 | 3.00 | 5.00 | 3.0000 | 4.0000 | 4.5000 |
| P1 T6 | 9 | 0 | 5.00 | 1.00 | 6.00 | 2.5000 | 4.0000 | 5.0000 |
| P1 T7 | 9 | 0 | 5.00 | 1.00 | 6.00 | 3.0000 | 3.0000 | 4.0000 |
| P1 T8 | 9 | 0 | 4.00 | 1.00 | 5.00 | 2.5000 | 4.0000 | 4.5000 |
| P1 T9 | 9 | 0 | 5.00 | 1.00 | 6.00 | 2.0000 | 3.0000 | 4.5000 |
| P1 T10 | 9 | 0 | 3.00 | 1.00 | 4.00 | 1.6665 | 3.0000 | 4.0000 |
| P2 T1 | 9 | 0 | 2.00 | 4.00 | 6.00 | 4.5000 | 5.0000 | 5.5000 |
| P2 T2 | 9 | 0 | 2.00 | 4.00 | 6.00 | 4.0000 | 4.0000 | 5.0000 |
| P2 T3 | 9 | 0 | 2.00 | 3.00 | 5.00 | 3.5000 | 4.0000 | 4.5000 |
| P2 T4 | 9 | 0 | 3.00 | 3.00 | 6.00 | 3.0000 | 4.0000 | 5.0000 |
| P2 T5 | 9 | 0 | 2.00 | 3.00 | 5.00 | 3.0000 | 4.0000 | 4.5000 |
| P2 T6 | 9 | 0 | 5.00 | 1.00 | 6.00 | 3.0000 | 4.0000 | 4.0000 |
| P2 T7 | 9 | 0 | 4.00 | 1.00 | 5.00 | 2.5000 | 4.0000 | 5.0000 |
| P2 T8 | 9 | 0 | 5.00 | 1.00 | 6.00 | 3.0000 | 4.0000 | 5.0000 |
| P2 T9 | 9 | 0 | 4.00 | 1.00 | 5.00 | 1.5000 | 3.0000 | 4.5000 |
| P2 T10 | 9 | 0 | 3.00 | 1.00 | 4.00 | 1.1665 | 3.0000 | 4.0000 |
| P3 T1 | 9 | 0 | 3.00 | 4.00 | 7.00 | 4.0000 | 6.0000 | 6.0000 |
| P3 T2 | 9 | 0 | 3.00 | 3.00 | 6.00 | 4.0000 | 5.0000 | 5.0000 |
| P3 T3 | 9 | 0 | 3.00 | 3.00 | 6.00 | 3.0000 | 4.0000 | 5.0000 |
| P3 T4 | 9 | 0 | 3.00 | 2.00 | 5.00 | 3.0000 | 4.0000 | 4.5000 |
| P3 T5 | 9 | 0 | 3.00 | 2.00 | 5.00 | 3.0000 | 3.0000 | 4.0000 |
| P3 T6 | 9 | 0 | 5.00 | 1.00 | 6.00 | 2.0000 | 4.0000 | 4.5000 |
| P3 T7 | 9 | 0 | 5.00 | 1.00 | 6.00 | 2.0000 | 4.0000 | 5.0000 |
| P3 T8 | 9 | 0 | 5.00 | 1.00 | 6.00 | 2.0000 | 4.0000 | 4.5000 |
| P3 T9 | 9 | 0 | 4.00 | 1.00 | 5.00 | 2.0000 | 3.0000 | 4.5000 |
| P3 T10 | 9 | 0 | 4.00 | 1.00 | 5.00 | 1.1665 | 3.0000 | 4.0000 |
| P4 T1 | 9 | 0 | 4.00 | 3.00 | 7.00 | 4.5000 | 5.0000 | 7.0000 |
| P4 T2 | 9 | 0 | 3.00 | 4.00 | 7.00 | 4.5000 | 5.0000 | 5.0000 |
| P4 T3 | 9 | 0 | 2.00 | 3.00 | 5.00 | 4.0000 | 4.0000 | 4.5000 |
| P4 T4 | 9 | 0 | 3.00 | 2.00 | 5.00 | 3.0000 | 4.0000 | 5.0000 |
| P4 T5 | 9 | 0 | 2.00 | 3.00 | 5.00 | 3.5000 | 4.0000 | 5.0000 |
|  | N | |  |  |  | Percentiles | | |
|  | Valid | Missing | Range | Minimum | Maximum | 25 | 50 | 75 |
| P4 T6 | 9 | 0 | 4.00 | 1.00 | 5.00 | 3.0000 | 4.0000 | 5.0000 |
| P4 T7 | 9 | 0 | 4.00 | 1.00 | 5.00 | 3.5000 | 4.0000 | 4.0000 |
| P4 T8 | 9 | 0 | 5.00 | 1.00 | 6.00 | 3.0000 | 4.0000 | 4.5000 |
| P4 T9 | 9 | 0 | 4.00 | 1.00 | 5.00 | 2.5000 | 4.0000 | 4.0000 |
| P4 T10 | 9 | 0 | 3.00 | 1.00 | 4.00 | 1.6650 | 3.0000 | 4.0000 |
| P5 T1 | 9 | 0 | 4.00 | 3.00 | 7.00 | 4.5000 | 6.0000 | 6.5000 |
| P5 T2 | 9 | 0 | 4.00 | 1.00 | 5.00 | 4.0000 | 5.0000 | 5.0000 |
| P5 T3 | 9 | 0 | 2.00 | 2.00 | 4.00 | 4.0000 | 4.0000 | 4.0000 |
| P5 T4 | 9 | 0 | 3.00 | 1.00 | 4.00 | 2.5000 | 3.0000 | 4.0000 |
| P5 T5 | 9 | 0 | 3.00 | 2.00 | 5.00 | 3.0000 | 4.0000 | 4.5000 |
| P5 T6 | 9 | 0 | 4.00 | 1.00 | 5.00 | 2.0000 | 4.0000 | 4.5000 |
| P5 T7 | 9 | 0 | 3.00 | 1.00 | 4.00 | 2.0000 | 4.0000 | 4.0000 |
| P5 T8 | 9 | 0 | 5.00 | 1.00 | 6.00 | 2.0000 | 4.0000 | 4.5000 |
| P5 T9 | 9 | 0 | 3.00 | 1.00 | 4.00 | 1.5000 | 3.0000 | 4.0000 |
| P1 T1 | 9 | 0 | 3.00 | 3.00 | 6.00 | 4.5000 | 5.0000 | 6.0000 |
| P5 T10 | 9 | 0 | 3.00 | 1.00 | 4.00 | 1.6665 | 3.0000 | 4.0000 |
| P6 T1 | 9 | 0 | 3.00 | 4.00 | 7.00 | 4.0000 | 5.0000 | 6.5000 |
| P6 T2 | 9 | 0 | 4.00 | 3.00 | 7.00 | 4.0000 | 4.0000 | 5.5000 |
| P6 T3 | 9 | 0 | 3.00 | 3.00 | 6.00 | 4.0000 | 4.0000 | 5.5000 |
| P6 T4 | 9 | 0 | 2.00 | 3.00 | 5.00 | 4.0000 | 4.0000 | 5.0000 |
| P6 T5 | 9 | 0 | 1.00 | 3.00 | 4.00 | 4.0000 | 4.0000 | 4.0000 |
| P6 T6 | 9 | 0 | 4.00 | 1.00 | 5.00 | 3.0000 | 4.0000 | 4.0000 |
| P6 T7 | 9 | 0 | 3.00 | 1.00 | 4.00 | 2.0000 | 3.0000 | 4.0000 |
| P6 T8 | 9 | 0 | 4.00 | 1.00 | 5.00 | 3.0000 | 4.0000 | 4.5000 |
| P6 T9 | 9 | 0 | 3.00 | 1.00 | 4.00 | 2.0000 | 4.0000 | 4.0000 |
| P6 T10 | 9 | 0 | 3.00 | 1.00 | 4.00 | 1.6665 | 3.0000 | 4.0000 |
| P7 T1 | 8 | 1 | 3.00 | 4.00 | 7.00 | 4.2500 | 5.0000 | 6.7500 |
| P7 T2 | 8 | 1 | 3.00 | 4.00 | 7.00 | 4.0000 | 5.0000 | 6.0000 |
| P7 T3 | 8 | 1 | 2.00 | 3.00 | 5.00 | 4.0000 | 4.0000 | 4.7500 |
| P7 T4 | 8 | 1 | 3.00 | 3.00 | 6.00 | 4.0000 | 4.0000 | 5.0000 |
| P7 T5 | 8 | 1 | 4.00 | 2.00 | 6.00 | 4.0000 | 4.0000 | 4.7500 |
| P7 T6 | 8 | 1 | 5.00 | 1.00 | 6.00 | 2.5000 | 4.0000 | 4.7500 |
| P7 T7 | 8 | 1 | 4.00 | 1.00 | 5.00 | 2.2500 | 3.0000 | 5.0000 |
| P7 T8 | 8 | 1 | 5.00 | 1.00 | 6.00 | 3.0000 | 3.5000 | 4.7500 |
| P7 T9 | 8 | 1 | 4.00 | 1.00 | 5.00 | 1.2500 | 4.0000 | 4.0000 |
| P7 T10 | 8 | 1 | 3.00 | 1.00 | 4.00 | 2.0000 | 3.0000 | 3.7500 |
|  | N | |  |  |  | Percentiles | | |
|  | Valid | Missing | Range | Minimum | Maximum | 25 | 50 | 75 |
| P8 T1 | 6 | 3 | 3.00 | 4.00 | 7.00 | 4.7500 | 5.5000 | 7.0000 |
| P8 T2 | 6 | 3 | 4.00 | 3.00 | 7.00 | 3.7500 | 4.5000 | 5.5000 |
| P8 T3 | 6 | 3 | 3.00 | 3.00 | 6.00 | 3.0000 | 4.0000 | 5.2500 |
| P8 T4 | 6 | 3 | 3.00 | 3.00 | 6.00 | 3.0000 | 4.0000 | 5.2500 |
| P8 T5 | 6 | 3 | 3.00 | 2.00 | 5.00 | 2.7500 | 4.0000 | 5.0000 |
| P8 T6 | 6 | 3 | 5.00 | 1.00 | 6.00 | 2.5000 | 4.0000 | 6.0000 |
| P8 T7 | 6 | 3 | 6.00 | 1.00 | 7.00 | 1.7500 | 3.5000 | 5.5000 |
| P8 T8 | 6 | 3 | 5.00 | 1.00 | 6.00 | 1.7500 | 3.5000 | 5.2500 |
| P8 T9 | 6 | 3 | 4.00 | 1.00 | 5.00 | 1.7500 | 3.5000 | 4.2500 |
| P8 T10 | 6 | 3 | 3.00 | 1.00 | 4.00 | 2.5000 | 3.5000 | 4.0000 |
| P9 T1 | 3 | 6 | 1.00 | 5.00 | 6.00 | 5.0000 | 6.0000 | . |
| P9 T2 | 3 | 6 | 2.00 | 3.00 | 5.00 | 3.0000 | 5.0000 | . |
| P9 T3 | 3 | 6 | 3.00 | 3.00 | 6.00 | 3.0000 | 4.0000 | . |
| P9 T4 | 3 | 6 | 3.00 | 1.00 | 4.00 | 1.0000 | 4.0000 | . |
| P9 T5 | 3 | 6 | 3.00 | 2.00 | 5.00 | 2.0000 | 4.0000 | . |
| P9 T6 | 3 | 6 | 5.00 | 1.00 | 6.00 | 1.0000 | 4.0000 | . |
| P9 T7 | 3 | 6 | 5.00 | 1.00 | 6.00 | 1.0000 | 3.0000 | . |
| P9 T8 | 3 | 6 | 3.00 | 1.00 | 4.00 | 1.0000 | 2.0000 | . |
| P9 T9 | 3 | 6 | 4.00 | 1.00 | 5.00 | 1.0000 | 3.0000 | . |
| P9 T10 | 3 | 6 | 3.00 | 1.00 | 4.00 | 1.0000 | 4.0000 | . |
| P10 T1 | 3 | 6 | 2.00 | 5.00 | 7.00 | 5.0000 | 5.0000 | . |
| P10 T2 | 3 | 6 | 2.00 | 4.00 | 6.00 | 4.0000 | 5.0000 | . |
| P10 T3 | 3 | 6 | 3.00 | 3.00 | 6.00 | 3.0000 | 4.0000 | . |
| P10 T4 | 3 | 6 | 2.00 | 4.00 | 6.00 | 4.0000 | 4.0000 | . |
| P10 T5 | 3 | 6 | 3.00 | 2.00 | 5.00 | 2.0000 | 4.0000 | . |
| P10 T6 | 3 | 6 | 5.00 | 1.00 | 6.00 | 1.0000 | 3.0000 | . |
| P10 T7 | 3 | 6 | 4.00 | 1.00 | 5.00 | 1.0000 | 3.0000 | . |
| P10 T8 | 3 | 6 | 4.00 | 1.00 | 5.00 | 1.0000 | 2.0000 | . |
| P10 T9 | 3 | 6 | 4.00 | 1.00 | 5.00 | 1.0000 | 3.0000 | . |
| P10 T10 | 3 | 6 | 3.00 | 1.00 | 4.00 | 1.0000 | 3.0000 | . |

**Table 36:** Descriptive statistics for the ten median-based assessment moment variables (T1 to T10), in the Intervention group


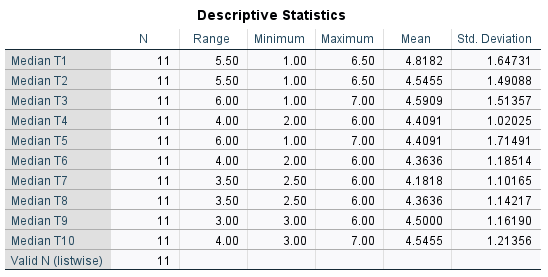


**Table 37:** Descriptive statistics for the ten median-based assessment moment variables (T1 to T10), in the Control group.

**
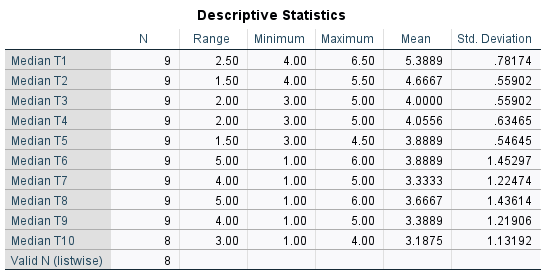
**

## Descriptives of Pretest Totals and their Spearman Correlations (N=20)

**Table 38:** Descriptives (N=20)


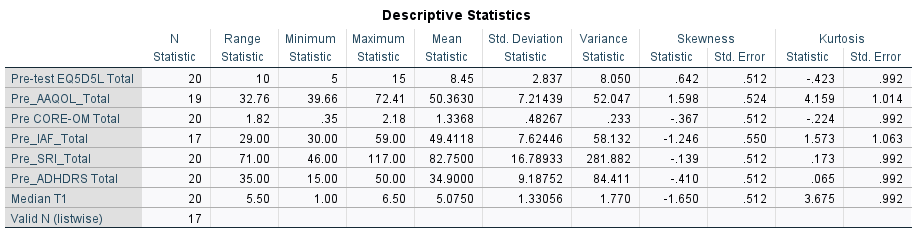


**Table 39:** Correlations Totals at pretest (N=20)

|  | | | Pre-test EQ5D5L Total | Pre_AAQoL Total | Pre CORE-OM Total | Pre_IAF  Total | Pre_SRI  Total | Pre_ADHDRS Total |
| --- | --- | --- | --- | --- | --- | --- | --- | --- |
| Spearman's rho | Pre_AAQOL Total | Correlation Coefficient | -.203 |  |  |  |  |  |
|  |  | Sig. (2-tailed) | .404 |  |  |  |  |  |
|  |  | N | 19 |  |  |  |  |  |
|  | Pre CORE-OM Total | Correlation Coefficient | .602^**^ | -.219 |  |  |  |  |
|  |  | Sig. (2-tailed) | .005 | .367 |  |  |  |  |
|  |  | N | 20 | 19 |  |  |  |  |
|  | Pre_IAF_Total | Correlation Coefficient | -.274 | .178 | -.111 |  |  |  |
|  |  | Sig. (2-tailed) | .287 | .495 | .672 |  |  |  |
|  |  | N | 17 | 17 | 17 |  |  |  |
|  | Pre_SRI_Total | Correlation Coefficient | -.117 | -.039 | -.388 | -.200 |  |  |
|  |  | Sig. (2-tailed) | .624 | .873 | .091 | .441 |  |  |
|  |  |  | Pre-test EQ5D5L Total | Pre_AAQoL Total | Pre CORE-OM Total | Pre_IAF  Total | Pre_SRI  Total | Pre_ADHDRS Total |
|  |  | N | 20 | 19 | 20 | 17 |  |  |
|  | Pre_ADHDRS Total | Correlation Coefficient | .513^*^ | -.070 | .631^**^ | -.176 | -.339 |  |
|  |  | Sig. (2-tailed) | .021 | .776 | .003 | .500 | .144 |  |
|  |  | N | 20 | 19 | 20 | 17 | 20 |  |
|  | Median T1 | Correlation Coefficient | .471^*^ | .058 | .485^*^ | -.100 | -.154 | .552^*^ |
|  |  | Sig. (2-tailed) | .036 | .815 | .030 | .704 | .516 | .012 |
|  |  | N | 20 | 19 | 20 | 17 | 20 | 20 |

| **. Correlation is significant at the 0.01 level (2-tailed). |
| --- |
| *. Correlation is significant at the 0.05 level (2-tailed). |

**Table 40:** Confidence intervals N=20

|  | Spearman's rho | Significance (2-tailed) | 95% Confidence Intervals (2-tailed)^a,b^ | |
| --- | --- | --- | --- | --- |
|  |  |  | Lower | Upper |
| Pre-test EQ5D5L Total - Pre_AAQOL_Total | -.203 | .404 | -.611 | .290 |
| Pre-test EQ5D5L Total - Pre CORE-OM Total | .602 | .005 | .205 | .829 |
| Pre-test EQ5D5L Total - Pre_IAF_Total | -.274 | .287 | -.676 | .252 |
| Pre-test EQ5D5L Total - Pre_SRI_Total | -.117 | .624 | -.542 | .356 |
| Pre-test EQ5D5L Total - Pre_ADHDRS Total | .513 | .021 | .077 | .784 |
| Pre-test EQ5D5L Total - Median T1 | .471 | .036 | .023 | .762 |
| Pre_AAQOL_Total - Pre CORE-OM Total | -.219 | .367 | -.621 | .274 |
| Pre_AAQOL_Total - Pre_IAF_Total | .178 | .495 | -.345 | .616 |
| Pre_AAQOL_Total - Pre_SRI_Total | -.039 | .873 | -.496 | .434 |
| Pre_AAQOL_Total - Pre_ADHDRS Total | -.070 | .776 | -.519 | .409 |
| Pre_AAQOL_Total - Median T1 | .058 | .815 | -.419 | .510 |
| Pre CORE-OM Total - Pre_IAF_Total | -.111 | .672 | -.572 | .404 |
| Pre CORE-OM Total - Pre_SRI_Total | -.388 | .091 | -.716 | .080 |
| Pre CORE-OM Total - Pre_ADHDRS Total | .631 | .003 | .249 | .843 |
| Pre CORE-OM Total - Median T1 | .485 | .030 | .039 | .769 |
| Pre_IAF_Total - Pre_SRI_Total | -.200 | .441 | -.631 | .324 |
| Pre_IAF_Total - Pre_ADHDRS Total | -.176 | .500 | -.615 | .347 |
| Pre_IAF_Total - Median T1 | -.100 | .704 | -.564 | .413 |
| Pre_SRI_Total - Pre_ADHDRS Total | -.339 | .144 | -.687 | .136 |
| Pre_SRI_Total - Median T1 | -.154 | .516 | -.568 | .322 |
| Pre_ADHDRS Total - Median T1 | .552 | .012 | .132 | .804 |
| a. Estimation is based on Fisher's r-to-z transformation. b. Estimation of standard error is based on the formula proposed by Fieller, Hartley, and Pearson. | | | | |

## Descriptives of Post-test Totals and their Spearman Correlations, and demographics (N=20)

**Table 41:** Correlations Totals and Demographics at post-test (N=20)

|  | | | Gender | Age years | Race Groups | Diagnosis | Date of Diagnosis | | Medication | | Months between Pre and Post-test | |  |
| --- | --- | --- | --- | --- | --- | --- | --- | --- | --- | --- | --- | --- | --- |
| Spearman's rho | Age years | Correlation Coefficient | -.267 |  |  |  |  | |  | |  | |  |
|  |  | Sig. (2-tailed) | .255 |  |  |  |  | |  | |  | |  |
|  |  | N | 20 |  |  |  |  | |  | |  | |  |
|  | Race Groups | Correlation Coefficient | .000 | .377 |  |  |  | |  | |  | |  |
|  |  | Sig. (2-tailed) | 1.000 | .101 |  |  |  | |  | |  | |  |
|  |  | N | 20 | 20 |  |  |  | |  | |  | |  |
|  | Diagnosis | Correlation Coefficient | -.035 | .009 | .042 |  |  | |  | |  | |  |
|  |  | Sig. (2-tailed) | .883 | .971 | .860 |  |  | |  | |  | |  |
|  |  | N | 20 | 20 | 20 |  |  | |  | |  | |  |
|  | Date of Diagnosis | Correlation Coefficient | .245 | -.408 | -.323 | .185 |  | |  | |  | |  |
|  |  | Sig. (2-tailed) | .298 | .074 | .165 | .434 |  | |  | |  | |  |
|  |  | N | 20 | 20 | 20 | 20 |  | |  | |  | |  |
|  | Medication | Correlation Coefficient | -.368 | .195 | .176 | -.042 | -.286 | |  | |  | |  |
|  |  | Sig. (2-tailed) | .110 | .411 | .457 | .860 | .222 | |  | |  | |  |
|  |  | N | 20 | 20 | 20 | 20 | 20 | |  | |  | |  |
|  |  |  | Gender | Age years | Race Groups | Diagnosis | Date of Diagnosis | | Medication | | Months between Pre and Post-test | |  |
|  | Months between Pre and Post-test | Correlation Coefficient | -.275 | .013 | -.013 | .118 | .111 | | -.053 | |  | |  |
|  |  | Sig. (2-tailed) | .241 | .957 | .956 | .622 | .642 | | .824 | |  | |  |
|  |  | N | 20 | 20 | 20 | 20 | 20 | | 20 | |  | |  |
|  | Post-test EQ5D5L_Total | Correlation Coefficient | .148 | .120 | -.186 | -.024 | .573^**^ | | .000 | | .069 | |  |
|  |  | Sig. (2-tailed) | .535 | .615 | .433 | .921 | .008 | | 1.000 | | .773 | |  |
|  |  | N | 20 | 20 | 20 | 20 | 20 | | 20 | | 20 | |  |
|  | Post_AAQOL_Total | Correlation Coefficient | -.132 | -.320 | -.119 | .000 | -.166 | | .436 | | -.276 | |  |
|  |  | Sig. (2-tailed) | .589 | .181 | .628 | 1.000 | .497 | | .062 | | .253 | |  |
|  |  | N | 19 | 19 | 19 | 19 | 19 | | 19 | | 19 | |  |
|  | Post CORE-OM Total | Correlation Coefficient | .282 | .249 | .049 | -.199 | .341 | | -.182 | | .070 | |  |
|  |  | Sig. (2-tailed) | .229 | .290 | .839 | .401 | .142 | | .441 | | .770 | |  |
|  |  | N | 20 | 20 | 20 | 20 | 20 | | 20 | | 20 | |  |
|  | Post_IAF  Total | Correlation Coefficient | -.366 | .323 | -.134 | .142 | -.265 | | .413 | | -.187 | |  |
|  |  | Sig. (2-tailed) | .113 | .165 | .574 | .549 | .258 | | .070 | | .430 | |  |
|  |  | N | 20 | 20 | 20 | 20 | 20 | | 20 | | 20 | |  |
|  | Post_SRI  Total | Correlation Coefficient | -.175 | .433 | .109 | .098 | -.543^*^ | | .377 | | -.054 | |  |
|  |  | Sig. (2-tailed) | .461 | .057 | .646 | .682 | .013 | | .102 | | .821 | |  |
|  |  |  | Gender | Age years | Race Groups | Diagnosis | Date of Diagnosis | | Medication | | Months between Pre and Post-test | |  |
|  |  | N | 20 | 20 | 20 | 20 | 20 | | 20 | | 20 | |  |
|  | Post_ADHDRSI Total | Correlation Coefficient | .129 | -.043 | -.012 | .031 | .553^*^ | | -.121 | | .171 | |  |
|  |  | Sig. (2-tailed) | .587 | .858 | .959 | .898 | .011 | | .610 | | .471 | |  |
|  |  | N | 20 | 20 | 20 | 20 | 20 | | 20 | | 20 | |  |
|  | Median T10 | Correlation Coefficient | -.271 | -.315 | -.309 | .126 | .145 | | .384 | | .168 | |  |
|  |  | Sig. (2-tailed) | .247 | .175 | .184 | .596 | .543 | | .095 | | .479 | |  |
|  |  | N | 20 | 20 | 20 | 20 | 20 | | 20 | | 20 | |  |
|  |  |  |  |  |  |  |  |  | |  | |  | |

**Table 42:** Correlations Totals and Demographics at post-test (N=20) - part 2

|  | | **Correlations** | | | | | | |  |  |
| --- | --- | --- | --- | --- | --- | --- | --- | --- | --- | --- |
|  | | | | | Post-test EQ5D5L Total | Post AAQOL Total | Post CORE-OM Total | Post IAF Total | Post SRI Total | Post ADHDRSI Total |
| Spearman's rho | Age years | | | Correlation Coefficient |  |  |  |  |  |  |
|  |  |  |  | Sig. (2-tailed) |  |  |  |  |  |  |
|  |  |  |  | N |  |  |  |  |  |  |
|  | Race Groups | | | Correlation Coefficient |  |  |  |  |  |  |
|  |  |  |  | Sig. (2-tailed) |  |  |  |  |  |  |
|  |  |  |  | N |  |  |  |  |  |  |
|  |  | | |  | Post-test EQ5D5L Total | Post AAQOL Total | Post CORE-OM Total | Post IAF Total | Post SRI Total | Post ADHDRSI Total |
|  | Diagnosis | | | Correlation Coefficient |  |  |  |  |  |  |
|  |  |  |  | Sig. (2-tailed) |  |  |  |  |  |  |
|  |  |  |  | N |  |  |  |  |  |  |
|  | Date of Diagnosis | | | Correlation Coefficient |  |  |  |  |  |  |
|  |  |  |  | Sig. (2-tailed) |  |  |  |  |  |  |
|  |  |  |  | N |  |  |  |  |  |  |
|  | Medication | | | Correlation Coefficient |  |  |  |  |  |  |
|  |  |  |  | Sig. (2-tailed) |  |  |  |  |  |  |
|  |  |  |  | N |  |  |  |  |  |  |
|  | Months between Pre and Post-test | | | Correlation Coefficient |  |  |  |  |  |  |
|  |  |  |  | Sig. (2-tailed) |  |  |  |  |  |  |
|  |  |  |  | N |  |  |  |  |  |  |
|  | Post-test EQ5D5L_Total | | | Correlation Coefficient |  |  |  |  |  |  |
|  |  |  |  | Sig. (2-tailed) |  |  |  |  |  |  |
|  |  |  |  | N |  |  |  |  |  |  |
|  | Post AAQOL Total | | | Correlation Coefficient | -.241 |  |  |  |  |  |
|  |  |  |  | Sig. (2-tailed) | .320 |  |  |  |  |  |
|  |  |  |  | N | 19 |  |  |  |  |  |
|  | Post CORE-OM Total | | | Correlation Coefficient | .505^*^ | -.580^**^ |  |  |  |  |
|  |  |  |  |  | Post-test EQ5D5L Total | Post AAQOL Total | Post CORE-OM Total | Post IAF Total | Post SRI Total | Post ADHDRSI Total |
|  |  |  |  | Sig. (2-tailed) | .023 | .009 |  |  |  |  |
|  |  |  |  | N | 20 | 19 |  |  |  |  |
|  | Post IAF Total | | | Correlation Coefficient | -.012 | .311 | -.186 |  |  |  |
|  |  |  |  | Sig. (2-tailed) | .962 | .196 | .432 |  |  |  |
|  |  |  |  | N | 20 | 19 | 20 |  |  |  |
|  | Post SRI Total | | | Correlation Coefficient | -.179 | .237 | -.083 | .765^**^ |  |  |
|  |  |  |  | Sig. (2-tailed) | .449 | .328 | .729 | <.001 |  |  |
|  |  |  |  | N | 20 | 19 | 20 | 20 |  |  |
|  | Post ADHDRSI Total | | | Correlation Coefficient | .272 | -.177 | .447^*^ | -.257 | -.385 |  |
|  |  |  |  | Sig. (2-tailed) | .246 | .469 | .048 | .274 | .093 |  |
|  |  |  |  | N | 20 | 19 | 20 | 20 | 20 |  |
|  | Median T10 | | | Correlation Coefficient | .137 | .430 | -.274 | -.160 | -.105 | -.154 |
|  |  |  |  | Sig. (2-tailed) | .564 | .066 | .243 | .501 | .661 | .517 |
|  |  |  |  | N | 20 | 19 | 20 | 20 | 20 | 20 |
|  | |  |  |  |  |  |  |  |  |  |

| **. Correlation is significant at the 0.01 level (2-tailed). |
| --- |
| *. Correlation is significant at the 0.05 level (2-tailed). |

# Pre and post-test comparison test results

## EQ-5D-5L: SPSS Sign Test Results

**Table 43:** Sign test results for the mobility dimension in the intervention group


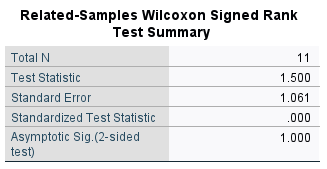


**Table 44:** Sign test results for the Self-care dimension in the intervention group


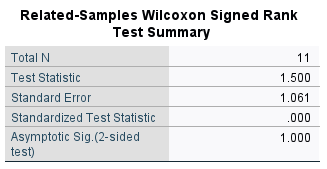


**Table 45:** Sign test results for the Activities dimension in the intervention group


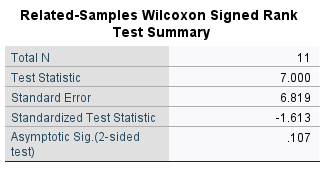


**Table 46:** Sign test results for the Pain/Discomfort dimension in the intervention group.


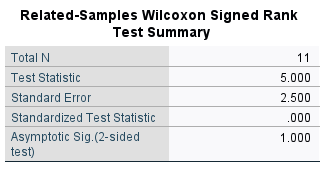


**Table 47:** Sign test results for the Anxiety/ Depression dimension in the intervention group.


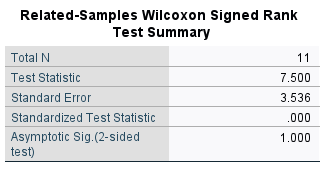


**Table 48:** Sign test results for the Total scores indicator in the intervention group.


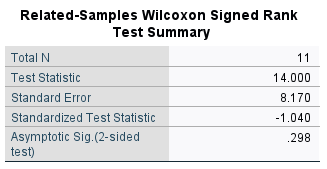


**Table 49:** Sign test results for the Index Value indicator in the intervention group


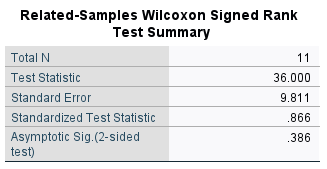


**Table 50:** Sign test results for the Mobility dimension in the control group


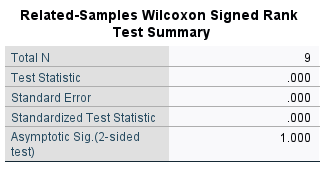


**Table 51:** Sign test results for the Self-care dimension in the control group


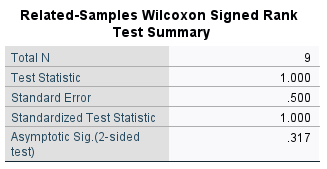


**Table 52:** Sign test results for the Activities dimension in the control group


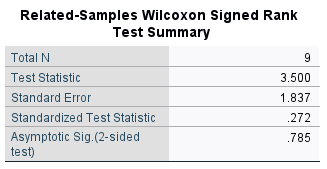


**Table 53:** Sign test results for the Pain/ Discomfort dimension in the control group


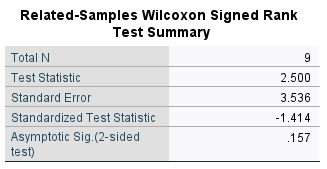


**Table 54:** Sign test results for the Anxiety/Depression dimension in the control group


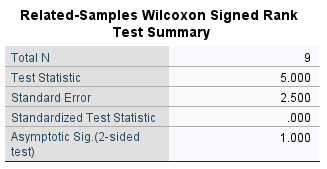


**Table 55:** Sign test results for the Total score indicator in the control group


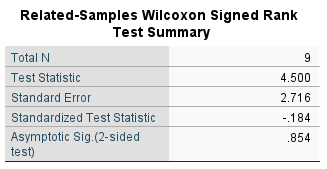


**Table 56:** Sign test results for the Index value indicator in the control group


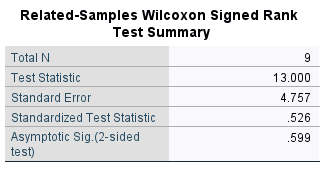


## CORE-OM: SPSS Sign Test Results

**Table 57:** Sign test results for the Wellbeing measure in the Intervention group.


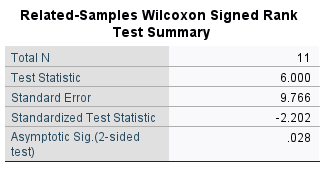


**Table 58:** Sign test results for the Problems measure in the Intervention group

**
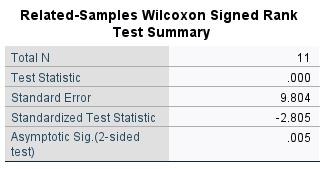
**

**Table 59:** Sign test results for the Functions measure in the Intervention group

**
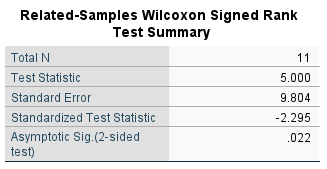
**

**Table 60:** Sign test results for the Risks measure in the Intervention group

**
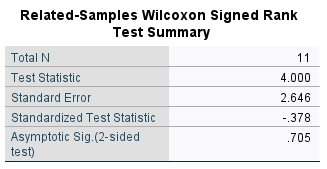
**

**Table 61:** Sign test results for the Total measure in the Intervention group

**
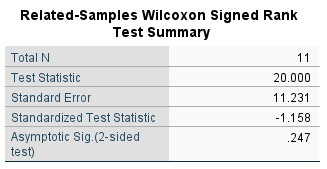
**

**Table 62:** Sign test results for the Non Risk measure in the Intervention group


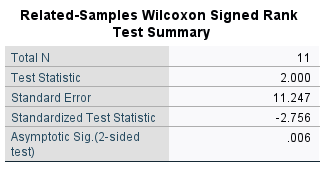


**Table 63:** Sign test results for the Risk Self measure in the Intervention group

**
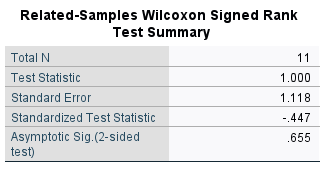
**

**Table 64:** Sign test results for the Risk Others measure in the Intervention group

**
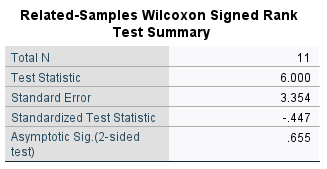
**

**Table 65:** Sign test results for the Wellbeing measure in the Control group

**
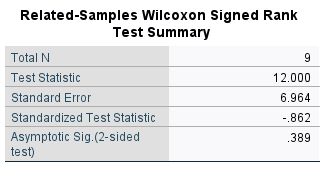
**

**Table 66:** Sign test results for the Problems measure in the Control group

**
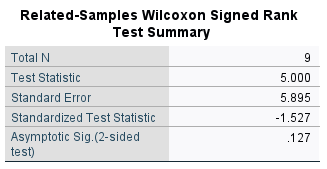
**

**Table 67:** Sign test results for the Functions measure in the Control group

**
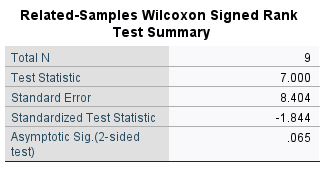
**

**Table 68:** Sign test results for the Risks measure in the Control group


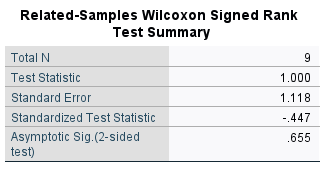


**Table 69:** Sign test results for the Total measure in the Control group

**
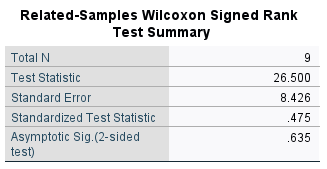
**

**Table 70:** Sign test results for the Non Risk measure in the Control group

**
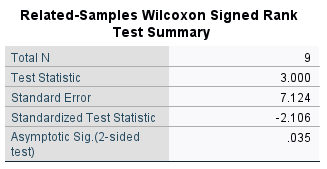
**

**Table 71:** Sign test results for the Risk Self measure in the Control group

**
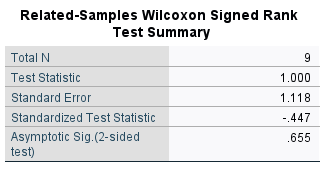
**

**Table 72:** Sign test results for the Risk Others measure in the Control group

**
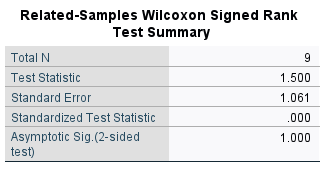
**

## ADHDRS: SPSS Sign Test Results

**Table 73:** Sign test results for the Inattention measure in the Intervention group


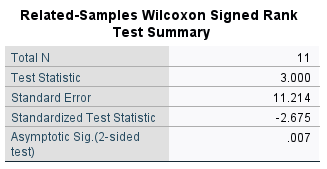


**Table 74:** Sign test results for the Hyperactivity measure in the Intervention group


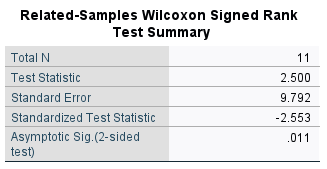


**Table 75:** Sign test results for the ADHDRS Total measure in the Intervention group


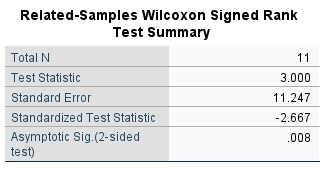


**Table 76:** Sign test results for the Inattention measure in the Control group


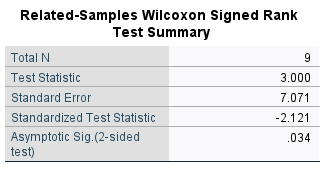


**Table 77:** Sign test results for the Hyperactivity measure in the Control group


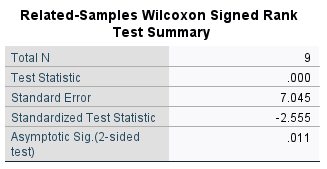


**Table 78:** Sign test results for the ADHDRS Total measure in the Control group

**
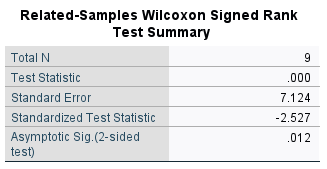
**

## AAQoL: SPSS Sign Test Results

**Table 79:** Sign test results for the Productivity scale in the Intervention group


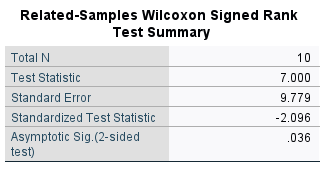


**Table 80:** Sign test results for the Mental Health scale in the Intervention group


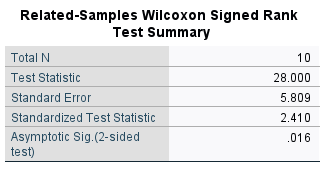


**Table 81:** Sign test results for the Outlook scale in the Intervention group


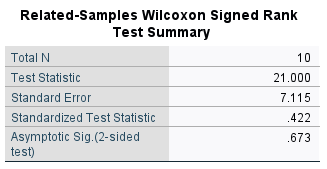


**Table 82:** Sign test results for the Relationships scale in the Intervention group
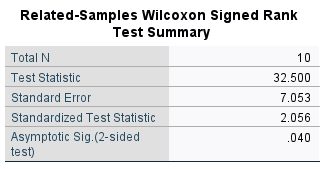


**Table 83:** Sign test results for the Total scale in the Intervention group
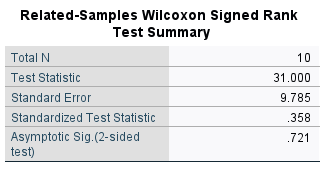


**Table 84:** Sign test results for the Productivity scale in the Control group


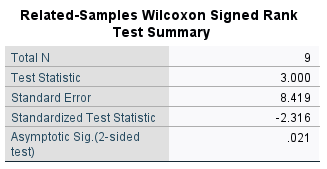


**Table 85:** Sign test results for the Mental Health scale in the Control group


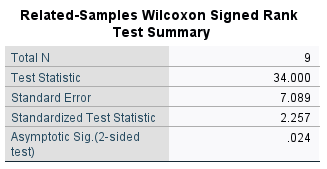


**Table 86:** Sign test results for the Outlook scale in the Control group


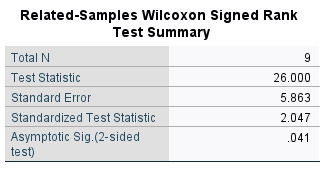


**Table 87:** Sign test results for the Relationships scale in the Control group


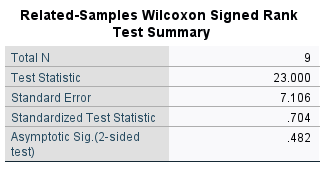


**Table 88:** Sign test results for the Total scale in the Control group


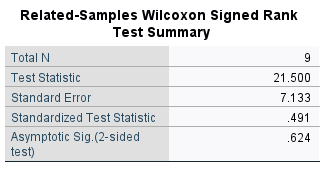


## SRI: SPSS Sign Test Results

**Table 89:** Sign test results for the Self-Reflection measure in the Intervention group


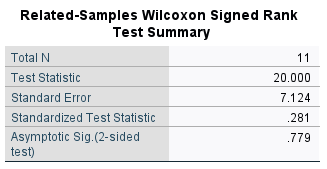


**Table 90:** Sign test results for the Need Reflection measure in the Intervention group

**
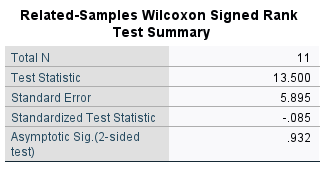
**

**Table 91:** Sign test results for the Insight measure in the Intervention group


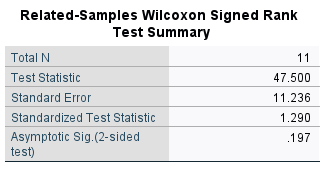


**Table 92:** Sign test results for the Total measure in the Intervention group


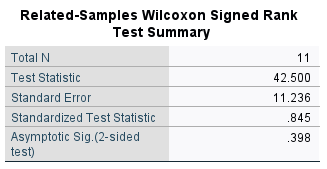


**Table 93:** Sign test results for the Self-Reflection measure in the Control group

**
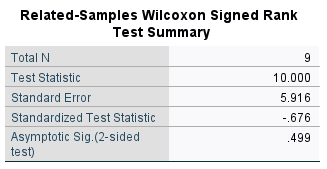
**

**Table 94:** Sign test results for the Need Reflection measure in the Control group

**
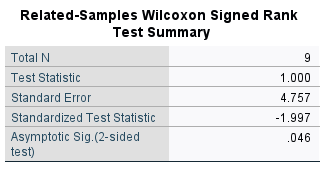
**

**Table 95:** Sign test results for the Insight measure in the Control group


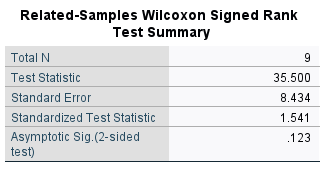


**Table 96:** Sign test results for the Total measure in the Control group

**
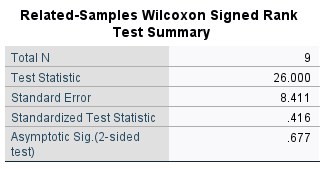
**

## IAF: SPSS Sign Test Results

**Table 97:** Sign test results for the Authorship measure in the Intervention group.


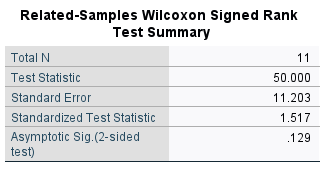


**Table 98:** Sign test results for the Control measure in the Intervention group


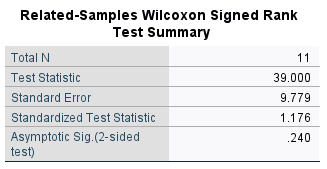


**Table 99:** Sign test results for the Interest measure in the Intervention group


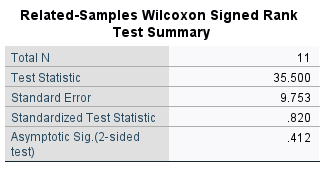


**Table 100:** Sign test results for the Total measure in the Intervention group

**
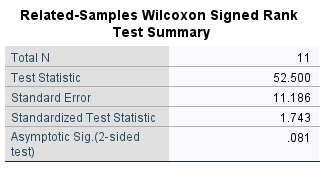
**

**Table 101:** Sign test results for the Authorship measure in the Control group
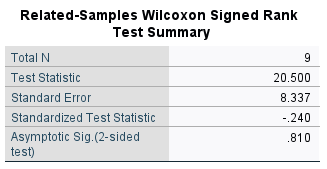


**Table 102:** Sign test results for the Control measure in the Control group


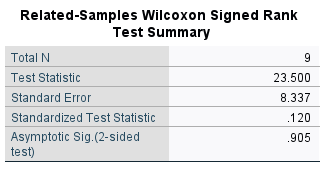


**Table 103:** Sign test results for the Interest measure in the Control group


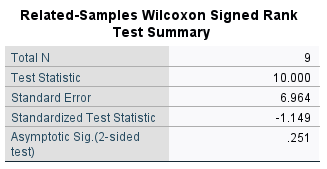


**Table 104:** Sign test results for the Total measure in the Control group


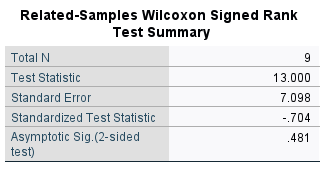


## PQ: Friedman’s test for Items

**Table 105:** Friedman’s test results for P1T1 to P1T10, in the Intervention group


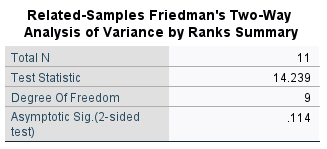


**Table 106:** Friedman’s test results for P2T1 to P2T10, in the Intervention group

**
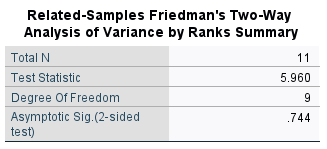
**

**Table 107:** Friedman’s test results for P3T1 to P3T10, in the Intervention group


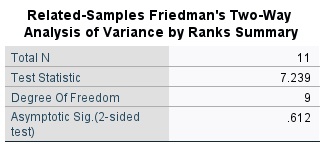


**Table 108:** Comparison of P4T1 to P4T10 in the Intervention group
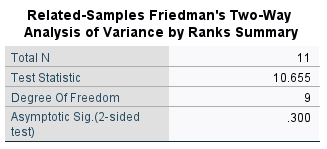


**Table 109:** Comparison of P5T1 to P5T10

**Table 110:** Comparison of P6T1 to P6T10 in the Intervention group

**Table 111:** Comparison of P7T1 to P7T10 in the Intervention group

**Table 112:** Comparison of P8T1 to P8T10 in the Intervention group

**Table 113:** Comparison of P9T1 to P9T10 in the Intervention group

**Table 114:** Comparison of P10T1 to P10T10 in the Intervention group

**Table 115:** Comparison of P1T1 to P1T10 in the Control group

**Table 116:** Multiple comparisons of P1T1 to P1T10 in the Control group

| **Sample 1-Sample 2** | **Test Statistic** | **Std. Error** | **Std. Test Statistic** | **Sig.** | **Adj. Sig.^a^** |
| --- | --- | --- | --- | --- | --- |
| P1 T10-P1 T9 | 1.111 | 1.427 | .778 | .436 | 1.000 |
| P1 T10-P1 T7 | 1.500 | 1.427 | 1.051 | .293 | 1.000 |
| P1 T10-P1 T8 | 2.000 | 1.427 | 1.401 | .161 | 1.000 |
| P1 T10-P1 T6 | 2.444 | 1.427 | 1.713 | .087 | 1.000 |
| P1 T10-P1 T5 | 2.556 | 1.427 | 1.791 | .073 | 1.000 |
| P1 T10-P1 T2 | 3.722 | 1.427 | 2.608 | .009 | .410 |
| P1 T10-P1 T3 | 3.778 | 1.427 | 2.647 | .008 | .366 |
| P1 T10-P1 T4 | 4.222 | 1.427 | 2.958 | .003 | .139 |
| P1 T10-P1 T1 | 5.333 | 1.427 | 3.737 | <.001 | .008 |
| P1 T9-P1 T7 | .389 | 1.427 | .272 | .785 | 1.000 |
| P1 T9-P1 T8 | .889 | 1.427 | .623 | .533 | 1.000 |
| P1 T9-P1 T6 | 1.333 | 1.427 | .934 | .350 | 1.000 |
| P1 T9-P1 T5 | 1.444 | 1.427 | 1.012 | .312 | 1.000 |
| P1 T9-P1 T2 | 2.611 | 1.427 | 1.829 | .067 | 1.000 |
| P1 T9-P1 T3 | 2.667 | 1.427 | 1.868 | .062 | 1.000 |
| P1 T9-P1 T4 | 3.111 | 1.427 | 2.180 | .029 | 1.000 |
| P1 T9-P1 T1 | 4.222 | 1.427 | 2.958 | .003 | .139 |
| P1 T7-P1 T8 | -.500 | 1.427 | -.350 | .726 | 1.000 |
| P1 T7-P1 T6 | .944 | 1.427 | .662 | .508 | 1.000 |
| P1 T7-P1 T5 | 1.056 | 1.427 | .740 | .460 | 1.000 |
| P1 T7-P1 T2 | 2.222 | 1.427 | 1.557 | .119 | 1.000 |
| P1 T7-P1 T3 | 2.278 | 1.427 | 1.596 | .111 | 1.000 |
| **Sample 1-Sample 2** | **Test Statistic** | **Std. Error** | **Std. Test Statistic** | **Sig.** | **Adj. Sig.^a^** |
| P1 T7-P1 T4 | 2.722 | 1.427 | 1.907 | .056 | 1.000 |
| P1 T7-P1 T1 | 3.833 | 1.427 | 2.686 | .007 | .326 |
| P1 T8-P1 T6 | .444 | 1.427 | .311 | .755 | 1.000 |
| P1 T8-P1 T5 | .556 | 1.427 | .389 | .697 | 1.000 |
| P1 T8-P1 T2 | 1.722 | 1.427 | 1.207 | .228 | 1.000 |
| P1 T8-P1 T3 | 1.778 | 1.427 | 1.246 | .213 | 1.000 |
| P1 T8-P1 T4 | 2.222 | 1.427 | 1.557 | .119 | 1.000 |
| P1 T8-P1 T1 | 3.333 | 1.427 | 2.335 | .020 | .878 |
| P1 T6-P1 T5 | .111 | 1.427 | .078 | .938 | 1.000 |
| P1 T6-P1 T2 | 1.278 | 1.427 | .895 | .371 | 1.000 |
| P1 T6-P1 T3 | 1.333 | 1.427 | .934 | .350 | 1.000 |
| P1 T6-P1 T4 | 1.778 | 1.427 | 1.246 | .213 | 1.000 |
| P1 T6-P1 T1 | 2.889 | 1.427 | 2.024 | .043 | 1.000 |
| P1 T5-P1 T2 | 1.167 | 1.427 | .817 | .414 | 1.000 |
| P1 T5-P1 T3 | 1.222 | 1.427 | .856 | .392 | 1.000 |
| P1 T5-P1 T4 | 1.667 | 1.427 | 1.168 | .243 | 1.000 |
| P1 T5-P1 T1 | 2.778 | 1.427 | 1.946 | .052 | 1.000 |
| P1 T2-P1 T3 | -.056 | 1.427 | -.039 | .969 | 1.000 |
| P1 T2-P1 T4 | -.500 | 1.427 | -.350 | .726 | 1.000 |
| P1 T2-P1 T1 | 1.611 | 1.427 | 1.129 | .259 | 1.000 |
| P1 T3-P1 T4 | -.444 | 1.427 | -.311 | .755 | 1.000 |
| P1 T3-P1 T1 | 1.556 | 1.427 | 1.090 | .276 | 1.000 |
| P1 T4-P1 T1 | 1.111 | 1.427 | .778 | .436 | 1.000 |
| Each row tests the null hypothesis that the Sample 1 and Sample 2 distributions are the same.  Asymptotic significances (2-sided tests) are displayed. The significance level is .050.  a. Significance values have been adjusted by the Bonferroni correction for multiple tests. | | | | | |

**Table 117:** Comparison of P2T1 to P1T10 in the Control group

**Table 118:** Multiple comparisons of P2T1 to P2T10 in the Control group

| Pairwise Comparisons | | | | | |
| --- | --- | --- | --- | --- | --- |
| Sample 1-Sample 2 | Test Statistic | Std. Error | Std. Test Statistic | Sig. | Adj. Sig.^a^ |
| P2 T10-P2 T9 | .722 | 1.427 | .506 | .613 | 1.000 |
| P2 T10-P2 T6 | 2.333 | 1.427 | 1.635 | .102 | 1.000 |
| P2 T10-P2 T5 | 2.444 | 1.427 | 1.713 | .087 | 1.000 |
| P2 T10-P2 T7 | 2.556 | 1.427 | 1.791 | .073 | 1.000 |
| P2 T10-P2 T3 | 2.889 | 1.427 | 2.024 | .043 | 1.000 |
| P2 T10-P2 T8 | 3.111 | 1.427 | 2.180 | .029 | 1.000 |
| P2 T10-P2 T4 | 3.944 | 1.427 | 2.764 | .006 | .257 |
| P2 T10-P2 T2 | 4.500 | 1.427 | 3.153 | .002 | .073 |
| P2 T10-P2 T1 | 5.833 | 1.427 | 4.087 | <.001 | .002 |
| P2 T9-P2 T6 | 1.611 | 1.427 | 1.129 | .259 | 1.000 |
| P2 T9-P2 T5 | 1.722 | 1.427 | 1.207 | .228 | 1.000 |
| P2 T9-P2 T7 | 1.833 | 1.427 | 1.285 | .199 | 1.000 |
| P2 T9-P2 T3 | 2.167 | 1.427 | 1.518 | .129 | 1.000 |
| P2 T9-P2 T8 | 2.389 | 1.427 | 1.674 | .094 | 1.000 |
| P2 T9-P2 T4 | 3.222 | 1.427 | 2.258 | .024 | 1.000 |
| P2 T9-P2 T2 | 3.778 | 1.427 | 2.647 | .008 | .366 |
| P2 T9-P2 T1 | 5.111 | 1.427 | 3.581 | <.001 | .015 |
| P2 T6-P2 T5 | .111 | 1.427 | .078 | .938 | 1.000 |
| P2 T6-P2 T7 | -.222 | 1.427 | -.156 | .876 | 1.000 |
| P2 T6-P2 T3 | .556 | 1.427 | .389 | .697 | 1.000 |
| Sample 1-Sample 2 | Test Statistic | Std. Error | Std. Test Statistic | Sig. | Adj. Sig.^a^ |
| P2 T6-P2 T8 | -.778 | 1.427 | -.545 | .586 | 1.000 |
| P2 T6-P2 T4 | 1.611 | 1.427 | 1.129 | .259 | 1.000 |
| P2 T6-P2 T2 | 2.167 | 1.427 | 1.518 | .129 | 1.000 |
| P2 T6-P2 T1 | 3.500 | 1.427 | 2.452 | .014 | .639 |
| P2 T5-P2 T7 | -.111 | 1.427 | -.078 | .938 | 1.000 |
| P2 T5-P2 T3 | .444 | 1.427 | .311 | .755 | 1.000 |
| P2 T5-P2 T8 | -.667 | 1.427 | -.467 | .640 | 1.000 |
| P2 T5-P2 T4 | 1.500 | 1.427 | 1.051 | .293 | 1.000 |
| P2 T5-P2 T2 | 2.056 | 1.427 | 1.440 | .150 | 1.000 |
| P2 T5-P2 T1 | 3.389 | 1.427 | 2.374 | .018 | .791 |
| P2 T7-P2 T3 | .333 | 1.427 | .234 | .815 | 1.000 |
| P2 T7-P2 T8 | -.556 | 1.427 | -.389 | .697 | 1.000 |
| P2 T7-P2 T4 | 1.389 | 1.427 | .973 | .330 | 1.000 |
| P2 T7-P2 T2 | 1.944 | 1.427 | 1.362 | .173 | 1.000 |
| P2 T7-P2 T1 | 3.278 | 1.427 | 2.297 | .022 | .974 |
| P2 T3-P2 T8 | -.222 | 1.427 | -.156 | .876 | 1.000 |
| P2 T3-P2 T4 | -1.056 | 1.427 | -.740 | .460 | 1.000 |
| P2 T3-P2 T2 | 1.611 | 1.427 | 1.129 | .259 | 1.000 |
| P2 T3-P2 T1 | 2.944 | 1.427 | 2.063 | .039 | 1.000 |
| P2 T8-P2 T4 | .833 | 1.427 | .584 | .559 | 1.000 |
| P2 T8-P2 T2 | 1.389 | 1.427 | .973 | .330 | 1.000 |
| P2 T8-P2 T1 | 2.722 | 1.427 | 1.907 | .056 | 1.000 |
| P2 T4-P2 T2 | .556 | 1.427 | .389 | .697 | 1.000 |
| P2 T4-P2 T1 | 1.889 | 1.427 | 1.323 | .186 | 1.000 |
| P2 T2-P2 T1 | 1.333 | 1.427 | .934 | .350 | 1.000 |
| Each row tests the null hypothesis that the Sample 1 and Sample 2 distributions are the same.  Asymptotic significances (2-sided tests) are displayed. The significance level is .050.  a. Significance values have been adjusted by the Bonferroni correction for multiple tests. | | | | | |

**Table 119:** Comparison of P3T1 to P3T10 in the Control group

**Table 120:** Multiple comparisons of P3T1 to P3T10 in the Control group

| Sample 1-Sample 2 | Test Statistic | Std. Error | Std. Test Statistic | Sig. | Adj. Sig.^a^ |
| --- | --- | --- | --- | --- | --- |
| P3 T10-P3 T9 | .222 | 1.427 | .156 | .876 | 1.000 |
| P3 T10-P3 T5 | .778 | 1.427 | .545 | .586 | 1.000 |
| P3 T10-P3 T8 | .778 | 1.427 | .545 | .586 | 1.000 |
| P3 T10-P3 T6 | 1.056 | 1.427 | .740 | .460 | 1.000 |
| P3 T10-P3 T7 | 1.222 | 1.427 | .856 | .392 | 1.000 |
| P3 T10-P3 T4 | 2.111 | 1.427 | 1.479 | .139 | 1.000 |
| P3 T10-P3 T3 | 2.556 | 1.427 | 1.791 | .073 | 1.000 |
| P3 T10-P3 T2 | 3.222 | 1.427 | 2.258 | .024 | 1.000 |
| P3 T10-P3 T1 | 4.722 | 1.427 | 3.309 | <.001 | .042 |
| P3 T9-P3 T8 | .556 | 1.427 | .389 | .697 | 1.000 |
| P3 T9-P3 T5 | .556 | 1.427 | .389 | .697 | 1.000 |
| P3 T9-P3 T6 | .833 | 1.427 | .584 | .559 | 1.000 |
| P3 T9-P3 T7 | 1.000 | 1.427 | .701 | .484 | 1.000 |
| P3 T9-P3 T4 | 1.889 | 1.427 | 1.323 | .186 | 1.000 |
| P3 T9-P3 T3 | 2.333 | 1.427 | 1.635 | .102 | 1.000 |
| P3 T9-P3 T2 | 3.000 | 1.427 | 2.102 | .036 | 1.000 |
| P3 T9-P3 T1 | 4.500 | 1.427 | 3.153 | .002 | .073 |
| P3 T8-P3 T1 | 3.944 | 1.427 | 2.764 | .006 | .257 |
| P3 T5-P3 T2 | 2.444 | 1.427 | 1.713 | .087 | 1.000 |
| P3 T8-P3 T2 | 2.444 | 1.427 | 1.713 | .087 | 1.000 |
| P3 T5-P3 T3 | 1.778 | 1.427 | 1.246 | .213 | 1.000 |
| P3 T8-P3 T4 | 1.333 | 1.427 | .934 | .350 | 1.000 |
| Sample 1-Sample 2 | Test Statistic | Std. Error | Std. Test Statistic | Sig. | Adj. Sig.^a^ |
| P3 T8-P3 T3 | 1.778 | 1.427 | 1.246 | .213 | 1.000 |
| P3 T5-P3 T8 | .000 | 1.427 | .000 | 1.000 | 1.000 |
| P3 T5-P3 T6 | -.278 | 1.427 | -.195 | .846 | 1.000 |
| P3 T5-P3 T7 | -.444 | 1.427 | -.311 | .755 | 1.000 |
| P3 T5-P3 T4 | 1.333 | 1.427 | .934 | .350 | 1.000 |
| P3 T8-P3 T6 | .278 | 1.427 | .195 | .846 | 1.000 |
| P3 T5-P3 T1 | 3.944 | 1.427 | 2.764 | .006 | .257 |
| P3 T8-P3 T7 | .444 | 1.427 | .311 | .755 | 1.000 |
| P3 T6-P3 T7 | -.167 | 1.427 | -.117 | .907 | 1.000 |
| P3 T6-P3 T4 | 1.056 | 1.427 | .740 | .460 | 1.000 |
| P3 T6-P3 T3 | 1.500 | 1.427 | 1.051 | .293 | 1.000 |
| P3 T6-P3 T2 | 2.167 | 1.427 | 1.518 | .129 | 1.000 |
| P3 T6-P3 T1 | 3.667 | 1.427 | 2.569 | .010 | .459 |
| P3 T7-P3 T4 | .889 | 1.427 | .623 | .533 | 1.000 |
| P3 T7-P3 T3 | 1.333 | 1.427 | .934 | .350 | 1.000 |
| P3 T7-P3 T2 | 2.000 | 1.427 | 1.401 | .161 | 1.000 |
| P3 T7-P3 T1 | 3.500 | 1.427 | 2.452 | .014 | .639 |
| P3 T4-P3 T3 | .444 | 1.427 | .311 | .755 | 1.000 |
| P3 T4-P3 T2 | 1.111 | 1.427 | .778 | .436 | 1.000 |
| P3 T4-P3 T1 | 2.611 | 1.427 | 1.829 | .067 | 1.000 |
| P3 T3-P3 T2 | .667 | 1.427 | .467 | .640 | 1.000 |
| P3 T3-P3 T1 | 2.167 | 1.427 | 1.518 | .129 | 1.000 |
| P3 T2-P3 T1 | 1.500 | 1.427 | 1.051 | .293 | 1.000 |
| Each row tests the null hypothesis that the Sample 1 and Sample 2 distributions are the same.  Asymptotic significances (2-sided tests) are displayed. The significance level is .050. | | | | | |
| a. Significance values have been adjusted by the Bonferroni correction for multiple tests. | | | | | |

**Table 121:** Comparison of P4T1 to P4T10 in the Control group

**Table 122:** Multiple comparisons of P4T1 to P4T10 in the Control group

| Sample 1-Sample 2 | Test Statistic | Std. Error | Std. Test Statistic | Sig. | Adj. Sig.^a^ |
| --- | --- | --- | --- | --- | --- |
| P4 T10-P4 T9 | 1.333 | 1.427 | .934 | .350 | 1.000 |
| P4 T10-P4 T8 | 1.389 | 1.427 | .973 | .330 | 1.000 |
| P4 T10-P4 T7 | 2.056 | 1.427 | 1.440 | .150 | 1.000 |
| P4 T10-P4 T6 | 2.722 | 1.427 | 1.907 | .056 | 1.000 |
| P4 T10-P4 T4 | 3.056 | 1.427 | 2.141 | .032 | 1.000 |
| P4 T10-P4 T5 | 3.056 | 1.427 | 2.141 | .032 | 1.000 |
| P4 T10-P4 T3 | 3.389 | 1.427 | 2.374 | .018 | .791 |
| P4 T10-P4 T1 | 5.111 | 1.427 | 3.581 | <.001 | .015 |
| P4 T10-P4 T2 | 5.111 | 1.427 | 3.581 | <.001 | .015 |
| P4 T9-P4 T8 | .056 | 1.427 | .039 | .969 | 1.000 |
| P4 T9-P4 T7 | .722 | 1.427 | .506 | .613 | 1.000 |
| P4 T9-P4 T6 | 1.389 | 1.427 | .973 | .330 | 1.000 |
| P4 T9-P4 T4 | 1.722 | 1.427 | 1.207 | .228 | 1.000 |
| P4 T9-P4 T5 | 1.722 | 1.427 | 1.207 | .228 | 1.000 |
| P4 T9-P4 T3 | 2.056 | 1.427 | 1.440 | .150 | 1.000 |
| P4 T9-P4 T1 | 3.778 | 1.427 | 2.647 | .008 | .366 |
| P4 T9-P4 T2 | 3.778 | 1.427 | 2.647 | .008 | .366 |
| P4 T8-P4 T7 | .667 | 1.427 | .467 | .640 | 1.000 |
| P4 T8-P4 T6 | 1.333 | 1.427 | .934 | .350 | 1.000 |
| P4 T8-P4 T4 | 1.667 | 1.427 | 1.168 | .243 | 1.000 |
| P4 T8-P4 T5 | 1.667 | 1.427 | 1.168 | .243 | 1.000 |
| Sample 1-Sample 2 | Test Statistic | Std. Error | Std. Test Statistic | Sig. | Adj. Sig.^a^ |
| P4 T8-P4 T3 | 2.000 | 1.427 | 1.401 | .161 | 1.000 |
| P4 T8-P4 T1 | 3.722 | 1.427 | 2.608 | .009 | .410 |
| P4 T8-P4 T2 | 3.722 | 1.427 | 2.608 | .009 | .410 |
| P4 T7-P4 T6 | .667 | 1.427 | .467 | .640 | 1.000 |
| P4 T7-P4 T4 | 1.000 | 1.427 | .701 | .484 | 1.000 |
| P4 T7-P4 T5 | 1.000 | 1.427 | .701 | .484 | 1.000 |
| P4 T7-P4 T3 | 1.333 | 1.427 | .934 | .350 | 1.000 |
| P4 T7-P4 T1 | 3.056 | 1.427 | 2.141 | .032 | 1.000 |
| P4 T7-P4 T2 | 3.056 | 1.427 | 2.141 | .032 | 1.000 |
| P4 T6-P4 T5 | .333 | 1.427 | .234 | .815 | 1.000 |
| P4 T6-P4 T4 | .333 | 1.427 | .234 | .815 | 1.000 |
| P4 T6-P4 T3 | .667 | 1.427 | .467 | .640 | 1.000 |
| P4 T6-P4 T2 | 2.389 | 1.427 | 1.674 | .094 | 1.000 |
| P4 T6-P4 T1 | 2.389 | 1.427 | 1.674 | .094 | 1.000 |
| P4 T4-P4 T3 | .333 | 1.427 | .234 | .815 | 1.000 |
| P4 T5-P4 T3 | .333 | 1.427 | .234 | .815 | 1.000 |
| P4 T4-P4 T5 | .000 | 1.427 | .000 | 1.000 | 1.000 |
| P4 T5-P4 T2 | 2.056 | 1.427 | 1.440 | .150 | 1.000 |
| P4 T4-P4 T2 | 2.056 | 1.427 | 1.440 | .150 | 1.000 |
| P4 T5-P4 T1 | 2.056 | 1.427 | 1.440 | .150 | 1.000 |
| P4 T4-P4 T1 | 2.056 | 1.427 | 1.440 | .150 | 1.000 |
| P4 T3-P4 T1 | 1.722 | 1.427 | 1.207 | .228 | 1.000 |
| P4 T3-P4 T2 | 1.722 | 1.427 | 1.207 | .228 | 1.000 |
| P4 T1-P4 T2 | .000 | 1.427 | .000 | 1.000 | 1.000 |
| Each row tests the null hypothesis that the Sample 1 and Sample 2 distributions are the same.  Asymptotic significances (2-sided tests) are displayed. The significance level is .050. | | | | | |
| a. Significance values have been adjusted by the Bonferroni correction for multiple tests. | | | | | |

**Table 123:** Comparison of P5T1 to P5T10 in the Control group

**Table 124:** Multiple comparisons of P5T1 to P5T10 in the Control group

| Sample 1-Sample 2 | Test Statistic | Std. Error | Std. Test Statistic | Sig. | Adj. Sig.^a^ |
| --- | --- | --- | --- | --- | --- |
| P5 T9-P5 T10 | -.278 | 1.427 | -.195 | .846 | 1.000 |
| P5 T9-P5 T4 | .611 | 1.427 | .428 | .669 | 1.000 |
| P5 T9-P5 T7 | 1.389 | 1.427 | .973 | .330 | 1.000 |
| P5 T9-P5 T6 | 1.778 | 1.427 | 1.246 | .213 | 1.000 |
| P5 T9-P5 T8 | 1.889 | 1.427 | 1.323 | .186 | 1.000 |
| P5 T9-P5 T3 | 2.500 | 1.427 | 1.752 | .080 | 1.000 |
| P5 T9-P5 T5 | 2.889 | 1.427 | 2.024 | .043 | 1.000 |
| P5 T9-P5 T2 | 4.278 | 1.427 | 2.997 | .003 | .123 |
| P5 T9-P5 T1 | 6.056 | 1.427 | 4.243 | <.001 | .001 |
| P5 T10-P5 T4 | .333 | 1.427 | .234 | .815 | 1.000 |
| P5 T10-P5 T7 | 1.111 | 1.427 | .778 | .436 | 1.000 |
| P5 T10-P5 T6 | 1.500 | 1.427 | 1.051 | .293 | 1.000 |
| P5 T10-P5 T8 | 1.611 | 1.427 | 1.129 | .259 | 1.000 |
| P5 T10-P5 T3 | 2.222 | 1.427 | 1.557 | .119 | 1.000 |
| P5 T10-P5 T5 | 2.611 | 1.427 | 1.829 | .067 | 1.000 |
| P5 T10-P5 T2 | 4.000 | 1.427 | 2.803 | .005 | .228 |
| P5 T10-P5 T1 | 5.778 | 1.427 | 4.048 | <.001 | .002 |
| P5 T4-P5 T7 | -.778 | 1.427 | -.545 | .586 | 1.000 |
| P5 T4-P5 T6 | -1.167 | 1.427 | -.817 | .414 | 1.000 |
| P5 T4-P5 T8 | -1.278 | 1.427 | -.895 | .371 | 1.000 |
| P5 T4-P5 T3 | 1.889 | 1.427 | 1.323 | .186 | 1.000 |
| P5 T4-P5 T5 | -2.278 | 1.427 | -1.596 | .111 | 1.000 |
| Sample 1-Sample 2 | Test Statistic | Std. Error | Std. Test Statistic | Sig. | Adj. Sig.^a^ |
| P5 T4-P5 T2 | 3.667 | 1.427 | 2.569 | .010 | .459 |
| P5 T4-P5 T1 | 5.444 | 1.427 | 3.815 | <.001 | .006 |
| P5 T7-P5 T6 | .389 | 1.427 | .272 | .785 | 1.000 |
| P5 T7-P5 T8 | -.500 | 1.427 | -.350 | .726 | 1.000 |
| P5 T7-P5 T3 | 1.111 | 1.427 | .778 | .436 | 1.000 |
| P5 T7-P5 T5 | 1.500 | 1.427 | 1.051 | .293 | 1.000 |
| P5 T7-P5 T2 | 2.889 | 1.427 | 2.024 | .043 | 1.000 |
| P5 T7-P5 T1 | 4.667 | 1.427 | 3.270 | .001 | .048 |
| P5 T6-P5 T8 | -.111 | 1.427 | -.078 | .938 | 1.000 |
| P5 T6-P5 T3 | .722 | 1.427 | .506 | .613 | 1.000 |
| P5 T6-P5 T5 | 1.111 | 1.427 | .778 | .436 | 1.000 |
| P5 T6-P5 T2 | 2.500 | 1.427 | 1.752 | .080 | 1.000 |
| P5 T6-P5 T1 | 4.278 | 1.427 | 2.997 | .003 | .123 |
| P5 T8-P5 T3 | .611 | 1.427 | .428 | .669 | 1.000 |
| P5 T8-P5 T5 | 1.000 | 1.427 | .701 | .484 | 1.000 |
| P5 T8-P5 T2 | 2.389 | 1.427 | 1.674 | .094 | 1.000 |
| P5 T8-P5 T1 | 4.167 | 1.427 | 2.919 | .004 | .158 |
| P5 T3-P5 T5 | -.389 | 1.427 | -.272 | .785 | 1.000 |
| P5 T3-P5 T2 | 1.778 | 1.427 | 1.246 | .213 | 1.000 |
| P5 T3-P5 T1 | 3.556 | 1.427 | 2.491 | .013 | .573 |
| P5 T5-P5 T2 | 1.389 | 1.427 | .973 | .330 | 1.000 |
| P5 T5-P5 T1 | 3.167 | 1.427 | 2.219 | .027 | 1.000 |
| P5 T2-P5 T1 | 1.778 | 1.427 | 1.246 | .213 | 1.000 |
| Each row tests the null hypothesis that the Sample 1 and Sample 2 distributions are the same.  Asymptotic significances (2-sided tests) are displayed. The significance level is .050. | | | | | |
| a. Significance values have been adjusted by the Bonferroni correction for multiple tests. | | | | | |

**Table 125:** Comparison of P6 T1 to T10 in the Control group

**Table 126:** Multiple comparisons of P6T1 to P6T10 in the Control group

| Sample 1-Sample 2 | Test Statistic | Std. Error | Std. Test Statistic | Sig. | Adj. Sig.^a^ |
| --- | --- | --- | --- | --- | --- |
| P6 T10-P6 T7 | .222 | 1.427 | .156 | .876 | 1.000 |
| P6 T10-P6 T9 | .333 | 1.427 | .234 | .815 | 1.000 |
| P6 T10-P6 T8 | 1.556 | 1.427 | 1.090 | .276 | 1.000 |
| P6 T10-P6 T6 | 1.778 | 1.427 | 1.246 | .213 | 1.000 |
| P6 T10-P6 T5 | 2.333 | 1.427 | 1.635 | .102 | 1.000 |
| P6 T10-P6 T4 | 3.167 | 1.427 | 2.219 | .027 | 1.000 |
| P6 T10-P6 T3 | 3.778 | 1.427 | 2.647 | .008 | .366 |
| P6 T10-P6 T2 | 3.833 | 1.427 | 2.686 | .007 | .326 |
| P6 T10-P6 T1 | 5.222 | 1.427 | 3.659 | <.001 | .011 |
| P6 T7-P6 T9 | -.111 | 1.427 | -.078 | .938 | 1.000 |
| P6 T7-P6 T8 | -1.333 | 1.427 | -.934 | .350 | 1.000 |
| P6 T7-P6 T6 | 1.556 | 1.427 | 1.090 | .276 | 1.000 |
| P6 T7-P6 T5 | 2.111 | 1.427 | 1.479 | .139 | 1.000 |
| P6 T7-P6 T4 | 2.944 | 1.427 | 2.063 | .039 | 1.000 |
| P6 T7-P6 T3 | 3.556 | 1.427 | 2.491 | .013 | .573 |
| P6 T7-P6 T2 | 3.611 | 1.427 | 2.530 | .011 | .513 |
| P6 T7-P6 T1 | 5.000 | 1.427 | 3.503 | <.001 | .021 |
| P6 T9-P6 T8 | 1.222 | 1.427 | .856 | .392 | 1.000 |
| P6 T9-P6 T6 | 1.444 | 1.427 | 1.012 | .312 | 1.000 |
| P6 T9-P6 T5 | 2.000 | 1.427 | 1.401 | .161 | 1.000 |
| P6 T9-P6 T4 | 2.833 | 1.427 | 1.985 | .047 | 1.000 |
| P6 T9-P6 T3 | 3.444 | 1.427 | 2.413 | .016 | .711 |
| Sample 1-Sample 2 | Test Statistic | Std. Error | Std. Test Statistic | Sig. | Adj. Sig.^a^ |
| P6 T9-P6 T2 | 3.500 | 1.427 | 2.452 | .014 | .639 |
| P6 T9-P6 T1 | 4.889 | 1.427 | 3.425 | <.001 | .028 |
| P6 T8-P6 T6 | .222 | 1.427 | .156 | .876 | 1.000 |
| P6 T8-P6 T5 | .778 | 1.427 | .545 | .586 | 1.000 |
| P6 T8-P6 T4 | 1.611 | 1.427 | 1.129 | .259 | 1.000 |
| P6 T8-P6 T3 | 2.222 | 1.427 | 1.557 | .119 | 1.000 |
| P6 T8-P6 T2 | 2.278 | 1.427 | 1.596 | .111 | 1.000 |
| P6 T8-P6 T1 | 3.667 | 1.427 | 2.569 | .010 | .459 |
| P6 T6-P6 T5 | .556 | 1.427 | .389 | .697 | 1.000 |
| P6 T6-P6 T4 | 1.389 | 1.427 | .973 | .330 | 1.000 |
| P6 T6-P6 T3 | 2.000 | 1.427 | 1.401 | .161 | 1.000 |
| P6 T6-P6 T2 | 2.056 | 1.427 | 1.440 | .150 | 1.000 |
| P6 T6-P6 T1 | 3.444 | 1.427 | 2.413 | .016 | .711 |
| P6 T5-P6 T4 | .833 | 1.427 | .584 | .559 | 1.000 |
| P6 T5-P6 T3 | 1.444 | 1.427 | 1.012 | .312 | 1.000 |
| P6 T5-P6 T2 | 1.500 | 1.427 | 1.051 | .293 | 1.000 |
| P6 T5-P6 T1 | 2.889 | 1.427 | 2.024 | .043 | 1.000 |
| P6 T4-P6 T3 | .611 | 1.427 | .428 | .669 | 1.000 |
| P6 T4-P6 T2 | .667 | 1.427 | .467 | .640 | 1.000 |
| P6 T4-P6 T1 | 2.056 | 1.427 | 1.440 | .150 | 1.000 |
| P6 T3-P6 T2 | .056 | 1.427 | .039 | .969 | 1.000 |
| P6 T3-P6 T1 | 1.444 | 1.427 | 1.012 | .312 | 1.000 |
| P6 T2-P6 T1 | 1.389 | 1.427 | .973 | .330 | 1.000 |
| Each row tests the null hypothesis that the Sample 1 and Sample 2 distributions are the same.  Asymptotic significances (2-sided tests) are displayed. The significance level is .050. | | | | | |
| a. Significance values have been adjusted by the Bonferroni correction for multiple tests. | | | | | |

**Table 127:** Comparison of P7T1 to P7T10 in the Control group

**Table 128:** Multiple comparisons of P7T1 to P7T10 in the Control group

| Sample 1-Sample 2 | Test Statistic | Std. Error | Std. Test Statistic | Sig. | Adj. Sig.^a^ |
| --- | --- | --- | --- | --- | --- |
| P7 T10-P7 T9 | 1.688 | 1.514 | 1.115 | .265 | 1.000 |
| P7 T10-P7 T7 | 1.938 | 1.514 | 1.280 | .201 | 1.000 |
| P7 T10-P7 T8 | 2.688 | 1.514 | 1.775 | .076 | 1.000 |
| P7 T10-P7 T6 | 3.313 | 1.514 | 2.188 | .029 | 1.000 |
| P7 T10-P7 T5 | 3.688 | 1.514 | 2.436 | .015 | .668 |
| P7 T10-P7 T4 | 3.875 | 1.514 | 2.560 | .010 | .471 |
| P7 T10-P7 T3 | 4.063 | 1.514 | 2.684 | .007 | .328 |
| P7 T10-P7 T2 | 5.688 | 1.514 | 3.757 | <.001 | .008 |
| P7 T10-P7 T1 | 6.188 | 1.514 | 4.087 | <.001 | .002 |
| P7 T9-P7 T7 | .250 | 1.514 | .165 | .869 | 1.000 |
| P7 T9-P7 T8 | 1.000 | 1.514 | .661 | .509 | 1.000 |
| P7 T9-P7 T6 | 1.625 | 1.514 | 1.073 | .283 | 1.000 |
| P7 T9-P7 T5 | 2.000 | 1.514 | 1.321 | .186 | 1.000 |
| P7 T9-P7 T4 | 2.188 | 1.514 | 1.445 | .148 | 1.000 |
| P7 T9-P7 T3 | 2.375 | 1.514 | 1.569 | .117 | 1.000 |
| P7 T9-P7 T2 | 4.000 | 1.514 | 2.642 | .008 | .371 |
| P7 T9-P7 T1 | 4.500 | 1.514 | 2.973 | .003 | .133 |
| P7 T7-P7 T8 | -.750 | 1.514 | -.495 | .620 | 1.000 |
| P7 T7-P7 T6 | 1.375 | 1.514 | .908 | .364 | 1.000 |
| P7 T7-P7 T5 | 1.750 | 1.514 | 1.156 | .248 | 1.000 |
| P7 T7-P7 T4 | 1.938 | 1.514 | 1.280 | .201 | 1.000 |
| P7 T7-P7 T3 | 2.125 | 1.514 | 1.404 | .160 | 1.000 |
| Sample 1-Sample 2 | Test Statistic | Std. Error | Std. Test Statistic | Sig. | Adj. Sig.^a^ |
| P7 T7-P7 T2 | 3.750 | 1.514 | 2.477 | .013 | .596 |
| P7 T7-P7 T1 | 4.250 | 1.514 | 2.807 | .005 | .225 |
| P7 T8-P7 T6 | .625 | 1.514 | .413 | .680 | 1.000 |
| P7 T8-P7 T5 | 1.000 | 1.514 | .661 | .509 | 1.000 |
| P7 T8-P7 T4 | 1.188 | 1.514 | .784 | .433 | 1.000 |
| P7 T8-P7 T3 | 1.375 | 1.514 | .908 | .364 | 1.000 |
| P7 T8-P7 T2 | 3.000 | 1.514 | 1.982 | .048 | 1.000 |
| P7 T8-P7 T1 | 3.500 | 1.514 | 2.312 | .021 | .935 |
| P7 T6-P7 T5 | .375 | 1.514 | .248 | .804 | 1.000 |
| P7 T6-P7 T4 | .563 | 1.514 | .372 | .710 | 1.000 |
| P7 T6-P7 T3 | .750 | 1.514 | .495 | .620 | 1.000 |
| P7 T6-P7 T2 | 2.375 | 1.514 | 1.569 | .117 | 1.000 |
| P7 T6-P7 T1 | 2.875 | 1.514 | 1.899 | .058 | 1.000 |
| P7 T5-P7 T4 | .188 | 1.514 | .124 | .901 | 1.000 |
| P7 T5-P7 T3 | .375 | 1.514 | .248 | .804 | 1.000 |
| P7 T5-P7 T2 | 2.000 | 1.514 | 1.321 | .186 | 1.000 |
| P7 T5-P7 T1 | 2.500 | 1.514 | 1.651 | .099 | 1.000 |
| P7 T4-P7 T3 | .188 | 1.514 | .124 | .901 | 1.000 |
| P7 T4-P7 T2 | 1.813 | 1.514 | 1.197 | .231 | 1.000 |
| P7 T4-P7 T1 | 2.313 | 1.514 | 1.528 | .127 | 1.000 |
| P7 T3-P7 T2 | 1.625 | 1.514 | 1.073 | .283 | 1.000 |
| P7 T3-P7 T1 | 2.125 | 1.514 | 1.404 | .160 | 1.000 |
| P7 T2-P7 T1 | .500 | 1.514 | .330 | .741 | 1.000 |
| Each row tests the null hypothesis that the Sample 1 and Sample 2 distributions are the same.  Asymptotic significances (2-sided tests) are displayed. The significance level is .050. | | | | | |
| a. Significance values have been adjusted by the Bonferroni correction for multiple tests. | | | | | |

**Table 129:** Comparison of P8T1 to P8T10 in the Control group

**Table 130:** Multiple comparisons of P8T1 to P8T10 in the Control group

| Sample 1-Sample 2 | Test Statistic | Std. Error | Std. Test Statistic | Sig. | Adj. Sig.^a^ |
| --- | --- | --- | --- | --- | --- |
| P8 T9-P8 T10 | -.333 | 1.748 | -.191 | .849 | 1.000 |
| P8 T9-P8 T8 | .667 | 1.748 | .381 | .703 | 1.000 |
| P8 T9-P8 T7 | 1.250 | 1.748 | .715 | .475 | 1.000 |
| P8 T9-P8 T5 | 2.250 | 1.748 | 1.287 | .198 | 1.000 |
| P8 T9-P8 T3 | 2.333 | 1.748 | 1.335 | .182 | 1.000 |
| P8 T9-P8 T6 | 2.667 | 1.748 | 1.526 | .127 | 1.000 |
| P8 T9-P8 T4 | 2.750 | 1.748 | 1.573 | .116 | 1.000 |
| P8 T9-P8 T2 | 3.750 | 1.748 | 2.145 | .032 | 1.000 |
| P8 T9-P8 T1 | 5.667 | 1.748 | 3.242 | .001 | .053 |
| P8 T10-P8 T8 | .333 | 1.748 | .191 | .849 | 1.000 |
| P8 T10-P8 T7 | .917 | 1.748 | .524 | .600 | 1.000 |
| P8 T10-P8 T5 | 1.917 | 1.748 | 1.096 | .273 | 1.000 |
| P8 T10-P8 T3 | 2.000 | 1.748 | 1.144 | .253 | 1.000 |
| P8 T10-P8 T6 | 2.333 | 1.748 | 1.335 | .182 | 1.000 |
| P8 T10-P8 T4 | 2.417 | 1.748 | 1.383 | .167 | 1.000 |
| P8 T10-P8 T2 | 3.417 | 1.748 | 1.955 | .051 | 1.000 |
| P8 T10-P8 T1 | 5.333 | 1.748 | 3.051 | .002 | .103 |
| P8 T8-P8 T7 | .583 | 1.748 | .334 | .739 | 1.000 |
| P8 T8-P8 T5 | 1.583 | 1.748 | .906 | .365 | 1.000 |
| P8 T8-P8 T3 | 1.667 | 1.748 | .953 | .340 | 1.000 |
| P8 T8-P8 T6 | 2.000 | 1.748 | 1.144 | .253 | 1.000 |
| P8 T8-P8 T4 | 2.083 | 1.748 | 1.192 | .233 | 1.000 |
| Sample 1-Sample 2 | Test Statistic | Std. Error | Std. Test Statistic | Sig. | Adj. Sig.^a^ |
| P8 T8-P8 T2 | 3.083 | 1.748 | 1.764 | .078 | 1.000 |
| P8 T8-P8 T1 | 5.000 | 1.748 | 2.860 | .004 | .190 |
| P8 T7-P8 T5 | 1.000 | 1.748 | .572 | .567 | 1.000 |
| P8 T7-P8 T3 | 1.083 | 1.748 | .620 | .535 | 1.000 |
| P8 T7-P8 T6 | 1.417 | 1.748 | .810 | .418 | 1.000 |
| P8 T7-P8 T4 | 1.500 | 1.748 | .858 | .391 | 1.000 |
| P8 T7-P8 T2 | 2.500 | 1.748 | 1.430 | .153 | 1.000 |
| P8 T7-P8 T1 | 4.417 | 1.748 | 2.527 | .012 | .518 |
| P8 T5-P8 T3 | .083 | 1.748 | .048 | .962 | 1.000 |
| P8 T5-P8 T6 | -.417 | 1.748 | -.238 | .812 | 1.000 |
| P8 T5-P8 T4 | .500 | 1.748 | .286 | .775 | 1.000 |
| P8 T5-P8 T2 | 1.500 | 1.748 | .858 | .391 | 1.000 |
| P8 T5-P8 T1 | 3.417 | 1.748 | 1.955 | .051 | 1.000 |
| P8 T3-P8 T6 | -.333 | 1.748 | -.191 | .849 | 1.000 |
| P8 T3-P8 T4 | -.417 | 1.748 | -.238 | .812 | 1.000 |
| P8 T3-P8 T2 | 1.417 | 1.748 | .810 | .418 | 1.000 |
| P8 T3-P8 T1 | 3.333 | 1.748 | 1.907 | .057 | 1.000 |
| P8 T6-P8 T4 | .083 | 1.748 | .048 | .962 | 1.000 |
| P8 T6-P8 T2 | 1.083 | 1.748 | .620 | .535 | 1.000 |
| P8 T6-P8 T1 | 3.000 | 1.748 | 1.716 | .086 | 1.000 |
| P8 T4-P8 T2 | 1.000 | 1.748 | .572 | .567 | 1.000 |
| P8 T4-P8 T1 | 2.917 | 1.748 | 1.669 | .095 | 1.000 |
| P8 T2-P8 T1 | 1.917 | 1.748 | 1.096 | .273 | 1.000 |
| Each row tests the null hypothesis that the Sample 1 and Sample 2 distributions are the same.  Asymptotic significances (2-sided tests) are displayed. The significance level is .050. | | | | | |
| a. Significance values have been adjusted by the Bonferroni correction for multiple tests. | | | | | |

**Table 131:** Comparison of P9T1 to P9T10 in the Control group

**Table 132:** Comparison of P10T1 to P10T10 in the Control group

## PQ: Friedman’s test for median-based variables

**Table 133:** Friedman’s test results for the median-based variables T1 to T6, in the Intervention group

**Table 134:** Multiple comparison results for the time-related median-based variables T1 to T8, in the Intervention group

**Table 135:** Friedman’s test results for the time-related median-based variables T1 to T10, in the Intervention group

**Table 136:** Friedman’s test results for the time-related median-based variables T1 to T6, in the Control group

**Table 137:** Multiple comparisons for the time-related median-based variables T1 to T6, in the Control group

**Table 138:** Friedman’s test results for the time-related median-based variables T1 to T8, in the Control group

**Table 139:** Multiple comparisons for the time-related median-based variables T1 to T8, in the Control group

**Table 140:** Friedman’s test results for the time-related median-based variables T1 to T10, in the Control group

**Table 141:** Multiple comparisons for Friedman’s test results for the time-related median-based variables T1 to T10, in the Control group

# Normality Tests

## Demographics

**Table 142:** Age

**Table 143:** Date of diagnosis

**Table 144:** Months between tests

## EQ-5D-5L

**Table 145**

## CORE-OM

**Table 146**

## ADHDRS

**Table 147**

## AAQoL

**Table 148**

## SRI

**Table 149**

## IAF

**Table 150**

## PQ

**Table 151:** MedT1

**Table 152:** Normality tests for PQ’s medians T1 to T10, in the Intervention group

**Table 153:** Normality tests for PQ’s medians T1 to T10, in the Control group

# Independence at pretest

**Table 154:** Hypotheses and decisions for Total scores and Median-based PQ

**Table 155:** Mann-Whitney’s U for EQ5L5D Total scores at pretest

**Table 156:** Mann-Whitney’s U for AQQoL scores at pretest

**Table 157:** Mann-Whitney’s U for CORE-OM scores at pretest

**Table 158**: Mann-Whitney’s U for IAF scores at pretest

**Table 159:** Mann-Whitney’s U for SRI scores at pretest

**Table 160:** Mann-Whitney’s U for ADHDSR scores at pretest

**Table 161:** Mann-Whitney’s U for PQ Median T1 scores at pretest

**Table 162:** Hypothesis testing fo Mann-Whitney’s U, for PQ median scores for every moment, T1 to T10

**Table 163:** Test statistics for median T10 scores
