# Supplementary material for: A Neuroaffirmative, Self-Determination Theory–Based Psychosocial Intervention for Adults With Attention-Deficit/Hyperactivity Disorder: Randomized Feasibility Study
Source: JMIR Form Res. 2025 Oct 29;9:e69943. doi: 10.2196/69943 (PMC12612647; doi:10.2196/69943)
Supplement: Multimedia Appendix 5 [file formative_v9i1e69943_app5.docx]

# Supplemental Material 3: Measures

## EQ-5D-5L

Under each heading, please tick the ONE box that best describes your health TODAY.

MOBILITY

I have no problems in walking about

I have slight problems in walking about

I have moderate problems in walking about

I have severe problems in walking about

I am unable to walk about

SELF-CARE

I have no problems washing or dressing myself

I have slight problems washing or dressing myself

I have moderate problems washing or dressing myself

I have severe problems washing or dressing myself

I am unable to wash or dress myself

USUAL ACTIVITIES (e.g. work, study, housework, family or leisure activities)

I have no problems doing my usual activities

I have slight problems doing my usual activities

I have moderate problems doing my usual activities

I have severe problems doing my usual activities

I am unable to do my usual activities

PAIN / DISCOMFORT

I have no pain or discomfort

I have slight pain or discomfort

I have moderate pain or discomfort

I have severe pain or discomfort

I have extreme pain or discomfort

ANXIETY / DEPRESSION

I am not anxious or depressed

I am slightly anxious or depressed

I am moderately anxious or depressed

I am severely anxious or depressed

I am extremely anxious or depressed

## Clinical Outcomes in Routine Evaluation – Outcome Measure (CORE-OM)


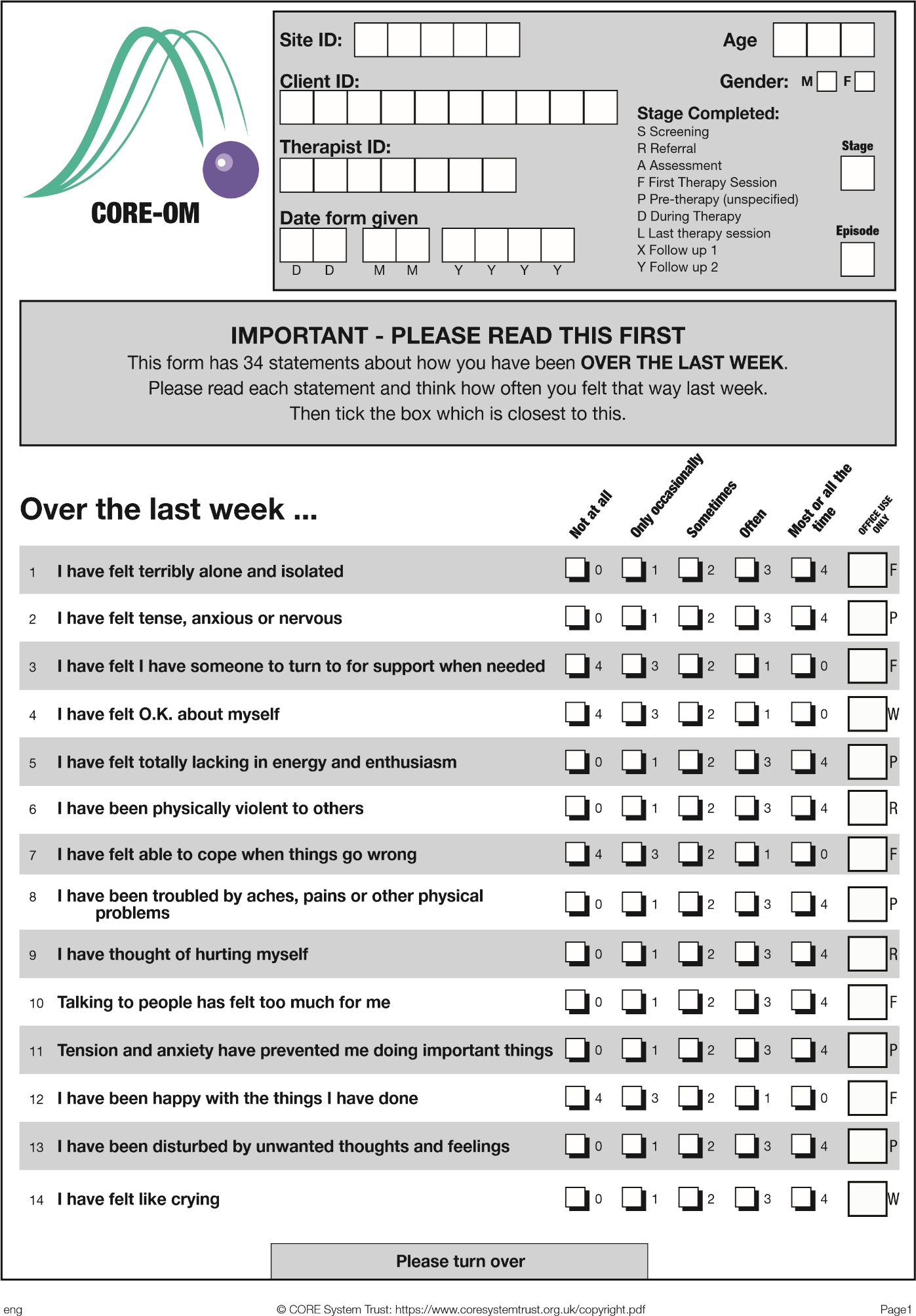


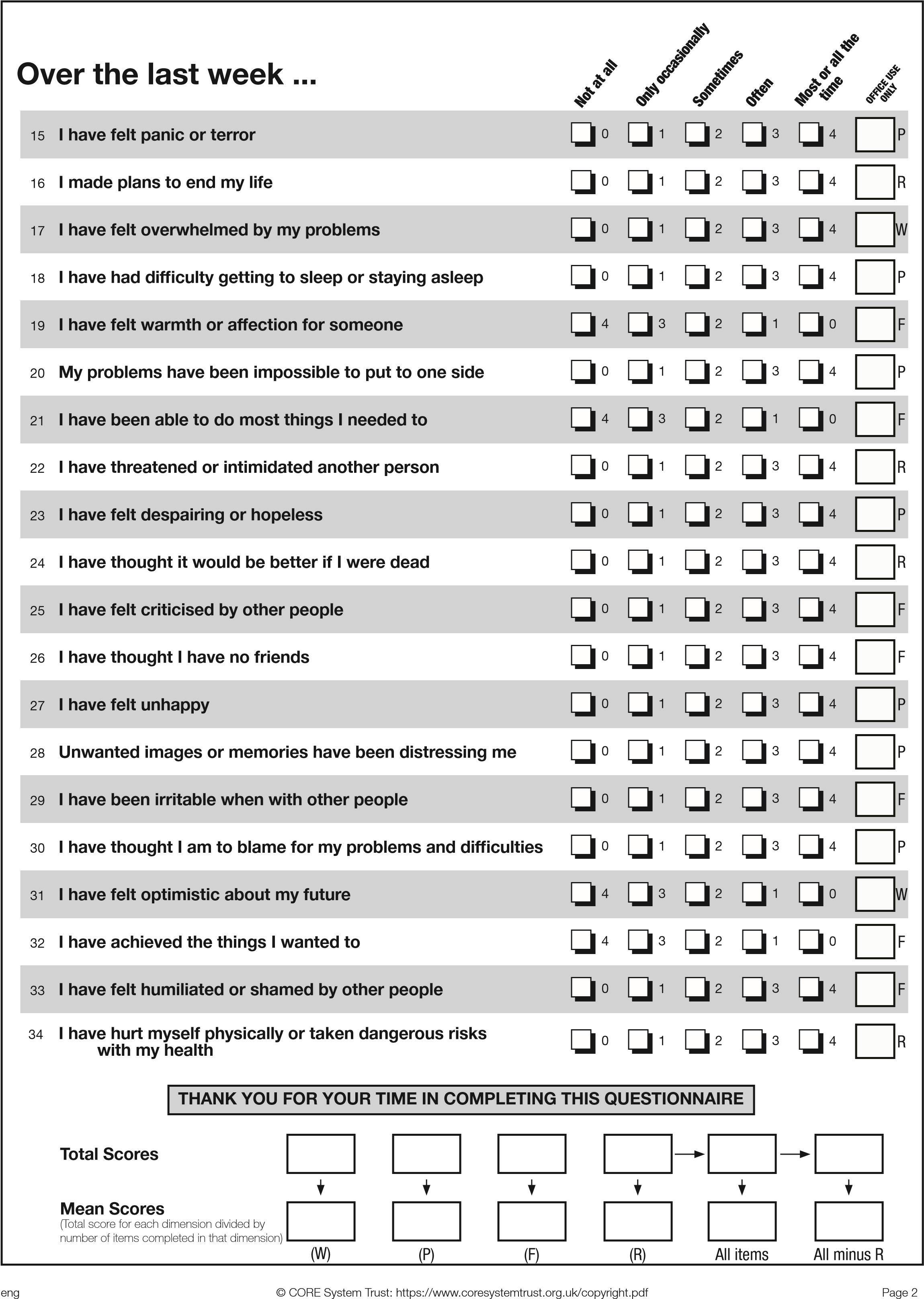


## Attention Deficit Hyperactivity Disorder Rating Scale, Investigator-Administered (ADHDRS-IV-Inv)

| **Items** | Not at all, never | Just a little, once in a while | Pretty much, often | Very much, frequently |
| --- | --- | --- | --- | --- |
| 1. Loses things necessary for tasks and activities (1) | 0 1 2 3 | | | |
| 2. Talks too much (2) | 0 1 2 3 | | | |
| 3. Gets rowdy or boisterous doing leisure activities (4) | 0 1 2 3 | | | |
| 4. Leaves seat when not supposed to (6) | 0 1 2 3 | | | |
| 5. Has trouble waiting in line or taking turns with others (8) | 0 1 2 3 | | | |
| 6. Has trouble keeping attention focused when working or at leisure (9) | 0 1 2 3 | | | |
| 7. Is forgetful in daily activities (13) | 0 1 2 3 | | | |
| 8. Has trouble listening to what other people are saying (14) | 0 1 2 3 | | | |
| 9. Is always ‘on the go’ (16) | 0 1 2 3 | | | |
| 10. Fidgets with hands or feet or squirms in seat (18) | 0 1 2 3 | | | |
| 11. makes careless mistakes or has trouble paying close attention to detail (19) | 0 1 2 3 | | | |
| 12. Does not like academic studies/work projects where effort at thinking a lot is required (21) | 0 1 2 3 | | | |
| 13. Is restless or overactive (22) | 0 1 2 3 | | | |
| 14. Gives answers to questions before the questions have been completed (25) | 0 1 2 3 | | | |
| 15. Has trouble finishing job tasks or school work (26) | 0 1 2 3 | | | |
| 16. Interrupts others when they are working or busy (27) | 0 1 2 3 | | | |
| 17. Appears distracted when things are going on around him/her (29) | 0 1 2 3 | | | |
| 18. Has problems organising tasks and activities (30) | 0 1 2 3 | | | |

## Attention Deficit Hyperactivity Disorder Quality of Life Scale (AAQoL)

|  | Never | Almost never | Sometimes | Fairly often | Very often |
| --- | --- | --- | --- | --- | --- |
| Life Productivity |  |  |  |  |  |
| Get things done on time | 1 | 2 | 3 | 4 | 5 |
| Complete projects or tasks | 1 | 2 | 3 | 4 | 5 |
| Balance multiple projects | 1 | 2 | 3 | 4 | 5 |
| Remember important things | 1 | 2 | 3 | 4 | 5 |
| Get started with tasks you don't find interesting | 1 | 2 | 3 | 4 | 5 |
| Keep track of important items | 1 | 2 | 3 | 4 | 5 |
| Keep house clean | 1 | 2 | 3 | 4 | 5 |
| Manage finances | 1 | 2 | 3 | 4 | 5 |
| Get your shopping done | 1 | 2 | 3 | 4 | 5 |
| Pay attention | 1 | 2 | 3 | 4 | 5 |
| Getting things done requires too much effort | 1 | 2 | 3 | 4 | 5 |
| Psychological Health |  |  |  |  |  |
| Anxious | 1 | 2 | 3 | 4 | 5 |
| Overwhelmed | 1 | 2 | 3 | 4 | 5 |
| Fluctuations in emotions | 1 | 2 | 3 | 4 | 5 |
| Depressed | 1 | 2 | 3 | 4 | 5 |
| You have overreacted in difficult situations | 1 | 2 | 3 | 4 | 5 |
| Feeling fatigued | 1 | 2 | 3 | 4 | 5 |
| Life outlook |  |  |  |  |  |
| Your energy is well spent | 1 | 2 | 3 | 4 | 5 |
| Feel good about yourself | 1 | 2 | 3 | 4 | 5 |
| People enjoy spending time with you | 1 | 2 | 3 | 4 | 5 |
| You can successfully manage your life | 1 | 2 | 3 | 4 | 5 |
| Able to enjoy time spent with others | 1 | 2 | 3 | 4 | 5 |
| As productive as you would like to be | 1 | 2 | 3 | 4 | 5 |
| Your intimate relationship is going well | 1 | 2 | 3 | 4 | 5 |
| Relationships |  |  |  |  |  |
| You annoyed people | 1 | 2 | 3 | 4 | 5 |
| People are frustrated with you | 1 | 2 | 3 | 4 | 5 |
| Tension in relationships | 1 | 2 | 3 | 4 | 5 |
| Not having quality time with others | 1 | 2 | 3 | 4 | 5 |
| You have not been able to meet others expectations | 1 | 2 | 3 | 4 | 5 |

## Self-Reflection and Insight Scale (SR&I)

|  | Strongly disagree | Moderately disagree | Slightly disagree | Slightly Agree | Moderately Agree | Strongly Agree |
| --- | --- | --- | --- | --- | --- | --- |
| Engagement in Self-reflection |  |  |  |  |  |  |
| I don’t often think about my thoughts | 1 | 2 | 3 | 4 | 5 | 6 |
| I rarely spend time in self-reflection | 1 | 2 | 3 | 4 | 5 | 6 |
| I frequently examine my feelings | 1 | 2 | 3 | 4 | 5 | 6 |
| I don’t really think about why I behave the way I do | 1 | 2 | 3 | 4 | 5 | 6 |
| I frequently take time to reflect on my thoughts | 1 | 2 | 3 | 4 | 5 | 6 |
| I often think about the way I feel about things | 1 | 2 | 3 | 4 | 5 | 6 |
| Need for self-reflection |  |  |  |  |  |  |
| I am not really interested in analysing my behaviour | 1 | 2 | 3 | 4 | 5 | 6 |
| It is important for me to evaluate the things that I do | 1 | 2 | 3 | 4 | 5 | 6 |
| I am very interested in examining what I think about | 1 | 2 | 3 | 4 | 5 | 6 |
| It is important for me to try to understand what my feelings mean | 1 | 2 | 3 | 4 | 5 | 6 |
| I have a definite need to understand the way that my mind works | 1 | 2 | 3 | 4 | 5 | 6 |
| It is important to me to be able to understand how my thoughts arise | 1 | 2 | 3 | 4 | 5 | 6 |
| Insight |  |  |  |  |  |  |
| I am usually aware of my thoughts | 1 | 2 | 3 | 4 | 5 | 6 |
| I’m often confused about the way that I really feel about things | 1 | 2 | 3 | 4 | 5 | 6 |
| I usually have a very clear idea about why I’ve behaved in a certain way | 1 | 2 | 3 | 4 | 5 | 6 |
| I’m often aware that I’m having a feeling, but I often don’t quite know what it is | 1 | 2 | 3 | 4 | 5 | 6 |
| My behaviour often puzzles me | 1 | 2 | 3 | 4 | 5 | 6 |
| Thinking about my thought makes me more confused | 1 | 2 | 3 | 4 | 5 | 6 |
| I often find it difficult to make sense of the way I feel about things | 1 | 2 | 3 | 4 | 5 | 6 |
| I usually know why I feel the way I do | 1 | 2 | 3 | 4 | 5 | 6 |

## Index of Autonomous Functioning (IAF)

|  | Not at all true | A bit true | Somewhat true | Mostly true | Completely true |
| --- | --- | --- | --- | --- | --- |
| My decisions represent my most important values and feelings | 1 | 2 | 3 | 4 | 5 |
| I do things in order to avoid feeling badly about myself | 1 | 2 | 3 | 4 | 5 |
| I often reflect on why I react the way I do | 1 | 2 | 3 | 4 | 5 |
| I strongly identify with the things that I do | 1 | 2 | 3 | 4 | 5 |
| I am deeply curious when I react with fear or anxiety to events in my life | 1 | 2 | 3 | 4 | 5 |
| I do a lot of things to avoid feeling ashamed | 1 | 2 | 3 | 4 | 5 |
| I try to manipulate myself into doing certain things | 1 | 2 | 3 | 4 | 5 |
| My actions are congruent with who I really am | 1 | 2 | 3 | 4 | 5 |
| I am interested in understanding the reasons for my actions | 1 | 2 | 3 | 4 | 5 |
| My whole self stands behind the important decisions I make | 1 | 2 | 3 | 4 | 5 |
| I believe certain things so that others will like me | 1 | 2 | 3 | 4 | 5 |
| I am interested in why I act the way I do | 1 | 2 | 3 | 4 | 5 |
| I like to investigate my feelings | 1 | 2 | 3 | 4 | 5 |
| I often pressure myself | 1 | 2 | 3 | 4 | 5 |
| My decisions are steadily informed by things I want or care about | 1 | 2 | 3 | 4 | 5 |

## Personal Questionnaire (PQ)

Instructions: Please complete before each session. Rate each of the following problems according to how much it has bothered you during the past seven days, including today.

|  | Not At All | Very Little | Little | Moderately | Considerably | Very Considerably | Maximum Possible |
| --- | --- | --- | --- | --- | --- | --- | --- |
| 1. | 1 | 2 | 3 | 4 | 5 | 6 | 7 |
| 2. | 1 | 2 | 3 | 4 | 5 | 6 | 7 |
| 3. | 1 | 2 | 3 | 4 | 5 | 6 | 7 |
| 4. | 1 | 2 | 3 | 4 | 5 | 6 | 7 |
| 5. | 1 | 2 | 3 | 4 | 5 | 6 | 7 |
| 6. | 1 | 2 | 3 | 4 | 5 | 6 | 7 |
| 7. | 1 | 2 | 3 | 4 | 5 | 6 | 7 |
| 8. | 1 | 2 | 3 | 4 | 5 | 6 | 7 |
| 9 | 1 | 2 | 3 | 4 | 5 | 6 | 7 |
| 10. | 1 | 2 | 3 | 4 | 5 | 6 | 7 |
