# Supplementary material for: A Neuroaffirmative, Self-Determination Theory–Based Psychosocial Intervention for Adults With Attention-Deficit/Hyperactivity Disorder: Randomized Feasibility Study
Source: JMIR Form Res. 2025 Oct 29;9:e69943. doi: 10.2196/69943 (PMC12612647; doi:10.2196/69943)
Supplement: Multimedia Appendix 6 [file formative_v9i1e69943_app6.docx]

# Supplementary Material 5: Thematic analysis

| Participant | Group | Themes | Codes | Quote |
| --- | --- | --- | --- | --- |
| 1 | Group 1 |  | Conscious choice  Following self-direction  Sense of satisfaction  Developing good habits  Awareness of choice  Sense of urgency vs anxiety  Less overwhelm and still active  Different perspective  Awareness and curiosity around progress  Recognition of choices and actions  Maintenance vs progress, but recognition it is still action  Recognising emotions as influences on everyday mood  Issues with autonomy link development experiences  Accountability  Easier to externalise exploring and reflecting  Tasks feel more autonomous with meaning | Useful - therapy style reflection to explore where I am, easier to know what I'm thinking when I have to explain to someone  It's been massively more successful because despite really like I said, throwing myself into the what really shouldn't be my priority. But I've decided for the last week I am doing that. Despite doing that. I have been every day putting down post it notes and actually getting all the other stuff done as well which? I guess. Because it's stuff that I really should be doing and have to do. I just kind of I don't notice I've done it. You know what I mean, but I certainly notice when I haven't done. But this week I've just been doing all the things that normally I just wouldn't do. So I guess it's been quite successful  So of all the things I really should be doing to present to you, I haven't really done them like for six months though. But but on the other hand, I am developing really good day by day habits and I and I know it's more like everything I'm doing is a choice. The guilt is the choice, but in a way that no in the way the guilt is good. Because if I don't have the guilt, once this piece is over, I'm just going to do it again. And I need that guilt to stop me from doing it. Yes, because I'm. I'm already feeling the urgency of all the things I need to do to be moving out like by January next year and vast progress that I'm. Doing urgency about something that's months away. That's definite progress. Which is what I say to my students when they're going in for exams, which is have urgency for this thing that's months away, not panic. Yeah, but urgency. Yeah. Are not anxiety. Yeah. Urgency. Yeah, Which is which is new, which is new for me because usually it's just anxiety and then I just shut it down. So yeah. Hmm. Urgency. It's like I can do this. It's like it's the same as anxiety, but with a no. I I got this like this is possible. I'm not overwhelmed. I've never really thought about that. But. But it is. It's the same bodily feeling, but with with a different belief system on top of it.  Well, I I think what it is is that I'm trying to be much better. At sort of. All the time. Am I moving towards my six month goal? Am I moving? In that direction. And I'm not. I'm not at all moving in that direction and then immediately I'm like, ohh well, my week's been a disaster. But in many ways, I think that's a healthy thing that I'm sort of going, oh, my God, what's happened? I've not been moving in that direction. But then I'm I'm sort of have to. Yeah. But then I have to keep reminding myself. No, I I made the decision to put that. On hold for. A week but. But anyways, actually that's a useful. That's a useful state, although there's a fine line between that. And just me all the time where I never have seen the positive. But it's a fine line. But but I I'm hoping that when I say OK enough with this piece and this next performance that then every time I do something moving in that direction. I'm not just going to. Then at the end of that day going Oh my God, I haven't done anything. I will be like, right. I'm moving in that direction, but I'm just not at the moment, you know? And although to be fair, in the last week I've been staying on top more or less. Of all of the teaching stuff, OK, so, but that's more like maintenance rather than rather than increasing, but normally the I don't have the maintenance, but yeah.  “This is so useful I can feel the change in the way that I think, and recently I've started feeling that if I'm not careful, I can really slip back into the depression. And I've realized that one of the overwhelming characteristics of being in that Is when I start to dwell on memories that give me painful emotions. And they start to happen more and more. It's not exactly that I choose to dwell on them. It's just that they're there and I don't know what to do with them, except I kind of do know what to do with them, because when I did that exercise a couple of weeks ago, they were like, every time I have one of these memories, there's been painful emotions. If I sit down and write down What is the positives that I could take away From it actually?  Changes my entire mood and I've realized, OK, they they come over me and I and they're just there and I don't do anything with them. But it's hard. And I'm realizing that I I have to actually just make. The time to sit down and do something with them, I feel like I don't quite have that time, but I've realized that kind of it works. I know it works so so I need to definitely make time for that in the next week because that was so powerful and every time. Came up that made me feel sad or a little bit of grief or or like, you know, just mostly sadness. But but there are ways I can consciously sit down and go instead of just every time I think it's. I'm just overwhelmed with the negative emotion.  “  I think this is wow, this is one of the most useful sort of concepts where actually I've come across that and self determination theory which has been a massive game changer. The whole concept of autonomy I think. But I think the felt sense of autonomy and what it is in in the in the deep sense and the spectrum in. Two, it's just one of the most deeply profound things to reflect on, I think, because I think that There's a lot of overlap between adverse childhood experiences, attachment issues. and trauma and I think to what they all share in common are issues to do with autonomy. And I think this is huge, so that's useful.  I think mostly because it helps me, I think I think all this stuff we spoke about in the beginning was interesting. But I think for me personally, I don't think it's sort of really practical and For me, it was really the accountability and I think what I find useful is almost more like a therapist for me, just to explore where I'm at in my reflection, because it just sort of sometimes It's much easier for me to know what I'm thinking when I have to describe it to somebody.  Maybe for some people who don't already have that as a habit when they're writing down their their projects and their to do this to go, why am I doing this? And then it does start to bear over and towards acts and stuff like that. We just remind ourselves why we're aligns with the values and everything. Umm cause? Cause I think that's that's really a weak part of my my whole process that just everything becomes a whole a giant list of shoulds with pressure which is why I don't do them even though it's come from me. I mean like like a lot of the time. I say OK, I'm gonna do this, but I still don't feel the autonomy because it's kind of come. It doesn't feel it doesn't have that same sense of proper inner autonomy. |
| 2 | Group 1 |  | Help for self  Support for self expertise  Support for self design  Clarity in managing time by externalising with tools  Anxiety reduction  Acceptance of needs and difference | Very useful – Amazing  Well, the stuff I've done is helping others and this was helping me. So it was like payback time and.  I kind of like the way where you [do this], actually, it's like you're driving a car with someone next to you rather than someone telling you what to do. And I think therapies where you tell an ADHD what to do, I think it's. Just not gonna be work for others. I think the reason a lot of us are freelance is because we hate being told what to do, you know? don't really do well, if someone goes right, you must do this. This is how you need to run. Your life. I don't think that's why I think psychotherapy is. For ADHD people. You know, because it it you know, it gives us a say in how we want to do things. And I also from I must have read so many books and how to stop procrastinating and none of. Them ever helped me. And you know, I think because maybe the way that our ADHD. Brains work, but it's a spectrum condition, isn't it? Umm, so it differs from person to person? So you know what works for me might not work for someone else.  Ohh [this program] is perfect. You know, like I said, I'm a digital only person cause I lose paper. Yeah, so I've been able to use the apps that I found work for me and improve them. You know? So basically I did on my I do have my weekly planner in good notes which I designed. Yeah, but it's the same for me. It's not like someone who's got a bullet journal telling me that I've got. To do it like. This. Yeah. Which I would like. I really, you know, it's like I designed it how I wanted. But with your help, you helping me steer it, it was sort of fine tuning it, you know, in a way that it worked better.  Yeah, Google Calendar. Color-coded calendar has really helped me see how much time I've got.  Because one thing that I I have one thing that I did as a freelancer which always tips me up. Taking work on without having any idea how much free time. I've got to do it. You know, now I know exactly. Now, once I've inputted everything that I need to do in the way I'm, I'm very clear of how many hours I have left to. Do things. And that's been, you know, I think it's still, you know, obviously I'm still learning that I'm still learning how much time I actually have. You know like you know basically. I thought I was going to be finished with this clients project this last week and I haven't been because I was tired. But you know I didn't add any buffer time in and my attitude was like, well, I'm already bit too much to do because blah blah blah blah blah. But actually I even then I still need to add that buffer timing because I might need it. So you know, I think it's about, you know, learning to be realistic and having if something visual that I could look at. So that was like that was the thing that I did. You know, I didn't have a calendar. Yeah, I didn't use one. And I think the reason I didn't use them before was it wasn't visual enough. So having the color-coded thing so I know my I actually know what my color codes are and they're the same colours in to do is as they are on Google Calendar so you know it's like a blue like this for client work, lavender for meetings like this, yellow for days off, grey for church. Was green for taking my parents up, so I remember all those colours. Now those are those are the colours that go. They were signed and. And getting them across the system as well, you know. Yeah. So they're same  And also I think one of the biggest things from that that is actually really taken me from by surprise is how much my anxiety levels have dropped. How seldom I have anxiety dreams now. Whereas they were a daily thing. And that is, you know, I really really have to know when it's like really high stress thing, you know, like basically the day before I have to offer me my annual ADHD review or, you know, the day before we have to do the bloody trip to Scorpio, which kills everybody, you know, my anxiety levels have dropped.  And I think setting boundaries like, you know. Basically, deciding like the day before I have a big thing like that I've really gotta do nothing. Yeah, yeah, yeah, you know, and it's actually been a revelation that I've realized that, you know, you can't if you have to do it, because you will, they'll be paying back time. At some point, they'll burn out. You know, I'm not young anymore and you know I don't have the energy that someone who's 20 has, you know, like someone rambling on and LinkedIn about how weekends are for losers. And it's like, well, you know, you're 20. So you know, so I've got to, you know, it's been realistic. You know, I've had, I I've been to accept that I've got a disability. And, you know, just work with it rather than try and fight it all the time. You. Can't do that. |
| 3 | Group 2 |  | Easier externalising exploring and reflecting  Recognition and validation of positive traits | The key thing. That, I've realized, is just literally the importance of. Talking stuff out because I I think I said it before. I don't think there's been anything groundbreaking or anything that I any solution I hadn't already got to in my own head, but actually saying it out loud. For some reason has the effect of making you actually consider it. I might be able to get a solution in my head and think ohh that would help with this and I'll never think about it again, but obviously talking it through. Seems to have. Bring it to life a bit more. I guess so. Yeah. That's been that's been interesting. Like, I have learned some interesting things along the way as well. And obviously the the thing about it is because I know I'm going to have to talk about myself. But I'm thinking about what I'm going to talk about, which obviously prompts you to think about yourself. Which? Again, it has benefits as well because you've realized you realized you've realized something.  The other positive thing is I've always been a really positive person anywhere. Don't think I've ever had any form of depression or anything in my life because my brain just goes well. What? How do we fix that? And does so. I've never really sat on things for long negatively, but. I've mentioned it a few times over the course of this that before. I realized I had ADHD. I was always just looking at as a skill set. This is what I'm good at and this is what I'm bad at. And then when you find out. They're actually symptoms and it was a bit like, oh, so is all of what I consider to be my best traits now problematic because technically it's part of the disability and. I obviously immediately went now, but it is what it. Is it doesn't. It doesn't change anything, but it's it's good because you in when you're kind of in the community especially, I see quite a lot of people who are. Very hard on themselves and and they get very angry, which I feel bad for because I find. Half the stuff I do hilarious. When things cock up Yeah, I'm. I'm riding the benefit that I can from it as well. Yeah, I wouldn't be where I am if I didn't have ADHD, which is a weird one. But it is what it is. But it's it. It's been really positive. Even for me, who is a positive person to start off with anyway, just to have somebody go, Oh yeah. That is, uh, yeah, that's what you're good at. You. It's a benefit. Blah, blah, blah, blah, blah. Talking about it. And it definitely has an impact as well. |
| 4 | Group 2 |  | Turned life around  Feeling organised  Considered withdrawing but stuck with it  Sessions were helpful  Less overwhelmed  Using tools for memory  Recognition of memory issues  Feelings of capability | I feel like I've, you know, I've really really, you know, turned my life around like me. I don't ever thought, you know, like weeks and weeks ago. I mean, I don't know how many sessions we've done now. But and I never have thought that I could have got my life like they swear I'm, you know, organized and things like that. I did struggle sometimes where, you know. Where your ADHD? When you're like, I really don't wanna do the phone call and not not because of who's on the other side just because I don't want to do it. But I did it and I thought, well, my brother said no, no, you're only are not allowed to do a phone call. If there's something that's happening and there's an emergency, you've not got to do like you need to complete this because it's gonna. It's gonna be a positive thing for you. So I just kept, you know, saying to myself, no, no, stop thinking about that. We've got the phone call. We'll get it done. Talk about the things we need to do. And and it'll be a. It'll be a good thing. And I'm glad I did. I'm glad I said to myself that I wasn't gonna withdraw because they were. I might I just withdraw because I just feel like the hour. But that's just made today. You know, I might have someone else on my mind that I'm wanting to do. Uber I just said to myself, it's only like an hour after that hour, do whatever the hell you like, but that hour is that hour and I was to force myself and I said to my partner, I'm not gonna withdraw. I said no good. Don't withdraw because it's really, really helping. I think it's really helping you. So there were times where my my mind would spiral out and go, oh, I can't be bothered, but it would just cause I couldn't be. Bothered it. Wouldn't, but I'd talk to myself and say no, there's nothing that's getting in the way. I've done my things that I need to do. Get the phone call done. It'll do you good. It'll do. So. I had positive. Thoughts as well about at the same time. And that's what got me to say, right. Yeah. Let's go on. Zoom now. Let's go.  Yeah, you know, like where ADHD, you don't tend to complete tasks like, but like I we're just gonna give it up and go right. That's it. We're done now. You know. Bored now? Can't, can't be bothered sort of thing, but I won't really. Cause once I got on the phone, we are absolutely fine. And then conversation rolled and then I looked at time and I thought, wow, and I was gone by it's end of session. Now. Do you know bloody hell all that. All that complaining at the start. I've got this to do and I've got that to do well. I haven't really got anything to do, I've. Done it all. No, it's had a really and and you know what I said to my partner said I'm good. It's the last session today. Where bloody hell says. You. Oh, you did one on sometimes. About what? This phone call and I went. Yeah, but I won't have meant it that I would just bring an impulse. I was fine once I got. On the phone. You were like, yeah, you are. Actually. You were actually alright once you got on the phone, he said. It's done. You loads of good so.  Yeah, I'd recommend it to anybody. If anybody said to me, should I do the the you know, the ADHD research thing that I've been doing, I'd say I'd definitely go for it, cause it'd it'll really, really, really help. Yeah.  Just not, not as overwhelmed. Obviously in natural situations I'll get overwhelmed. Like yesterday. I would. I would cooking a Sunday dinner so. I were like ohh, getting a bit flustered, but then obviously my partner's friend said do you want me to mash the Mashed potato? I went. Ohh yes please, I will. Just about to get really overwhelmed. Right, alright. Yeah, that's fine. So Mashed potato. And once you've done that, I could then concentrate on dishing the children's meals up. So I felt a little bit more at ease and like, oh, someone just coming and rescued the situation from her.  Yeah, I was using the notebook writing stuff down every time I get something through on for their school, I'll write it down straight away. Right, because I'll just forget.  So it was true. I was saying to my sister weeks, probably months ago now, so I can't cause I've got a headache. I can't just do that. I can't do it and just went. You can just gotta change a couple of things, that's all you've gotta do. No, I can't, though, because my brain don't work like that. I'd be saying that. But now I I can say to heryou actually right? I I can. I can do those things. |
| 5 | Group 1 |  | Support for self design  Speaking to someone who understands  New understanding  Helps on everyday level  Recommend to everyone | Very useful - develop and use what I've got, speaking to someone who gets it  Very, very helpful. Help very helpful and and like useful like helping me. What's the word like develop and build on stuff that I've already got? But I don't know how to use properly if that makes sense. And just like the Being able to talk to someone that actually understands what you're going through is a massive help like. It's alright just talking to someone. Your doctors or whatever, and they're just like, are you trying to blah, blah? But we yourself it I can tell you what's going on in my brain. And to some degree, you'll understand and say right, well, This is why you're doing it and it and it's just helpful that there's. Someone that gets it. But yeah, overall I think it's been a massive help and. I think I would like to continue once I've got some money started again.  just like. I don't know. I don't know how to put. It, but I think everybody with a diagnosis should probably go through something similar just because of the amount that, like I've learned about myself and I say a short time, but it's not cause we've had mishaps and stuff like that, but. It it it's it's it's an eye opener and. And it helps a lot on like. Just an everyday sort of. Life type of thing. But yeah, I think. Long as show, everyone should have a go. At least once. |
| 6 | Group 2 |  | Positive perspective on ADHD  Polar experience  Sense of belonging  Not abnormal  Sense of peace and acceptance  Sense of capability  Negative view of current perspective and diagnostic criteria  Concern for others seeking diagnosis  Impact of negative feedback on identity  Concern for others  Belonging and accepting others express differently | You see, I and and and and. Yes, I mean, it's not always easy, but. I don't always see there's only a downside to having ADHD. I think there's an upside too. And and and. A lot of people don't recognize it.  They they. Definitely a benefit. Uh, I I I see many times there's a benefit of running at 100 miles an hour. I also do believe a pick up concept and grasp concepts twice as quickly as other people etc. And. I get things done. You know, so, so. Yes, as much as there is definitely sometimes downsides and it's almost tiring because of of what happens. There's definitely upsides as well.  If you ask me that question now. Do I have a lot different personal achieve more? Probably not. Am I happy because I understand I'm not the only clown who thinks this way? Yes. No, because. To me, the most refreshing thing this has brought is I'm not alone in the. World the way I think. Is is it? Is there an advantage? I've always operated the way I do and I don't think much has changed that way. Maybe I'll think a bit more about it than I did before, but I think what is positive to me. Is that? Is is that I'm not completely abnormal the way I think it and and I go about things and and and I think that's what's what's it's almost. I've always seen those. Advantages to it, but I think it's almost brought a bit of peace with the whole with the whole. Thing in that. You know, So what? There's. I'm not the only one out there. And yes, it does happen. And the way I think is pretty normal for someone with ADHD. And can I manage it? Yes and. If if you don't like the way I think I'm, I'm pretty much well unlucky for you. I I I think some of you you people out there without at all a little bit slur I agree yeah and and and and that's that's the way I think you know to be yeah. I I just wish some of you come on, get up to speed. Let's move.  To be frank, the 1st when I came to this country and the first person I spoke. To who interviewed me about ADHD for the medication and what have you said. I'm borderline I they don't think I've got ADHD because of this and because of that I said no, you must stop now because you must remember I was in that on that medication for years elsewhere, I've learned to manage this years ago. So what it does tell me. Is that treatment is successful and it is assisting me, so don't tell me I'm borderline and because I don't do this. And I'm not in trouble with the law. And I'm not about to jump off a building and because I can keep a job, doesn't mean I'm not. I'm. I'm. I'm not ADHD. And that unfortunately was someone who was interviewing me, who apparently knows a lot about ADHD. Which I found extremely disappointing. I told my wife that I said. To me, this is the fact that a professor. Of another country had had a look at it and written reports and what have you. No, you know the the information. She was saying what she thought she had determined over hour or hour and a half telephone conversation is that you know it's it's almost a misdiagnosis and that. I must admit and extremely naive, until the lady you what? I can't remember her name. Who's your supervisor on this project or your? I saw her and and straight away she knew. I mean, she knew and and and what have you and I just. I was very she. And in fact that first lady I spoke to had the decision whether she would allow me to go further and speak to someone further or not. Right. And and to me, you can almost leave people out there without support. By by having that approach. And nothing personal against her. She, to me, had a. Basis for Christians and because I didn't score over a certain amount and what I kept trying to say to. To a large degree, it's already under control because I've been on this medication for more than 10 years, I've learned. It but take me off the medication and say I don't have it and let's see if I go. Forward till I regret. Yeah, yeah. Positive somethings. So I'm glad you managed to get it sorted because that doesn't happen for everybody and it it can be really, really fraught and very difficult and then particularly. For people who Are seeking A diagnosis if they if they receive that negative feedback they then start to question themselves. Well, if it's not this then you know what? What's wrong with me. You see a night. Which is. Which which, rightly or wrongly, I'm if you are used to. On on ADHD medication and because someone misdiagnoses you just. Like that. I don't believe it's in anyone's best interest to just take people off because of of of a few minutes or hour on the telephone, you decided this was this or that. So much so, I phoned that woman back and I said to her, you know it, you might have whatever scores, but if you don't agree that I go further, I will ask for a second opinion. Anyway, I also you know it's this whole thing of of of being hereditary or not hereditary, but I was also trying to explain to her. I do believe that there is in the family. Maybe it wasn't diagnosed. And I also don't believe it was always diagnosed in the past because people didn't know so much about it and and. Also, do you believe that whether it's hereditary or not, it can start and end in a certain place? Doesn't mean that it's had to have been in there for the last 20 generations. Can start now and be hereditary going forward. Who knows. Yeah, I just, it's just I suppose that naivety of that people. You know, I mean, you can find a book that that ADHD for dummies. Or something that says. This is what will happen. That'll happen. That'll happen. It's it's it's very general. A lot.  Rebecca, it just worries me if if there's a person who's already down and they should be honest and they. Might not be excuse. The terminology is full of crap as me that I insist on things. It's they, don't they? You know, just they will get trampled all over and they will accept it and move on.  I think what I've got out of it the most is this. I'm not completely insane and I'm quite frankly, to be honest, even if I wasn't, so I didn't worry me. I was quite happy. So, so you know, it's just. Refreshing to know that there's others who think that. Right. And you know, you soon realize that. Many other people live with it, but also that other people show different symptoms. I know people who have got it, and I can tell you they're more. They're very much more subdued. |
| 7 | Group 2 |  | Experimental attitude  Manage overwhelm  Able to see reality of simple practice  Vulnerability  Honest self expression  Comfort with self honesty  Positive perspective more realistic  Useful framework and structure  Low effort key  More useful than quant indicators | m-hmm. I mean, and and we, we haven't yet, but it it it's like, but the idea is is, yeah, is to have the idea have to have the attitude of like well let's let's try it. Let's let's let's let's uh let's let's give ourselves let's do a hypothesis and let's test the hypothesis and then if that doesn't work we'll have another one. Yeah. That is, it's. It's a challenging frame of mind to to keep up with in my personal life. But you know. m-hmm. I mean, and and we, we haven't yet, but it it it's like, but the idea is is, yeah, is to have the idea have to have the attitude of like well let's let's try it. Let's let's let's let's uh let's let's give ourselves let's do a hypothesis and let's test the hypothesis and then if that doesn't work we'll have another one. Yeah. That is, it's. It's a challenging frame of mind to to keep up with in my personal life. But you know. Hmm, because it's. I would have to keep up with it. It's it's it involves more like, you know, it involves more keeping up with this is the experiment. And let's like, keep that sort of time bound like, OK, cool. If we have something that we're we're trying to test, then let let's make sure that we come back to it and we're like, we don't actually get to write it off and call it solved. We get to say, OK, cool. This is something to test. And like, let's come back and have a look and see did it actually solve the problem? So it's like problem solving at work, something I've got to like with something that I'm actually OK at doing at work, more or less, but it's something that I have to like. You know, drill into the uh, the the people that I manage is like alright, so problem solving isn't just come up solution. It's like you know coming up with potential solutions picking one to try and then like testing did the problem actually get solved. And and it it's the the it's the coming back to. It is the challenging part. The notebook is is. I don't know it it it feels like it's a a monumental task. I feel like I'm going to have like 50 things I'm trying to experiment with at. Any given time, but then like. In reality, like putting into practice, it's like, you know, it's two or three, actually. Yeah. But it just feels like it's going to be everything all at once that I'm trying to keep up with. But it's. It's it's not, you know, it's it's actually like, OK, cool. Let's try two or three things.  If say, even if there were, there were nothing else improved in in from our sessions here, something that I I recognize just from the act.  of like doing therapy. It is just something that I it's a skill that I had to develop from years ago was just coming to, you know, counseling. And just being honest and not trying to hide things and and and and just just having that sort of a an honest look at things like you know and making that a practice if even if it was like if we've we've done lots of of really you know great things and huge improvements. But just even if nothing else, just that practice of like you know. Let's come in and just be honest, completely honest. Once a week and not try and not try and sugarcoat things, not try and hide. Things just be like, OK, cool. Here we are. Is, yeah. So just sitting here thinking, but like, you know there there are are times when I've I've. I don't know. Just recognize that as it's just a a pattern and it's it's like something pulling a tool out of the toolbox, being like alright, cool. Uh, something I just, I don't know. I thought that I had thought that I recognized there. That was once very challenging, but it was less challenging now.  I think probably that comes from, you know, growing up with ADHD and, you know, having to come up with excuses why things aren't done or why didn't you do this or why aren't you living up to your why? And it's like ohh excuses, you know or I totally did do that. I just lost it. You know, and just having lying be a a a defense mechanism. A A way of of coping. Don't know if I've ever kind of put that those those together. Until just now. But but yeah Now that I've said it, it seems pretty obvious, but hey, that's OK. That’s here.  Actually it's it's something it's a problem I had with because it's actually you probably have very strong feelings about this is around the I think you call it like. The disorder model of this. That like having an idea of the neurobiology. My first reaction to that, or like my my first, I guess my life actions as a result of learning those was to be like, OK well, my brain works differently and that's my excuse. I guess that's just all of that's that's just how things are. And then like that. You know, OK, well, I guess I guess I guess just things are worse for me. And I'll just have to just make make peace with the fact that. You know that that's just this is where things are. Whereas in the sessions we've had here, it's been more about, OK, cool. Well, let's look at that and like, let's actually look at over overcoming some things or or you know using. You know, using the gifts that you have. Cool. I mean and and not even necessarily like but, but I get it. It's also not the same as some like like people with ADHD are able to use hyper focus or like like people that like it's not like seeing ADHD as superpowers either. It's like you know it it it's it feels like a more accurate. You know. Model to me and and in the way. And not not trying to like you know, just just blow smoke at you here, but just it just it. This is felt a bit more. A bit more real.  And the framework and the structure of this has been very helpful. And I I feel like what I would need to do to to maintain this would be to find. I guess to find a way of making these into habit or like like to to find a way of. Maintaining the framework, maintaining the structure and but like making that. Part super low effort. So that like so that the I can focus on the fun part of you know you.  Yes, absolutely. Looking back at like the the weekly, you know, and I don't have the results of those, but I I don't know if that like that particular data collection is. Going to be a super, super accurate representation of of like how useful I found this like I. But yeah, so I I feel like it's more useful than that thing might show. |
| 9 | Group 1 |  | Sharing useful for reflection  What is normal for ADHD  Validation is reassuring  Understanding others reactions | I really like them. I found them really useful. I think it's it's always like I'm I am quite a. I can say I'm a talker. I am like I am quite a lot of the talker. I think a lot of the time I talk to myself about most things good and and about things that are on my mind, I'll always. I'll always talk to myself about them and I think having someone other than myself to kind of be listening, me and. That sort of extra opinion. Will always like be really beneficial to like everyone. I think everyone would benefit from that and I think with the ADHD thing as well, I think it's like when you when you're having issues with something and being able to recognize that it's not just. An issue and that it is, you know, sometimes it is symptoms are like this. Whatever they call it, the ADHD I think that's always I can reassure not reassuring to know that it's not just. Like me being a drama queen or whatever and it. Is it is actual sightings that there's a reason why I've had that reaction and there's a reason why you're a typical people have had, like, different reactions. And I think that's always a very kind of comforting thing to know and to hear.  Because I don't believe in that the word overreaction cause like no reaction, like there's no standard reaction to anything so. It's always good to know that any kind like when neurotypicals as people call them have like a different reaction or whatever or it's good to know like what's. ADHD, showing its side and what's what's not essentially. I think that's all. It's always. Kind of like confirming. I don't know what the word is. I'm. Trying to think. Of but like. Validating. Yeah. Validating. Yeah, definitely. Yeah. That's it.  Maybe I think I'm a bit more understanding. I think if I'm having. Like if someones. Like when me and my. Mom were fully, like, butting heads at the start of the whole project. I think I was. Yeah, I think that I was in that kind of thing. I think you helped me be a bit more understanding about why she was being like that and about why she's so. About why she was being so like all the time. All like trying to always looking to like, try and. Understand why someone else is. React in that way behave in that way. Why they've done that and stuff like that, I think that's. I think I've definitely had. Like feel more aware of other peoples. Reaction kind of ohh if that makes sense. |
| 10 | Group 1 |  |  | Situation made it difficult to implement and experiment  Honestly, and it's not your fault. I don't think anything's really worked for me because of the situations I've been in. And there's huge gaps where I'd like to cancel or, you know, so I forgot, like, a lot of it. But you're the person. Yeah. Brilliant. Definitely. But like I said, I can't blame you because it's. Just one of them things. It's a lot. Has just got in. The way. So I've not been able to put my attention in it to actually try and learn. Yeah, like it was just. About time really. I couldn't turn down, you know, I mean, obviously anything that we could try and help you just gotta do it. 'Cause then it happened to be at the wrong time.  Yeah, definitely. Because obviously I wouldn't mind trying to again, you know, feel should be Obviously, I believe there might be a chance to probably work, so I mean cause I don't see why not, I can focus. You know what I mean so. It's just, I said. There's just too much around and you were too much to have time for myself. I mean, like, when I was pulling him. It's why you coming to work. That's because I've got my cancer healing here. |
| 12 | Group 2 |  |  | Great, I've I've learned. I've learned a lot of stuff that is. Helping me already and I think we'll once I I'm still not. Fully using all of the tools as a kind of. Habitually, as they would be more useful, but once they those are kind of fully in my in like a a fully habit like be great like I use the notebook like so much now and not as much but I've got the the cookbook stand and I've got the binder coming for it. But other than that, yeah, everything. It's been great. Been very useful, I think to talk through. A lot of the things.  I think a lot of the stuff I've kind of. That I I've learned about having an issue has been from very loose research and stuff I've seen on like social media and things like that like and having only been diagnosed a couple of years back. It's still fairly new. This is definitely been changed how I kind of think about. Going like like how I think about what's not attacking but approaching different things I need to. Kind of consider, I guess, brilliant. |
| 13 | Group 1 |  |  | Ohh to be fair, it's one of the things that. I thought were gonna be really. Onto the greater I thought this would be really tiring and maybe upsetting and just boring and. Come a bit more wrong. It's like I tell people about these sessions quite a lot how. Much of helped.  So I think it massively helps. It's massively helps me ******* so good. Yeah, I just there's not been a downside to it all. It's been just super helpful and easy to do and easy to talk to you and it helps me. Work out. Our minds working without feeling stupid about it, or feeling like it's something I'm just doing wrong. I'm just being bad at. More, which is that helped me deal with stuff I've been a lot happier. Unless. Down about stuff. So like. It's not that I overly get depressed through anyway, but like. Our a lot more up and down before than what I've been doing these. I think that's partly because I'm hating myself less for not getting stuff done. Yeah. Which then never helped anyway.  So I can't thank you enough for the help you give me, I. Really appreciate it. I think. This sort of thing should be more available for everyone we're testing. This has helped more than meds have than the meds have 10 times over. Yeah, definitely. It's been way more helpful than anything else. I really appreciate your help you've given me. |
| 14 | Group 1 |  | Internal feedback  Self-acceptance  Change in perspective  New self-narrative  Enjoyment of process  Self-understanding  New experience in counselling  Sense of peace  Improved sense of self  Felt listened to | Yeah, probably. Probably uncertainty. And I do have I want to release resentment as well and a bit of anger. I want to kind of get rid of those emotions. They're not really emotions, but they're reactions. So yeah. So I think, yeah, I gotta. I know kind of what I need to change. Inside and I've been put in situations this last year and last year where it's I think it's just a testament and just every year in every situation I need to pass the test until and I'll just repeating the same lesson. So I realize and then and that's it. So, you know, I think I think that. A little adventure will put me in situations where. Yeah, I I do. I do have insecurities. I do have jealousy and I want to get rid of those. They don't serve me at all anymore. And these situations have just shown me. Yeah. OK. Yeah, that's what you need to work on.  Yeah, understanding why I think. And then projecting it onto yourself. Changing what it is, be more receptive, I think, be more receptive to maybe a change in my narrative because that's the only place where you grow, isn't it? When you been challenged, you get challenged. And you also need to have the kind of. Understanding to change your perspective and change your thought process because you know. I am wrong. Thank you for showing me that. I'm now a better person. Instead of, you know, reacting with an ego or. Myopic stands, I guess so. Everything's a lesson and I want to learn more.  I've really enjoyed these actually.  Yeah, I can say without doubt about these sessions with you have got me a lot of kind of understanding and a lot of kind of peace as well and it's and it's lit up a few different avenues that I probably haven't got with any other counsellor before. Its opened up new kinds of brain networks and you know, I'm very much appreciative of those very much because I can say I'm a better person from having these sessions through you as a counsellor and through these sessions as well. So thank you. Thank you for listening. |
| 15 | Group 1 |  | Sense of options and choice to not overextend  Playing with approaches – adapting  Recognition of needs  Recognition of stress/anxiety levels and reduction  Tools linked to self-esteem and confidence  Negative interpretation of symptoms based on theory  Self-understanding  Share with others  Outstanding – big difference  Different from other support experiences  Accessible | Massively helpful - no downsides at all, super helpful and easy to do  No, it is going well. It's going really well. I can definitely see a change, just like I said the last time, just to thinking about it, the thought of it, you know, me sitting there and thinking about those tools that for me is really because that then gives me an option. You know, it gives me a good option and I feel like I'm not, which I'm not. My love, I'm being honest. I'm not overstretching myself anymore. Oh, I haven't been. For the last, oh, several weeks, I've not stretched myself, you know.  Yes, yeah. But that's human, right? Everybody occasionally has one of these things, right? It's the difference with ADHD is that we were having them all the time, right? Yeah. And if you get it down to kind of a lower level, yeah. Then that's brilliant. That's that's more sort of the the kind that everybody struggles with. Yeah. Which is why. Yeah, I mean the only thing I am struggling the most with, I would say which to begin with was doing well with Brad, I mean. I mean, I've I said to myself, I'll not use this rule because I'm not. It's not working, but I think if I can adapt it and get it when I need, it is again going back to that minute rule or two-minute rule is because it's I've sort of come backwards on that. But you know, if the summit here for example this green tea, I know full well if I don't put this back after I've finished you, I will leave it on this table. And I'll stay there cause Kerry will just turn and say well, not you move it. And I said yeah, I'll move it to one second, I'll come down and do it. Ohh 2 minutes, 4 minutes. You know. Yeah, that's the only. That's the only thing that I'm struggling with is again with the washing. You know, we can say, you know, I've got some washing and then I can take it down and do it. Quickly though, as I don't I I think Oh no, no, I'll do it. I will do it in a minute and I won't. I'm cancelling out that that few minute rule. And so I need to find a way to adapt it again to my needs that that are going to suit me, that I'm going to want to do. You know that I'm going to be interested and pick up on. It's about, like you said, it's having that that work rate down what that interest rate up, you know that's what I'm trying to get. You know, it's very difficult, very difficult. Keira is amazing at trying to remind that's why she sits in the sessions on and she can. Remind me if I need to. I said. Remember what Becca said. I mean, Rebecca said you're famous in my family, by the way. You're very, very famous in my things you love. Now everybody knows Rebecca and Rebecca champion. My family. They really do so. But yeah, it's going well apart from the odd struggle, like I said.  The stress levels have decreased slightly. I mean I do get for example, if I wake up, I walk up a bit late today because I was up quite a lot at night with the chess. I was in a bit of a mood, but obviously I was up late. I'm a bit groggy, but I'm realising, you know, I'm realizing say if Kira snaps at me and says no, stop being that way, you know, stop being that way. There's no need. I need to snap at kids or snap at me or don't do that beforehand. I'd be like, well, no, no, no, I'm right. No, no, no. But now. It's no I understand. Or I'll go back. I mean, there was some yesterday where I come back to you and said no, when you upstairs, I come back and say no, no, you're right. I needed to relax a little bit, you know? So I'm recognizing my own stress levels, my own anxiety levels, my own. I mean, even doing that thing beforehand, you know the questionnaire. I've noticed the odd things make because I have to remember always when I'm doing it is. It's within seven days. Yeah. Think of it as is. The question is 7 days. So what have I been like since in seven days from today from last week? You know what I've been like and I find sometimes, you know, I might rise again to to either to middle or to considerably I. But most of the time I I remember when I first started I was very anxious. Even doing the the form. It was considerably, considerably. And now I've seen the difference, you know, coming down, it's like my own little checklist.  I don't know if you know, but I do study these tools. I really do. I sit and I study these tools quite a lot because it's just trying to better yourself. That's all it is, isn't it? Just trying to better yourself. And I think that's important, especially for self esteem and for confidence. I think that's very important.  No, you definitely answered the question there. I mean the question pretty much was you know, is there gonna be more because I know full well what they tried to do to me with the diagnosed ADHD was send me to therapists, send me to CAMS and you know with these third sector organizations and they tried to do all this to me and I thought this was working at one point and. I started to feel well, no, I'm pretty much just being medicated and left and and being told that all it is that's in your brain is your brain's constantly just giving you the wrong sort of chemicals. And that to me wasn't. I think that's the reason that ADHD become A tag. That's the reason ADHD's become looked down upon, you know, and that's why I'm really I was always, I mean I was interested when I heard about you.  It's been it's been inspiring. Not just inspiring, but I've been able to pass things I understand. Obviously I I don't understand everything. But the things that I can understand and apply to myself, I can try and pass to others and that's all. It's about making. If I can make my or you can help me make my confidence higher and my understanding. Of of my own ADHD, you know I. Can help or hopefully just pass some the odd information to somebody else. You know, that's what it's about, I think  Wow, I bet you've put yourself under so much stress and you know that's for. That's for us. You know, that's what you know. I'm really serious. I mean, give us smiling there. You know, I've nearly got tears like the the help that you’re giving Rebecca was outstanding. I don't think you realize what difference you're making just through your research, let alone before it gets put into practice. The difference you're making for your research is everything, absolutely everything. Because I I will remember this for my life, I can pass this on out for the next 50 years.  You know, and I've only got plans for your paper. You know, I've got I've the photocopying it. I've been into libraries, I've print it out because, I mean, I think people should at least give it a go and and and think. Well if it's not working we can try some different here. That's the the evidence is showing. I mean for me it's concrete evidence. The only difference from the sort of people I've seen. You mean I've seen therapists. I've seen occupational therapists. I've seen psychiatry. I've seen every single kind of profession you can think. You're sat at home and your room. I'm sat here. In my kitchen. And you've made such a difference. You know, it's been outstanding, and not just for me. That means you made a difference from a partner. My children and I. Can. I honestly want to thank you from my heart. Brilliant. Thank. |
| 17 | Group 2 |  | Support helpful to prioritise chaos  Externalising  Circumstances impacted on implementation | I feel a little bit. Like cause my. Life has been so chaotic like, I mean, I guess you could describe my life as being chaotic always, but. Stance, but I think it's been helpful as well. You know, because things where we've gone over like prioritizing things and stuff, which has been helpful and it would be a bit bit of different if I was just doing a nine to five job would probably would have. Right. Got different things. Yeah, yeah, yeah. Help sort of talking about things and. Instead of just like leaving them in my head to think oh, oh, yeah, that was I needed to do like a lottery. And I know I. There's some things that I need to. Probably implement more, so there's things that we particularly when we first start. Talking. There's some things I did start to do, and then because of my. Well, it's just ironically, because of the ADHD. But I think it's also, it's also just not helped that I've been all around the place. Definitely, definitely. Just made it. In one place, I think it would be easier to sort of do some more routine things, yeah. |
| 18 | Group 2 |  | Change I outcomes  Neurobiological foundation helped with daughter as well  Understanding how to Motivate self to motivate child  Validation of daughter’s struggle | Outstanding - made such a difference for me and my family  I think it's probably going to be odd when it comes to the surveys at the end because a lot of what we discussed at the start isn't really the direction we went in. So we ended up talking a lot about my daughter and to be honest, that has been fantastic. I mean, it's been the most. Help. I mean, you know, I I know this was sort of signed up as a thing for me through the ADHD service, but oddly enough. It sort of turned into, yeah, just the most helpful thing for sort of really. Watching her dealing with her, sort of, you know, knowing or having a better idea of what's sort of going on, what processes she's going through. You know, just understanding how. You know that sort of cognitive function. A bit better and it's been really, really useful. Definitely. It's may I think it's may, it's sort of noticeable difference and it's sort of been a bit worried and they're kind of I don't know if this is going to have got what you needed out of it, but I mean certainly. Yeah, yeah. In in terms of. ADHD related coaching yes, it's been very, very useful.  The thing you know, sort of being used to my family, actually, that is useful to me. Because sort of. The the the main thing I do is. Sort of look after her. So also sort of knowing I think understanding a bit more of sort of when. 'm hitting a. Block and sort of knowing right. Why am I feeling like this that you know so when? She gets homework and just. I know her homework needs doing and I start feeling. It's like because I know you're going to need help. Doing your homework and I don't want to do. Absolutely. Yeah, definitely. And you know, even if it's just sort of finding like, what sort of little push do I need to be able to give you the push to do what you've got to do sort of whatever it is. So I think it's, it's useful just sort of having a more concrete thought pattern along. Those lines and going right. OK, how am I going to motivate myself to motivate you? Because frankly, I don't want to do any of that, so I can completely understand that you. Don't want to do any. Of that, and I can't even say to you. Because I don't want to be discovered. Yeah. Yes. This is a colossal waste of time. This feels like something simple this taking about. But that can that can actually be really useful.  And yeah, I think it it's just sort of knowing where my block is on it and going right. What can I do to get my pass me pass my blocks so I can get you past Your block on it. |
| 19 | Group 2 |  | Adjusting to circumstances with awareness of needs  Awareness of listening to self  Understanding of needs  Checking in with feelings  Shift in attention to resourceful approach  Stress reduction  Change in decision-making  Reduction in acting on impulse  Enjoyed the process  Useful  Self-reflection and exploration  Being listened to and experimenting with tools  New experience  Recommend to others  Benefits of self-reflection  Discovering value beyond rewards  Normalising difference  Positives as well as negatives  Independent self-esteem  Recognition of social misunderstanding  Self – appreciation vs self punishment  Curiosity about options  Self-forgiveness  And compassion  Reframing self-image/identity | think I think if if if I hadn't got, I got ill. I would I I would have probably have been venturing off more because I do like I I can. I can remember like thinking. A few weeks back or it like it potentially be quite cold, you know, like to wake up somewhere amazing dodge, you know, like or just wake up, like, really, really early on my birthday and Just. Like, drive to just like Mum tour or like something Watched like sunrise or like whatever and but obviously like with my lungs hurt and I was like. It's not advisable. It, and I suppose it just it just makes it less, less fun, you know, just like you just want to be able to. Walk around and breathe without it hurting. That that, that helps. Done it like, yeah. So like I. I was like. I thinkTo be fair, the way that that I actually handled it were. Really well, because I wouldn't even say that I would be disappointed. I was very, very much and like, look, it is what is what it is deep. These things happens. You're like it's nobody's fault. I can't change it. So it's fine. We'll do that another time. You know, like I don't. I don't need to join. Like, it's not like my life depends on it. You know, I've got. All time in world so. It's cool. We'll just we'll just have a nice relaxed day instead, and that's what. That's what I did. So yeah, I didn't. I didn't even get this. Disappointed, you know, like I was. I was aware that it was something that I'd like to. To do and, I was also aware you know that right now it's not something, you know that like. I wouldn't say that I'm not capable of because I I could, but I don't want to end up making my myself feel, feel, feel worse. I'm actually listening, you know, to. My body and what it's saying and I'm like, OK, so although you're like I can think and walk about and you're like I'm I'm. A hell of a lot better than than I want, you know, like I still clearly need. A bit of rest, you know to get Back to hundred 100% so. I think at one time. I'd have probably just done it and being like. It hurts when you breathe tough. ****. We're doing it anyway. So yeah, yeah, I think that's. That's different.  I think. I think I'm. I'm listening like to to my body more and I'm I'm able to. I'm able to understand. The things that that I want and understand why I want in those things, but instead of just acting on like impulse and just being driven by, you know that that kind of dope mean hit because that's what I like. And that's what I wantI'm able to, you know, to like, stop and think a little bit more and. Stop myself from just like acting instantly. And you know, I'm like, OK, so this this is what I want. How am I feeling? How is that going to make me feel? Do you know what kind of impact is that potentially gonna gonna have? Because I think. I'm a lot more aware now of like the the like. The whole kind of spectrum of my thoughts and feelings rather than it was very like tunnel vision before. I want this. Or is now you know like this this a lot more color to it that I'm I'm actually seeing in paying attention to and and I. Think I think. To be honest. Being no. Having having more of. Having more knowledge of, you know, like how how stress affects me, I think I think is is a massive driver for that. Because I think now. Now I'm so much more aware of that. I am. I am thinking more you know to to prevent unnecessary stress because I know you know that if I if I'd have pushed myself and. I did, you know, like that order. And like I I I climbed a mountain or or like whatever. Into like that would cause me stress because I I'd either get stressed at the fact that like I couldn't enjoy it as much as I wanted to, you know, because I was feeling pain, or I'd get stressed. You know, if, like the pain got too, too much and, you know, like, I'd be stressing about how to get back. You know I'm. I'm thinking a lot more now and I think that is due to just yeah, the the awareness that I've got of like this stressing. What that kind of does means for? For me, and I think you know seeing. Like being able to see and feel the difference as well. Do you know that's I can see the reward? You know from. Having a a less stressful life and time and I can see, you know, like how now? Like I can just chill out during like I'm not constantly during like thinking during like I'm not constantly during, like going like I'm I'm able to just. Give myself time. Enjoy this. This. There's so many positive things that have come from just. That that little little part of it. Stress. Yeah, yeah, yeah. The thing I think that that is that is a huge a huge driver definitely.  It's definitely made a massive difference to my decision making. Humongous sly. And it's. It's nice, you know, to like. Know that I I I am I am able to, you know like like you know stop myself and so yeah I still I still have you know like the instant thought of oh I want. To do this, but instead of just like going off on that tangent, I have that thought and then it's instantly OK. So how else am I feeling So it is, you know, just just having those like more impulsive and like thoughts of what like like my brain won't show. And it's like looking for that dopamine that happens and then the kickback is instantly. How else do you feel? Which that's that is becoming, you know, quite like in an instant thing. So I'm like, OK, so we we want to enjoy this and we don't want anything to cause us stress. So yeah. I am very much enjoying that. Yeah. Yeah, yeah, yeah. It's definitely been definitely been useful.  It's like it took to be fair. I I tried, you know, to to. To not have. Probably sounds like weird, but like expectations and like things because you know, this was something I'd I'd never, never done. So I came into it with like a very open mind, you know, just just kind of excited to to see, you know what, what would, what would come of it. And you know what it'd be like and nothing else. And. It's been absolutely amazing. Like I I have to say you and like I've I've enjoyed like. Every minute of it. And it's been so, so useful. Uhm, I think especially. For me, you know with like. The the kind of background and knowledge you know that I'd.  I'd already got, but then you know, like being able to just. Chat with you and you know, like just. A lot of things, you know, we'd we'd talk about that. You know, it would trigger my like thoughts into June. Like just stuff that I'd done with kids DBT wise and then being able to. To like tweak those because. A lot of those like like skills you know, were like embedded in my head because it's just something, you know, that was part of work that I was having to teach the kids. But then being able to like unpick those more. As well has been. That's that. That's been really, really useful to, you know, to just kind of tap into the. The knowledge that I've already got, but then there's so much. I feel that I've I've learnt as as well, you know, through just just through our our chats and I think. Being able to like, break stuff down. As well, you know it's, you know, in in it's like the the little kind of rewards the little wins and you know like taking little steps instead of big ones. You know there's there's there's so many aspects of. What we've like done and spoken about, because I I feel like when when we started. You know, there were, like, set things, you know, that we we we kind of targeted and then once once like I've got. Some things to implement there, then rest at time. It's it's just been doing like chatting about.  My just my experiences of like the week in Just like what I've done and you know how I've felt and you know, being able to reflect with another person has been very, very useful because I think, you know, like the first few few weeks. Gave me journal like really essential tools. To put like in my little tool belt, you know, to to kind of get me me off going and then it's just been, you know just. Running with it in Just, like trying stuff in Just, like keeping up stuff and then. Just, yeah. yeah, it's definitely been like. I mean, I've I've never experienced anything like this before and if. If anybody was was to ask me, I would like 110% recommend. I think the I I came into this year like thinking that well, I mean I knew you know that I did self reflect often you know because I am very, very curious. I'm very like a very curious creature and I think this. This is definitely June like show me how how important that self reflection is to know it's. I already knew it was, but I think I think it's definitely highlighted you know like. The benefits and stuff cause I don't I I don't think. It might sound weird, but I I don't think I only saw that before. Like I've I've I've just always been curious about why I've done something or why I've felt a certain way. I I don't think I've I've ever drawn like sat down like properly thought about. All the benefits that that, like brings, it's just something that I've like done, but now.  The difference being now is that you know like like the. The the the the importance of it is definitely you know, just just being highlighted and you know like. I can see. Those instant benefits of doing that, you know. So instead of just being like focused on journal like rights, how's that made me feel? Do you know I can see like beyond that? As well, and scenes, you know that like reflecting like I'm. I'm going to have a better understanding which you know is going to do XY and Z and you know I can see what benefits I'm going toGet from itBecause I think I've got a better understanding now. Of how do you know like to make my life easier? Do you know by these little benefits so? I know that like self reflection is is a benefit in itself, but then if I can highlight you know to myself doing this is you know it's. Picking out. The good even with stuff, you know that I do. Naturally, you know, so I don't want to. I know what I'm like. And I don't want to end up, you know, like getting in into that kind of routine of just, you know, like highlighting like the little benefits or rewarding myself for all the **** that I don't want to do because then. The way that I am. That definitely highlights that like that is something **** that I do not want to do and. I could end up whether it be during like a week, a month, Just like a year. That could end up turning into a massive roadblock. I am just rewarding myself, you know, so if. Yeah, I suppose it it is tricking my mind in a bit of like in in a way you know. So I'm not just like highlighting the rewards and benefits for the things that I'm not overly fond of doing. I'm also doing it for like the things that. I do like to do, you know and. Whether that's just highlighting, you know, like the benefits I'm getting out out of it, that's telling my mind that this is good. This is good. Yeah. So it just keeps that kind of rolling, if that makes any sense at all. Makes sense.  I I think like I'm I'm I think doing like less. I don't. I'm not seeing as much either. You know, you know that kind of well. I can't do that during like I think. I don't think I'm. I'm thinking about it as much because scheduling like the these going to be like ****. You know that like. I'm not as good at or doing like whatever, but. In everybody like that, do you know? So regardless of whether it's to do with like? My ADHD brain or or or not. We we're all different, you know, like and it's. We can't all be good at. Everything you know, like, there's so much that. I can do. That other folks are and these things that I can do. Like I might not be able to if it wasn't for the ADHD. You know, it's it. It's does, it's just kind of positives in that as well like since you're like good because I think it. I think. It is tough to like not to get like stuck on stuff because there is so much negativity around it and you're like it's it's, you know, like no like you can't do that that way. You know like you have to do it this way and that's and that's just because this is where. You know, like the the like non idiots brain has decided to like do it. But it's it's just those little things that he he even that himself it you know it it like highlights show that. You're thinking different, but then it's that person perceiving it as being bad, but it's not necessarily bad, you know, like it's it's just different and. I think it's recognizing that. If they want to see it as a negative or they want. To Judge Hun, that's your problem, not mine. Because I'm a lot more open minded and you know I can be cool with. Like, if you want to go like around in circle. Tools to get to like the endpoint, crack on jerk. Like if I want to do zigzags, that's cool tooThinks you know it's it's that. Just being being able to like understand how how much. How much other people should like perceptions, you know can? Really have an impact things you know, there's there's so many people that are just so narrow minded and they say is this naughty Boy syndrome. Well you can't have ADHD because I mean you're an adult and you're female so oh ****. I can't color. No. Sorry. My bad. I lied. You know. It's it's it. It it is just a lot of like naivety and lack of knowledge, you know and everything else from other people that I think can have, you know, like such a massive impact. Knows because you know, like people just don't don't know enough.  And now I think more than more than average, you know, like I am massively aware you know of like all these kind of negatives and and if else and that's that's on them though not not me just just just because. I want to do something this way. No, no, it does not mean I am wrong. It means, you know, like I'm doing something different. Or maybe you're doing it different. Have you thought about that? Do you know? Like, who? Who's to say that it's me. That's. I mean it different. You need to look and and analyze the way that you're doing it and why is it? Why is it me that's different? You know, so I think. I think more than more than ever, you know, like, yeah, I am. I am able to, you know, to to really sit and analyze, you know, like like all that kind of stuff and do like seed, you know, like all these negative thoughts and feelings. And you're like all that. What like. But then I'm I'm very much able to, you know, just. Not really. Care which I've I've not, I've not. I'm I'm not massively, massively bothered at what people think in many ways during like I've got like a very small circle like I I literally socialize with my dog, my partner and then my mum, my sister, my niece, you know, that's that's it. I've got a very, very small circle. And that's that's how how I like it. So it's not like I have. A lot. Of that, you know because. I I keep myself out at work as it's what makes makes me happy, but. Yeah, I can. I can't see it. And justAppreciate you know that I am the way that I am and you know, see. And think of. Even you know when it comes to things in like. The past that I'm able to look now and when at at that times you know I would, I would have beaten myself up and blamed myself. I can look back and see. It probably it might sound a bit ******, however I can see the problems in them which I couldn't before. Now you know so so I, but it's it's that whole like spectrum though again. When? Now you know it was very tunnel visioned. That it was me that was the problem. Now do you know there's more questions and you know, like it's a case of, like, I'm asking my so I'm like, is it like was it me though? Because what's? What made them think you know that they are doing it like the correct way? I'm doing it the the different way. Like what? Where's where's? Where's the manual that says you're like it has to be done this way in any way. Other than that, it's different, you know? Yeah, I am able to, like, think. A lot more about stuff instead of being like you're the problem. Yeah, which is. Also nice obviously.  I think like that in itself, you know, like it it it just it just helps you know to. To keep like spirits up. I suppose he. Instead of going into that instant Justlike. Self self blame. I'm kind of like. Just stop right there. Let me think about this and you know, so I'm. I'm not just absorbing like ****, you know, like like a sponge or, you know, like I'm, I think. I think in, in, in. I I am probably doing like without. Even realizing realizing it like. Going back in Just, like thinking of like things and you. Know forgiving myself. Which is just just that makes sense. I think there's so many things and so many times like over years that I've just taken the blame or. I've been doing like the one in the wrong and it's me. Me, me, me, me, me, me and never be. Mean in? A positive light. You know, it's always been me that is the problem. So I think. One thing that I have realized is although yeah during like now I'm on a much better track and you know like I'm in a much better place, you know, like and I've got all these skills, you know, for me to utilize and everything else and. This this still Just like a lot of forgiveness. Like past me needs, I think to, you know, like just to just to be able to to let go and. Move on and you know just. Give. Give myself, you know like. Now that I've got the knowledge like give give myself like the recognition. That I always need it, but I wasn't. Able to do before. Does that make sense?  Yeah, I think it's. Yeah, I think it's nice. Think sometimes you know, like you have to. You do have to. To revisit things, cause like whether whether you realize it or not like. All all our like experiences and things like, you know, the the all effect is they all shape us and you know like subconsciously. We're not always aware of how certain things have affected us. And you know, like we can, we can be doing things you know is is like a form of like trauma response and stuff and, you know, not even know, you know like in. I think. Was probably more than like most. We can we can experience something. And you know, like it can. It can be. Smallest thing it's, you know, like we might have have had. But experience in Just like be made. To feel stupid dodging, like be made fun of, you know, because we've done a certain thing. We don't forget that. Every time we go to do that same same thing, that thought instantly pops up and it's that, Oh my God. Like. I worked such aging like idiots, you know, like I was so embarrassed and. We relive it. Yeah, and I mean. That that thing could have happened like 10 years ago and we are still doing it, I think. I think you know that is white. You know, just just being able to like pick thing things out and grow it and and you know, be like, do you know what, it wasn't you. And it's OK because you know, like people, people don't. The knowledge maybe you know they don't have like the understanding that's all on them and you know, just just just being able to just leave stuff behind and, you know, just yeah, it's definitely I think I think it's definitely like a massive step, you know, to just. Just being able to. Stop that kind of cycle of doing something and you know, just really, really fun. It's very important, you know, to be able to. Just lay things to rest in it, I guess. |
| 20 | Group 1 |  | Adaptation of tools  Recognition of needs  Noticing variability  Awareness and curiosity around feelings  Confirmation of feelings and recognition of needs  Self compassion  Permission to choose | Imean, I know that I've kind of. I mean, I was like religiously. Following the weekly planner.  And I've kind of I still use that like I still have it my own kind of way of doing it. So but. I don't like religiously follow as much because I kind of don't need to. It's kind of like almost becomeI feel like that was a bit like a training wheels kind of deal, and now it's like, oh, well, I know what I'm doing. I know that. This goes here. And this gets written down so I don't use it as much, but this is definitely like. In this. Journal. This is like the oopsy. This is like the important one, but important things go down there and then I've got. A brain dump journal which has got everything in. So that's just random stuff. So that's a very good thing that's come out of it is. Being able to section off things and like, categorize everything I mean. I've I've been quite nice. Learn how to not dis my own likes and dislikes.  I do think that my. What do you call it? My filling out that thing every day. Well, I learned to do it before you asked. That's good. That took a. While it was, but I don't know, I feel like if you look at the. Results. It's very inconsistent. So, and that's just sort of what I am like, if I could like describe my 1:00. Personal and straight, it's very I am inconsistent with everything and it's hard to maintain that. So I think like my emotions are up and down. Like if I'm a bit hormonal, they're all going to be awful and they're all going to be like, you know, moderately or, you know, always and and but. Most of the time it's it depends.  Absolutely. I think I I think maybe that's something else. Well, and I don't, I think I've you've made it aware for me, but I do it quite a bit is I get given something and I'm like right, how can I? Make this easier. For myself. But then, if there's something that I actually really, really enjoy and I, how can I make this as? Complicated as possible. Was like, yeah. And I'm like, yeah. And I really enjoy doing it so. Like yesterday because I was ill and I just I came down here and I sat down and I was like, feeling really awful. I thought I was anxious because all night like, I felt like it was like adrenaline inside my tummy. It was horrible. And I was like, what is this? What is this?Feeling and I was feeling really ill. And then in the morning. Cause I thought I thought I was having like a a chilled out anxiety attack like I was so aware of it I was like. Why? Why, why? And I thought all right, so in the morning I woke up and I still had. That feeling. And then I was like well.  What am? I anxious about because I'm anxious about everything, but I've learned to deal with that. Why? You feel like this Now and then I thought, right, so went downstairs and I sat down, got all my work out. And for. I feel crap, like I feel awful. So I stood up and as I stood up. Because I was thinking. I'll go. Upstairs, I'm just going to go into bed. I'm going to take my laptop with me and just do work in bed. As soon as I stood up my hooked door go and John just came and went. I'm so ill and I went, oh, thank God. It's not anxiety. And I was like, I will too. I'm so relieved. And I was like, let's get in our arms. So when I sat, I spent the whole day because I felt so. So much more relieved than that. I I just felt a bit better, even though I felt. Was really crap. I felt happier and it was weird, like oddly happy. And it was quite nice to sit on a couch them. So for I have a free day and I can even chill on the couch and watch a lot of rings. Or I could do something. But I've not done because I'm so pressured with time, so I literally just taught myself how to use Indesign and I spent the whole day learning and actuallyWhere normally I would view that as you have so much work to do, you have so much, so much to do. You're wasting time trying to teach yourself this. You should already know this, and instead of doing that, I thought I'm going to take the day and do this. And I didn't feel like I. Was wasting it because I. Was ill so I feel like I've learned how to. It's like trick myself that was tricking myself, yeah But it was alright. |
| 21 | Group 2 |  | Useful for self-management  Engaging with small tasks vs overwhelm  Self-compassion | I think it has definitely been useful. I think it helped me like. Learn how to cope with some things.  I definitely wasn't like. Doing nearly asLike you know, just. Like just to. For example, just picking stuff and take it downstairs, or trying to do the smaller things so they just not get done. Very often. And now I try to do the small things instead of letting them all like pile up. Yeah, I feel good that I'm doing something at least. Yeah. So, like, I'm less. I'm more useful. At least I know it's not really like being useful or not, but that's just how I feel. More useful and better about myself.  It's still like a work in progress for me, but I think I'm better at it. I don't really know like. I just need to like I'm still like. You know the drawing thing is still like a day-to-day, like still trying to do it every day. And sometimes I don't, but I'm still trying anyway. And try not to feel bad if I don't so. It's just like you said. Like I said, just like every day trying. |
| 22 | Group 1 |  |  | COME BACK |
| 23 | Group 1 |  | Self-awareness and need recognition  Learning about self  Sharing knowledge with others  Disclosure  Self-confidence in self-knowledge  Ability to see where and how to learn more and develop | Really useful - increased awareness and reflection, using time tools and adapting to make easier  it's been. A bit of a. Roller coaster. Yeah, yeah. I mean, it's sort of been for the past 10 years. It's been. It's been a rollercoaster. I think that that's that's what. So nice about this, this sort of being able to go out and the gym thing that's that's a massive improvement. That's unbelievable. It's nice it's you know, it's it's obvious, it's measurable it's it's really good and. And just learning more. About why I am the way you know, with the way the way they do things, the way I need to plan things out before is is just amazing. And I've learned so much about myself and. And it it just makes things so much easier, which is really nice and and like. When when let's say Rs. RSD kicks in, I know what I know what that is and it and it you can rationalise it so much easier than. Of despair, which is really nice and. And then. It's been really lovely. To be able to help other people as. Well, so. I had an admit really. Really, genuinely one of the best experiences the other day I was meeting with the student who Mild mild safeguarding concerns and but he'd. They he basically has a lot. Of problems with his. Anger. And he's diagnosed with ASD, ADHD and dyslexia, and he literally just cannot control his anger at all. And when he was a child, he was taken out of school, institutionalized in behavioral centres to that to that level used to be really, really violent, proper bully. And in the last sort of year. He decided not to umm, and he's really struggling cause he's he's struggling with like the reputation still within and people don't trust him. And and I was obviously as as you do in safeguarding what you try and clarify information you try and buy. Just building trust and building relationships, talking about what they've got going on, and any any at one point said you know what? What really annoys me is is focus. I can't focus and I was like, OK, I was like, that's because you've got a diagnosis. Of ADHD isn't. It seems like, yeah, and I said. Well, I've got one. Too and then. Actually makes a staff member next to me. Who hasn't got ADHD? And she was she was like, the whole time. Like what? Really funny. And then we just. I just sat and talked to him and he's a 16 year old that can't read, can't write. Because of his of his ADHD. Quite emotionally intelligent lad though, like to be able to decide not to bully was was brilliant and and I just sat and I just talked to him about what it was and explained some of the different. Not symptoms, but the things that we can experience. And I said, you know, do you recognize yourself doing that? And he was like, yeah. And then he got quite angry. And he was like. Why is and, I explained dopamine and I went yes. So basically document is that it? Yeah that that's it. Did you miss? That's it. It is no. And he was like he was so cross because he's been working with. Yeah, he's been working with doctors his entire life, has been institutionalized. And he was like, nobody's ever told me that. Why has nobody told me that? And I was like, I was like, look, the, the, the, the science is new is new, but it and it's it's stuff that. Nobody ever told. Even more. People studying ADHD don't don't really fully understand yet, but you're right, you should have. You should have been taught and like I want ADHD. I was telling about you when I want ADHD therapy with an ADHD therapist who actually has idiots, obviously got ADHD and he just got really angry and I were don't worry it's been. Really scared to meet me all day and I've been running around anywhere so I I didn't get to meet until 1:00 in the afternoon and. He went he just out of nowhere, just turns with you and went. I'd have another one of these. Let us in that meeting with. Me and I was like Excellent, excellent. And it was just really, really nice. And I was like, I just felt like I really knew. What I was talking about and tastic good. Brilliant And is is . The the stuff in the next to me is the the head of that course, that whole curriculum area. So her knowing that as well is really nice because our our.  Faculty is is students that 90% of them have got some sort of scent. So really just and I just felt really proud. Of myself.  I'm really this this whole this whole process has just been amazing because I've, I've feel like I've. I I just understand so much and I. UM. I can see, you know, I can see what areas. Maybe not need improvement, but where I need help or where I need to learn more or. You know, just do do some more work and and and things that can get put in place, like in the workplace, which is really good. Yeah. Yeah. And that's really nice.  t's great. It's a fantastic tool. It's really, really good. Really good. All these little things you learn that it's. Like it's like. You know, you know when you learn like a sticky key on a keyboard. Like you learned that paste paste is what control the like. It's like that it's like Ohh secret hack. OK. |
