# Supplementary figures and images for: Correction: A Neuroaffirmative, Self-Determination Theory–Based Psychosocial Intervention for Adults With Attention-Deficit/Hyperactivity Disorder: Randomized Feasibility Study
Source: JMIR Form Res. 2025 Dec 3;9:e87679. doi: 10.2196/87679 (PMC12712557; doi:10.2196/87679)

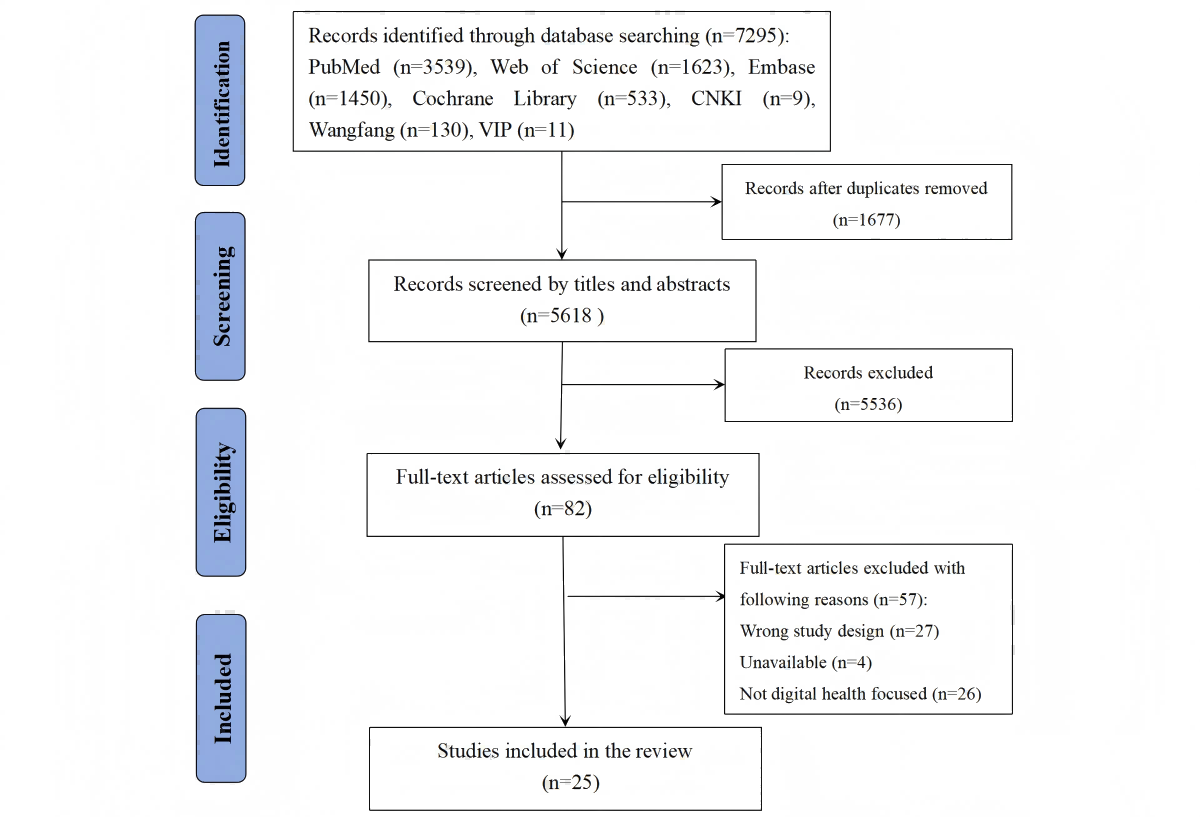

Supplement: Multimedia Appendix 1 [file formative_v9i1e87679_app1.png]
